# Genome-Wide Identification and Structural Analysis of bZIP Transcription Factor Genes in *Brassica napus*

**DOI:** 10.3390/genes8100288

**Published:** 2017-10-24

**Authors:** Yan Zhou, Daixiang Xu, Ledong Jia, Xiaohu Huang, Guoqiang Ma, Shuxian Wang, Meichen Zhu, Aoxiang Zhang, Mingwei Guan, Kun Lu, Xinfu Xu, Rui Wang, Jiana Li, Cunmin Qu

**Affiliations:** 1 Chongqing Rapeseed Engineering Research Center, College of Agronomy and Biotechnology, Southwest University, Chongqing 400716, China; zy18375729663@163.com (Y.Z.); 13594700414@163.com (D.X.); jld914@126.com (L.J.); 13108988211@163.com (X.H.); mgq12358@163.com (G.M.); 13699443694@sina.cn (S.W.); zmc201789@163.com (M.Z.); aoxiang922101@163.com (A.Z.); lingshifengshan@foxmail.com (M.G.); drlukun@swu.edu.cn (K.L.); xinfuxu@126.com (X.X.); ruiwang71@163.com (R.W.); 2 Academy of Agricultural Sciences, Southwest University, Chongqing 400716, China

**Keywords:** *Brassica napus*, bZIP transcription factors, expression patterns, phylogenetic analysis

## Abstract

The basic region/leucine zipper motif (bZIP) transcription factor family is one of the largest families of transcriptional regulators in plants. bZIP genes have been systematically characterized in some plants, but not in rapeseed (*Brassica napus*). In this study, we identified 247 *BnbZIP* genes in the rapeseed genome, which we classified into 10 subfamilies based on phylogenetic analysis of their deduced protein sequences. The *BnbZIP* genes were grouped into functional clades with *Arabidopsis* genes with similar putative functions, indicating functional conservation. Genome mapping analysis revealed that the *BnbZIPs* are distributed unevenly across all 19 chromosomes, and that some of these genes arose through whole-genome duplication and dispersed duplication events. All expression profiles of 247 bZIP genes were extracted from RNA-sequencing data obtained from 17 different *B*. *napus* ZS11 tissues with 42 various developmental stages. These genes exhibited different expression patterns in various tissues, revealing that these genes are differentially regulated. Our results provide a valuable foundation for functional dissection of the different *BnbZIP* homologs in *B*. *napus* and its parental lines and for molecular breeding studies of *bZIP* genes in *B*. *napus*.

## 1. Introduction

The basic leucine zipper (bZIP) family, one of the largest transcription factor (TF) families in plants, is widely distributed in eukaryotes [1]. The bZIP TFs play a crucial role in various biological processes in plants, including signal transduction, stress responses, and growth and development [2]. During these processes, the expression of bZIP TF genes is primarily induced by abscisic acid (ABA), an important phytohormone that functions in the abiotic stress response that regulates the expression of numerous genes and associated physiological processes [3]. The *Arabidopsis thaliana* genome encodes 13 bZIP TFs in the A subfamily, including ABI5 (ABA Insensitive 5) and ABFs (ABA-responsive Element Binding Factors), also known as AREBs (ABA-responsive Element Binding Proteins), which play pivotal roles in activating plant ABA signaling [4,5,6,7]. In addition, bZIP TFs are crucial regulators of drought, high salinity, and cold stress responses in many plants, including *Arabidopsis* [8], *Oryza sativa* (rice) [9], *Triticum aestivum* (wheat) [10], *Solanum lycopersicum* (tomato) [11,12], *Glycine max* (soybean) [13], and *Capsicum annuum* (pepper) [14]. Many bZIP TFs also play a crucial role in plant growth and developmental processes, including organ and tissue differentiation [15], cell elongation [16,17], nitrogen/carbon and energy metabolism [18,19,20], the unfolded protein response (UPR) [21,22], seed storage protein gene regulation [23], and somatic embryogenesis [24]. Therefore, bZIP TFs play a crucial role in protecting plants from all types of biological and abiotic stresses.

*Brassica napus* (AACC, 2*n* = 38), one of the most important oilseed crops worldwide, originated from hybridization between *Brassica rapa* (AA, 2*n* = 20) and *Brassica oleracea* (CC, 2*n* = 18), which have a common ancestor [25]. At the most recent, moreover, they have experienced α and β duplication events, and then they have two triplication events during the specific to *Brassica* clade during the evolutionary process [26]. For these whole-genome duplication (WGD) events, along with the merger of the two progenitor genomes, have resulted in a large number of gene duplications in the *B*. *napus* genome, followed by substantial gene loss [27]. Further, the A/C genomic sequences of *B. napus* had a good collinearity with the A and C genomes of *B. rapa* and *B. oleracea* with three sub-genomes showing different levels of fractionation (least fractionized subgenome (LF), moderately fractionized gennome (MF1), and most fractionized genome (MF2)), which were also extrapolated to the *B. napus* genome [25]. Thus, *Brassica* is regarded as an ideal model for investigating polyploidy evolution [25]. To date, many bZIP TF genes have been identified and characterized extensively in various plants, including *Arabidopsis* [7], rice [1], sorghum [28], *Zea mays* (maize) [2], *Vitis vinifera* (grapevine) [29], *Cucumis sativus* (cucumber) [30], *Ricinus communis* (castor bean) [31], and *Hordeum vulgare* (barley) [32], for which whole genome sequences are available. However, genome-wide surveys and expression analyses of this gene family have not been performed in *B. napus*.

In this study, we screened the draft sequence of the *B*. *napus* genome (http://www.genoscope.cns.fr/brassicanapus/) for bZIP TF genes. We identified 247 *BnbZIP* genes and performed a detailed analysis of their duplication and classification, chromosome distribution, and motifs, as well as phylogenetic analysis. Finally, we verified the differential expression profiles of selected *BnbZIP* genes in 17 different *B. napus* tissues at various developmental stages. This study provides important information about the origin and evolution of the bZIP family in *B. napus*, and lays the foundation for further studies of the functions of the bZIP family in this important crop.

## 2. Materials and Methods

### 2.1. Identification of bZIP Family Genes in Brassica napus

Based on the protein sequences of the bZIP family from the The Arabidopsis Information Resource (TAIR) database (ftp://ftp.arabidopsis.org), 247 *B. napus* bZIP protein sequences were obtained as query sequences through BLASTp searching [33] against the *B. napus* genome database. The candidate sequences were chosen based on an E-value of ≤1 × 10^−20^. The candidate sequences were further confirmed using the HMMsearch program (HMMER 3.0, http://hmmer.janelia.org/), and BLAST analysis of the bZIP sequences in the *B. napus* genome database was performed using Geneious 4.8.5 software (http://www.geneious.com/; Biomatters, Auckland, New Zealand). The candidate genes were named using two-letter abbreviations (italicized) denoting the source organism, the family name, and the positions in the subtribe, e.g., *BnbZIP1a*. The lengths of the coding sequences (CDSs) were identified by performing BLASTn searches against the *B. napus* genome database. The physicochemical properties of the deduced proteins, including the molecular weight (MW), isoelectric point (pI), and grand average of hydropathy (GRAVY) value were determined using the ExPASy-ProtParam tool (http://web.expasy.org/protparam/).

### 2.2. Multiple Sequence Alignment and Phylogenetic Analysis of the bZIP Family Members in Brassica napus

*Arabidopsis* contains 75 putative bZIP genes [7]. The 73 authenticated *Arabidopsis AtbZIP* genes were used for multiple protein sequence alignments using ClustalW2 software with default settings (AT2G12980 [*AtbZIP32*] and AT2G13130 [*AtbZIP73*] are not available) [34]. The phylogenetic trees were constructed using Molecular Evolutionary Genetics Analysis (MEGA) 6.0 with the neighbor-joining (NJ) method, the JTT+I+G substitution model with 1000 bootstrap replicates [35]. The phylogenetic trees were visualized using FigTree v1.4.2 (http://tree.bio.ed.ac.uk/software/figtree/).

### 2.3. Analysis of Exon-Intron Structures and Conserved Motifs in BnbZIP Genes

The DNA and complementary DNA (cDNA) sequences of the putative *bZIP* genes were downloaded from the *B*. *napus* genome database, and the exon-intron compositions of the *BnbZIP* genes were analyzed using Gene Structure Display Server (GSDS) (http://gsds.cbi.pku.edu.cn/index.php). The conserved motifs of *BnbZIP* family members were determined using Multiple Em for Motif Elicitation (MEME) 4.11.4 (http://meme-suite.org/tools/meme), with the following parameters: number of repetitions, any; maximum number of motifs, 15; and optimum width of each motif, between 6 and 300 residues [36]. Only genes with an E-value of <1 × 10^−20^ were subjected to further analysis.

### 2.4. Chromosomal Distribution of BnbZIP Genes in Brassica napus

The detailed chromosomal locations of each *BnbZIP* gene were mapped to the *B*. *napus* chromosomes according to the GFF genome files downloaded from the *B*. *napus* genome database. The physical chromosome maps were drafted with MapChart 2.0 [37], and the *BnbZIP* genes were graphically displayed on the *B*. *napus* chromosomes.

### 2.5. Analysis of BnbZIP Gene Expression Patterns

Transcriptome sequencing datasets were deposited in the BioProject ID PRJNA358784, which were sequenced by RNA sequencing (RNA-seq) from 17 *B*. *napus* cultivar Zhongshuang No. 11 (ZS11) tissues (roots, stems, leaves, buds, anthocaulus, sepal, petal, pistils, stamens, anthers, filaments, the top of main inflorescence flowers, seeds, embryos, seedcoat, funiculus, and silique pods) at different developmental stages. Total RNA was isolated using an RNAprep Pure Plant Kit (Tiangen Biotech, Beijing, China) according to the manufacturer’s instructions. RNA-seq library construction and data analysis were performed as described [38]. All *BnbZIP* genes expression levels were quantified in terms of FPKM (fragments per kilobase of exon per million mapped fragments) using Cufflinks with default parameters [39], and then extracted from RNA-seq results according to their *B*. *napus* code. The heatmaps for the *BnbZIP* genes were drafted using HemI 1.0 software [40].

### 2.6. Quantitative Real Time PCR Validation of Transcriptome Data

To confirm the RNA-seq data and to characterize the expression patterns of *BnbZIP* genes involved in *B*. *napus* development, the expression profiles of 13 genes in different ZS11 tissues and different subfamilies (as determined in this study) were evaluated by quantitavite real time PCR (qRT-PCR) analysis. The cDNA was synthesized with the Oligo dT-Adaptor Primer, using an RNA PCR Kit (AMV) Ver. 3.0 (TaKaRa, Dalian, China). qRT-PCR analysis was performed on a Bio-Rad CFX96 Real Time System (Bio-Rad Laboratories, Hercules, CA, USA) with the following cycling conditions: pre-incubation at 95 °C for 2 min followed by 40 cycles of denaturation at 95 °C for 10 s, annealing at 60 °C for 20 s, and melting curve analysis [38]. *BnACTIN7* (EV116054) and *BnUBC21* (EV086936) were used as internal controls to evaluate relative gene expression levels [41]. Target genes and internal controls were amplified in single wells in triplicate, and the expression levels were calculated by the 2^−ΔΔCt^ method. The values represent the average ± standard deviation (SD) from three independent biological replicates. All the primers were listed in the Supplementary Materials Table S1.

## 3. Results

### 3.1. Identification of bZIP Family Members in Brassica napus

**Figure 1:**
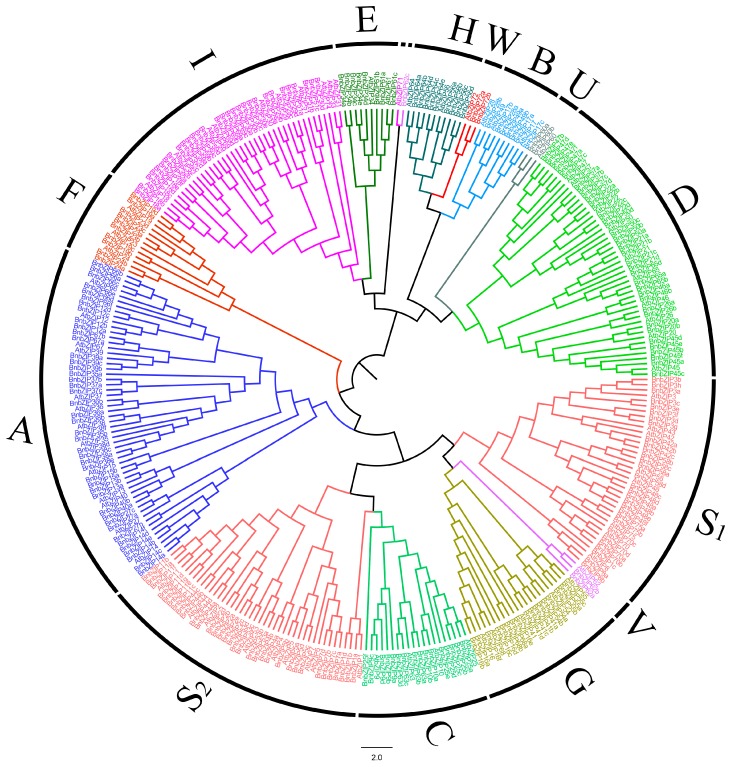
Phylogenetic analysis of the *B. napus* and *Arabidopsis* bZIP proteins. The protein sequences from 247 bZIPs from *Brassica napus* and 73 bZIPs from *Arabidopsis* were aligned using ClustalW. The phylogenetic tree was constructed with MEGA 6.0 using the neighbor-joining (NJ) method with 1000 bootstrap replicates. All bZIP TFs were divided into 13 subfamilies, A–I and S–W. The subgroups A-I and S were named according to Jakoby et al. [7], and U, V, and W were the new subgroups, with similar to *AtbZIP60*, *AtbZIP62*, and *AtbZIP72*, respectively.

In the model plant *Arabidopsis*, 75 bZIP members have been identified [7], however, the sequences of *AtbZIP32* and *AtbZIP73* were not accurately identification in the *Arabidopsis* genome database. To perform genome-wide identification of bZIP proteins in *B. napus*, therefore, we used BLAST and the Hidden Markov Model (HMM) profiles of the bZIP domain to screen the *B. napus* genome database. We identified 247 proteins with bZIP domains in the *B. napus* genome. A subset of the genes encoding these proteins are homologous to 67 of the 73 *Arabidopsis* bZIP TF genes, except for *AtbZIP6*, *AtbZIP31*, *AtbZIP33*, *AtbZIP43*, *AtbZIP71*, and *AtbZIP74*. We named these genes *BnbZIP1* to *BnbZIP75*, corresponding to the respective *Arabidopsis* genes (Figure 1, Table S2). In addition, 2–8 copy members were identified from 67 *BnbZIPs*, respectively (Table S2). For example, twenty *BnbZIP* genes only contains two members, *BnbZIP3* have 8 members, and fewer than 7 members of the other *BnbZIP* families were identified (Table S2). These results are consistent with the finding that excessive gene loss typically occurs after polyploidization in eukaryotes [42,43]. These bZIP TF genes encode proteins with deduced amino acid sequences ranging from 82 (*BnbZIP16f*) to 729 (*BnbZIP1c*) amino acids in length, with molecular weights of 8.79 kDa (*BnbZIP16f*) to 79.69 kDa (*BnbZIP1c*). The isoelectric points of these proteins were predicted to range from 4.74 (*BnbZIP60b*) to 10.35 (*BnbZIP14c*). The GRAVY values for all 247 *BnbZIP* proteins were found to be negative, indicating that they are hydrophilic, which is essential for the functioning of TFs. The gene names and related information are presented in Table S2.

### 3.2. Phylogenetic Analysis of the bZIP TFs in Brassica napus

The 75 bZIP TFs identified in *Arabidopsis* were classified into 10 groups, of which one group contains three bZIP TFs (*AtbZIP60*, *AtbZIP62*, and *AtbZIP72*) [7]. To explore the evolutionary relationships between bZIP TFs from *A*. *thaliana* and *B*. *napus*, we performed a phylogenetic analysis of the 247 BnbZIP TF protein sequences identified in *B*. *napus*. All 247 BnbZIP TFs were classified into 10 groups (Figure 1), which we named A–I and S, as in *Arabidopsis*, and the genes with high levels of sequence similarity to *AtbZIP60*, *AtbZIP62*, and *AtbZIP72* were named U, V, and W, respectively. Group S, the largest group, includes 63 *BnbZIPs*, which were further divided into two subgroups (Group S_1_ and S_2_). However, the *AtbZIP1*-containing clade were grouped as part of C-Group (Figure 1), which were different with the previous research [7]. Groups A and D contain 43 and 37 members, respectively. Group I contains 30 members. Groups C and G contain 16 and 17 members, respectively. Groups B and H contain 7 and 9 members, respectively, and Groups U and W each contain 2 members. Finally, Groups E, F, and V include 8, 10, and 3 *BnbZIPs*, respectively. However, Groups C, G, and V are contained within Group S, and *AtbZIP71* and *BnbZIP62c* were classified in the same subgroup (Figure 1). These results indicate that the conserved areas have been preserved of these genes share similar evolutionary relationships during evolution between *Arabidopsis* and *B*. *napus*, but several variations have also occurred, enabling the division of some genes into subfamilies, e.g., *AtbZIP1*, *BnbZIP1a*, *BnbZIP1b*, *BnbZIP1d*, *AtbZIP71*, and *BnbZIP62c* (Figure 1).

### 3.3. Gene Structure and Motif Analyses of BnbZIPs

**Figure 2:**
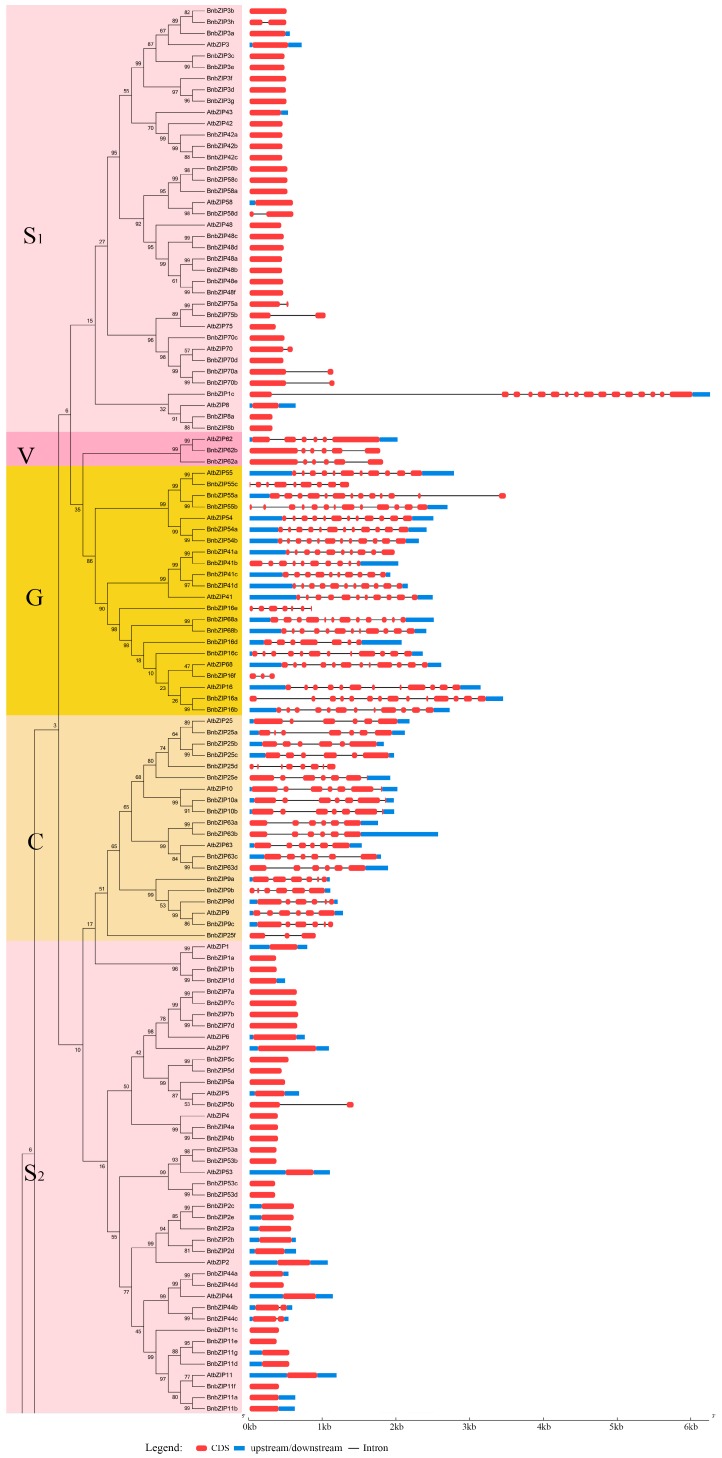

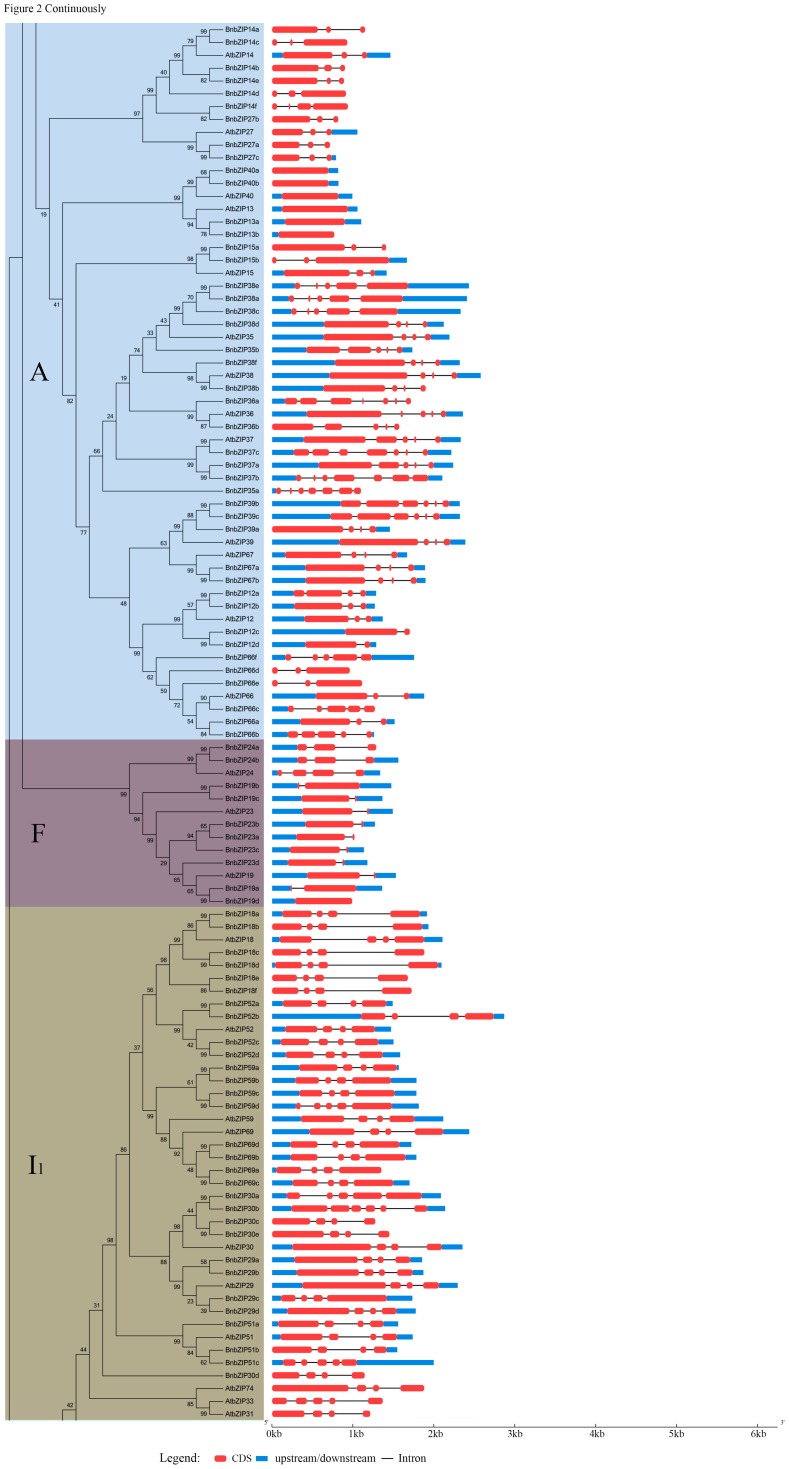

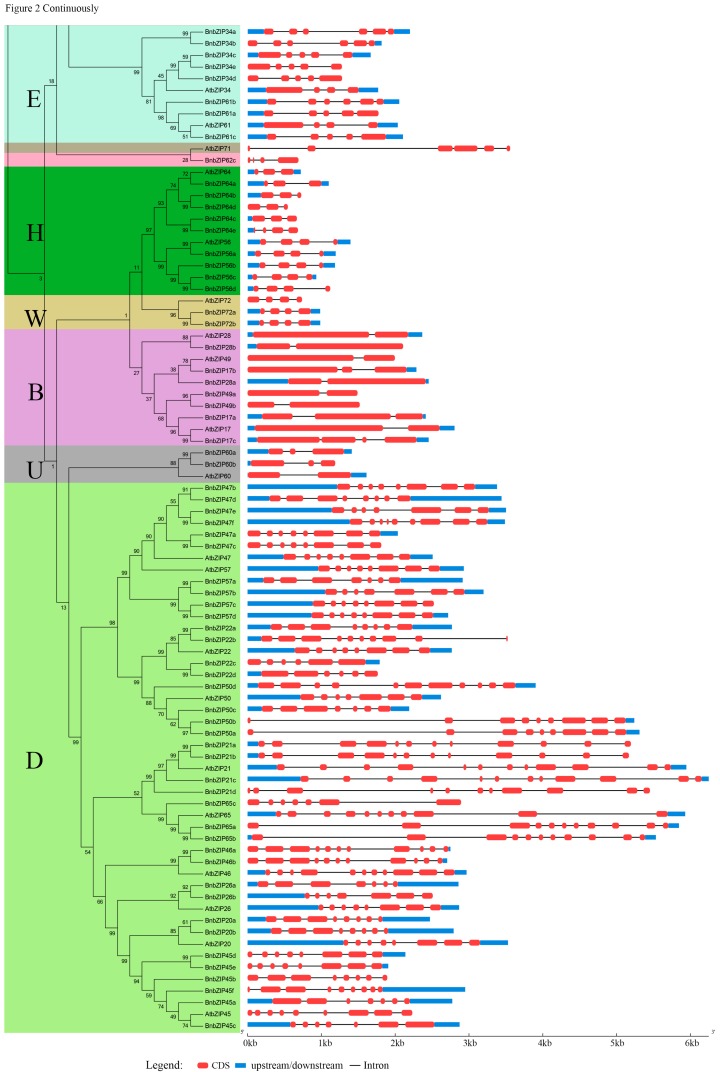
Sequences analysis of exon-intron structures of the *BnbZIP* genes. The red boxes indicate exons. Solid lines indicate introns (connecting two exons). Untranslated regions (UTRs) are indicated by blue boxes. The length of *BnbZIP* genes were indicated by horizontal axis (kb).

To explore the structural diversity of the *BnbZIP* genes, we analyzed their intron-exon structures using GSDS. The number of exons varies from 1 to 16, indicating a high degree of divergence among the 247 *BnbZIP* genes (Figure 2, Table S2). However, the exon-intron structures of the genes are more highly conserved within a single subgroup. For example, most genes with uninterrupted open reading frames (ORFs) are found in Group S (Figure 2, Table S2). However, among the genes in Group S, *BnbZIP1c* has 16 exons, while *BnbZIP3h*, *BnbZIP5b*, *BnbZIP75a*, *BnbZIP75b, BnbZIP44b*, *BnbZIP44c*, *BnbZIP58d*, *BnbZIP70a*, and *BnbZIP70b* each have two exons (Figure 2, Table S2). Additionally, genes in Group D and G have 5–11 and 2–13 introns, respectively, indicating that some subgroups are highly divergent. We also identified highly paralogous pairs among the *BnbZIP* genes with relatively high bootstrap support (>99%), such as *BnbZIP41c–BnbZIP41d*, *BnbZIP16a–BnbZIP16b*, *BnbZIP27a–BnbZIP27c*, *BnbZIP12a–BnbZIP12b*, *BnbZIP22a–BnbZIP22b*, *BnbZIP30a–BnbZIP30b*, and *BnbZIP69a–BnbZIP69c*.

**Figure 3:**
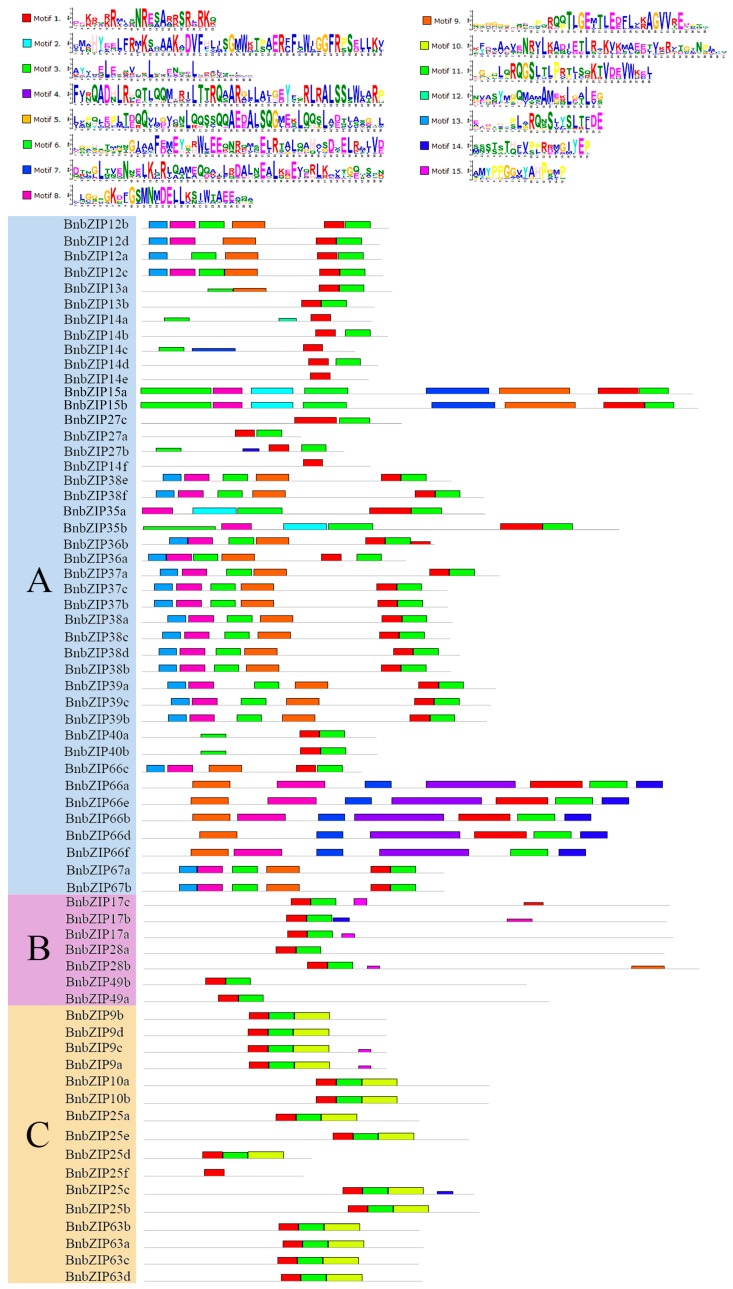

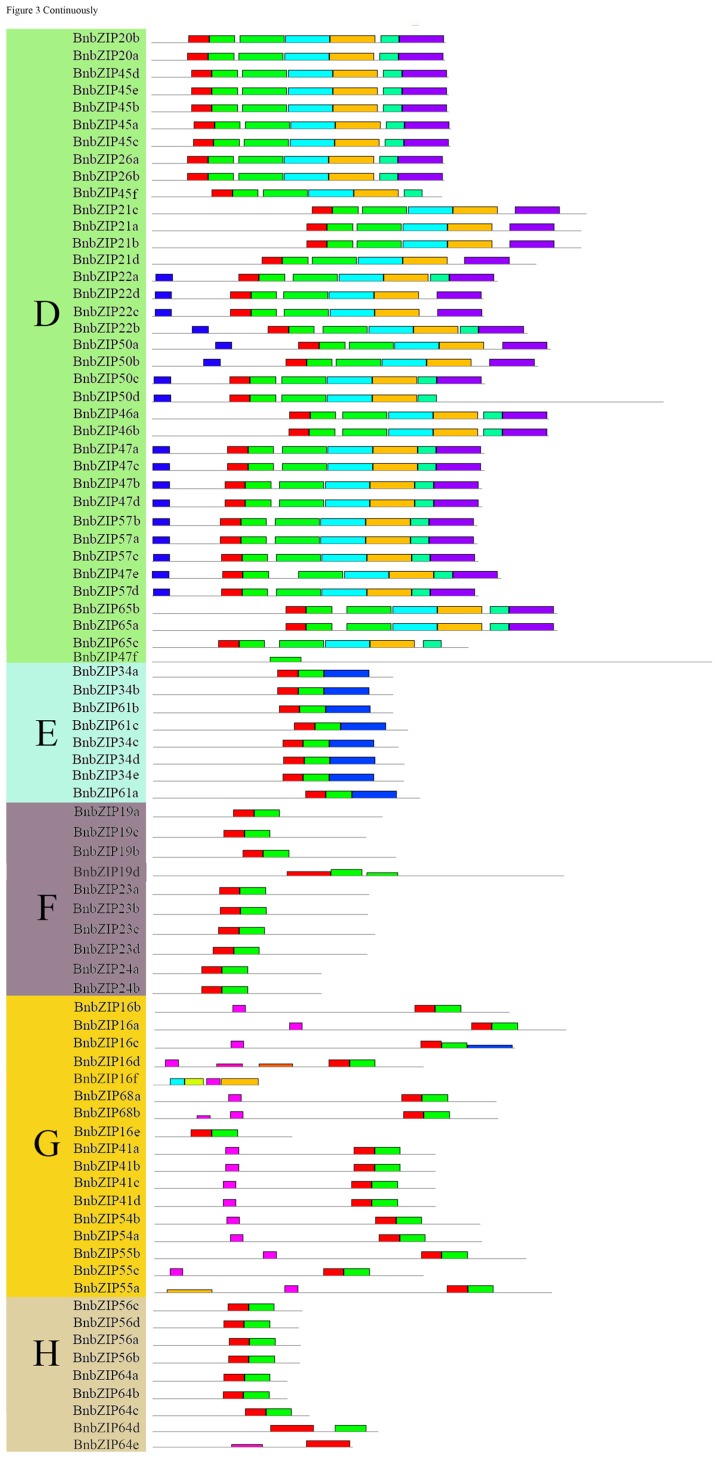

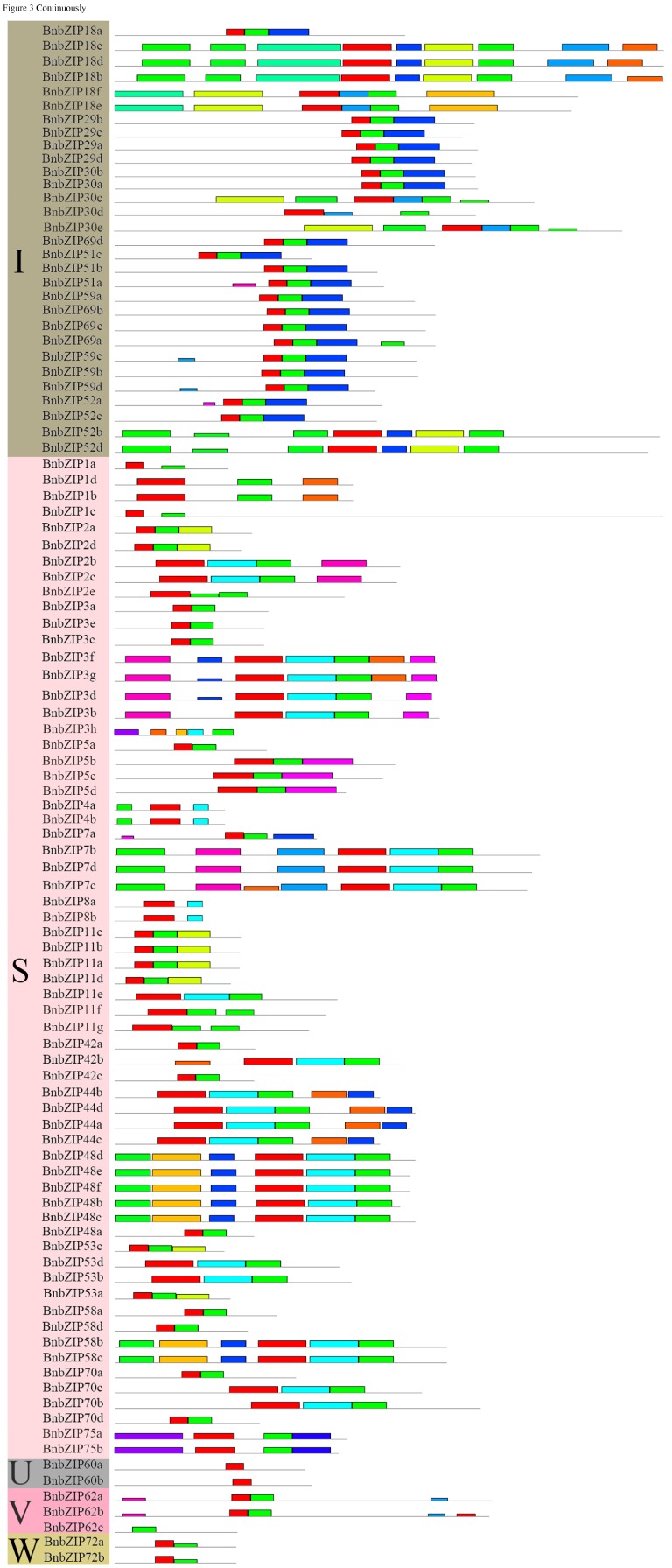
Motif patterns and WebLogo plots of the different *BnbZIP* subfamilies. The 247 *B. napus* BnbZIP amino acid sequences were aligned (Figure S1). The motifs are indicated by different colored boxes (numbered Motif 1–15).

As shown in Figure 3, one or more identical motifs (motif 1–9) are shared among members of the same family, with major differences detected among groups. For example, motif 1 and motif 3 are widely present in all *BnbZIP* members; motif 1 encodes the conserved bZIP domain, with a basic region of 16 amino acid residues containing a nuclear localization signal followed by an invariant N-x7-R/K motif [7], which binds to the DNA (Figure 3). We detected widespread variation in the motif pattern among Groups A, D, I and S; for instance, motif 2 was found in Groups A, D, G and S; motif 4 and 5 were found only in Groups I and D; motif 7 was found in Groups D, E, and I; motif 8 was found in Groups A and S; and motif 9 was found in Group A (Figure 3). These results showed that the composition of the structural motifs varies among different bZIP families but is similar within the same families, and that the motifs encoding the bZIP domains are conserved.

### 3.4. Genomic Distribution of the 247 BnbZIPs on the 19 Brassica napus Chromosomes

**Figure 4:**
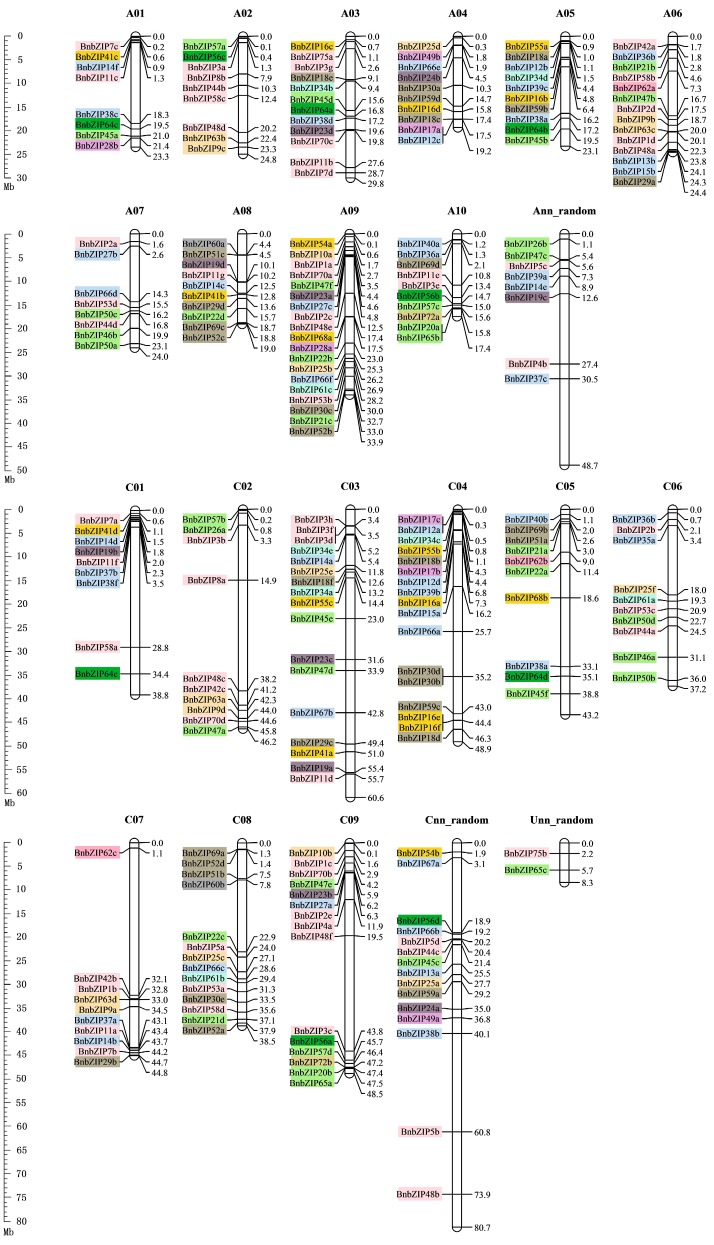
Distribution of *BnbZIP* gene family members on the *B. napus* chromosomes. The 222 *BnbZIP* genes for which exact chromosomal information is available in the database were mapped to the 19 *B. napus* chromosomes. Genes from the same subtribe are indicated by the same color, which is consistent with the corresponding family in the evolutionary tree. The scales were indicated the genome size of *B. napus* genome (Mb). A01-A10 and C01-C09 indicate the chromosomes of *B. napus*. Ann_random: unmapped A Chromosomes of *B. napus* genome; Cnn_random: unmapped C Chromosomes of *B. napus* genome; Unn_random: unmapped Chromosomes of *B. napus* genome.

Based on the physical positions of the *BnbZIP* genes annotated in GFF files, 222 *BnbZIP* members could be accurately mapped onto the 19 *B*. *napus* chromosomes, whereas the others were located on the unmapped scaffolds in the *B. napus* genome (Figure 4). Furthermore, all *BnbZIP* members varies considerably among the *B*. *napus* chromosomes, with chromosomes harboring from 8 (A01 and A07) to 19 (A09) of these genes (Figure 4, Table S2). *BnbZIP* genes are mainly localized on chromosomes A06, A09, C03, C04, C08, and C09, which contain 14, 19, 17, 17, 14, and 15 *BnbZIP* genes, respectively (Figure 4, Table S2). In addition, many *BnbZIP* genes are distributed near the ends of chromosomes. We detected 15 tandemly duplicated genes (with two or more homologous genes ≤100 kb apart) on eight chromosomes (A04, A05, A08, A09, A10, C03, C04, and C09). Three of the 13 subclusters, including Groups A, D, and S, are widely distributed throughout the *B*. *napus* genome (Figure 4).

### 3.5. The Differential Expression Analyses of the BnbZIP Genes in Various Tissues of Brassica napus

**Figure 5:**
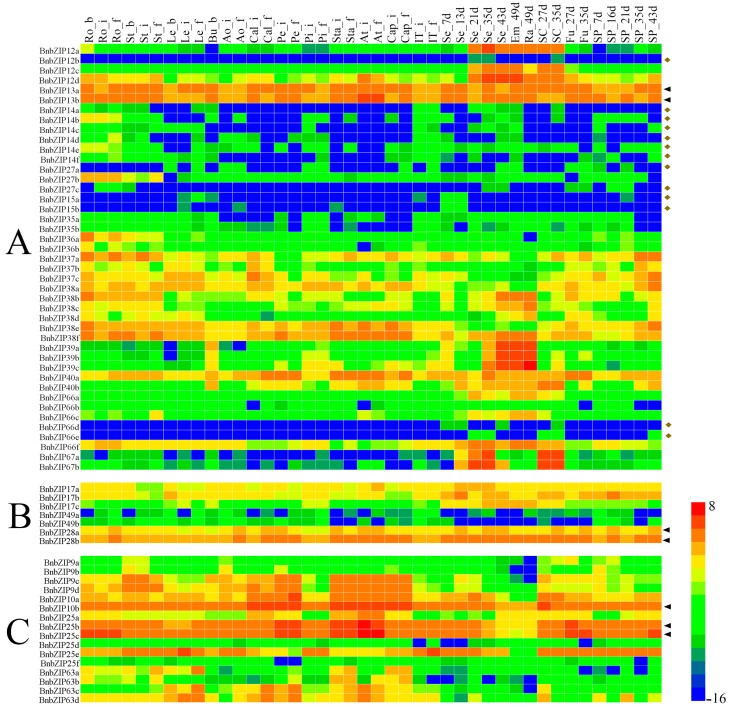

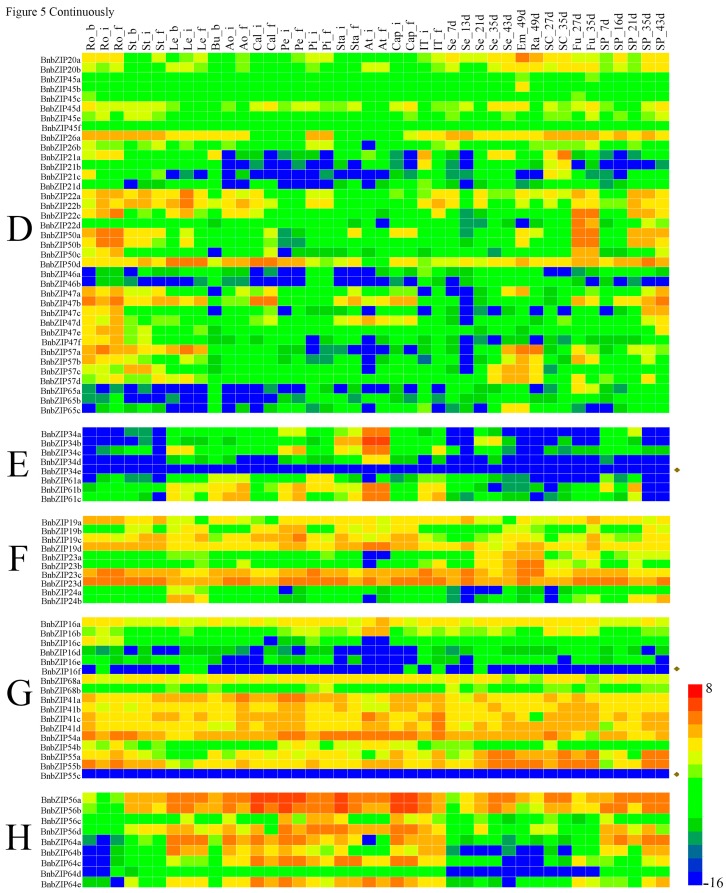

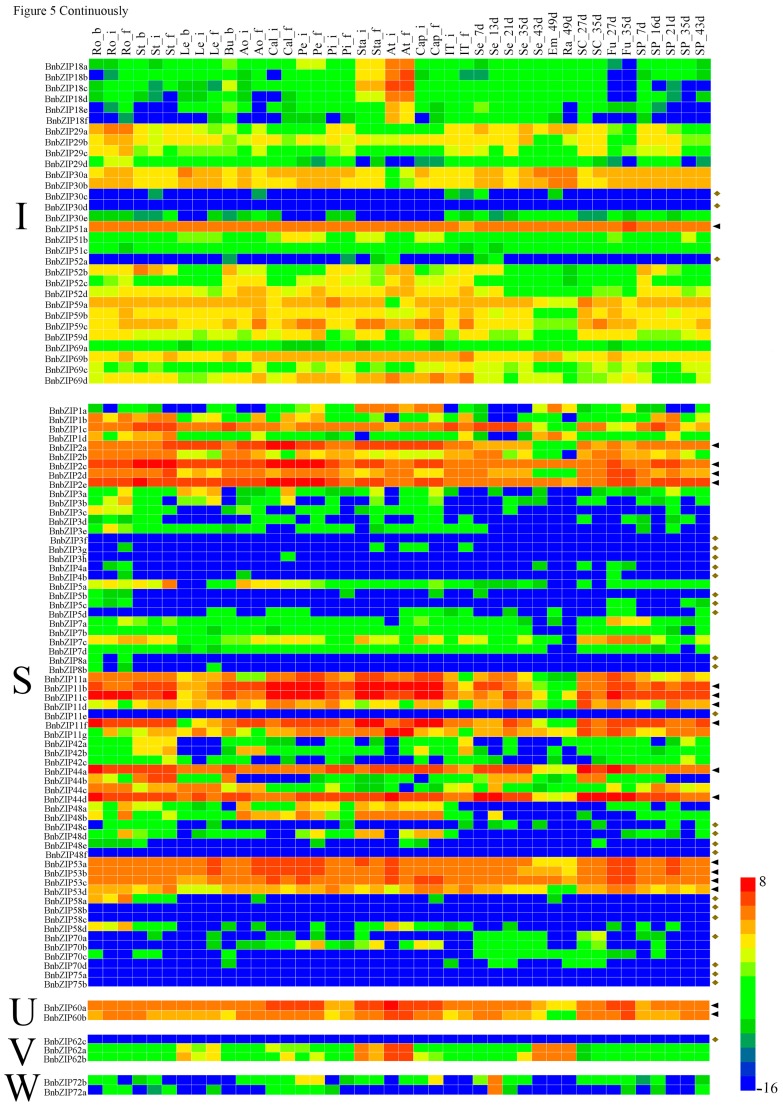
Heatmap of the expression levels of *B*. *napus bZIP* transcription factor family genes during different periods of development in different tissues and organs. Each subfamily is represented by a different uppercase letter (A–I, S, U–W). The black triangles correspond to higher expression levels in all tissues during the whole development, and the yellow diamonds correspond to lower expression levels in all tissues during the whole development. The abbreviations combinations above the heatmap indicate the different tissues and organs/developmental stages from *B*. *napus* ZS11 (listed in Table S3). The “scale” function in R was used to normalize relative expression levels (*R* = log2/FPKM). The heatmap was generated using Heatmap Illustrator (HemI) [40].

We investigated the expression profiles of the 247 *BnbZIP* TF genes in different tissues at various stages of development based on RNA-seq datasets from *B. napus* ZS11. In this study, we generated high-quality transcriptome sequencing datasets (BioProject ID PRJNA358784) from 17 *B. napus* ZS11 tissues at different developmental stages, e.g., roots, hypocotyls, cotyledons, stems, leaves, buds, flowers, and seeds (Table S3). We then calculated the relative expression levels of the 247 *BnbZIP* genes based on the RNA-seq data, which we used to construct a heatmap. In the present study, the results showed that the expression profiles of *BnbZIP* genes were associated with different tissues, and the expression patterns also differed among each *bZIP* gene family (Figure 5). For example, genes in the largest Groups, A, D, and S, had significantly different expression levels in all tissues and organs. Most *BnbZIP* genes in Groups C, F, G, H, and U were highly expressed in all tissues and organs, whereas most genes in Groups E, V, and W were expressed at low levels or were not expressed in the tissues examined (Figure 5, Table S4). Among the 247 *bZIP* genes, 22 were highly expressed in all tissues, including *BnbZIP13*, *BnbZIP28*, *BnbZIP41*, *BnbZIP2*, *BnbZIP53*, and *BnbZIP60*. Conversely, 42 *BnbZIP* genes were not expressed or were expressed at low levels in all tissues. For example, *BnbZIP14*, *BnbZIP15*, *BnbZIP66*, *BnbZIP3*, *BnbZIP4*, *BnbZIP5*, *BnbZIP*8, *BnbZIP58*, *BnbZIP70*, *BnbZIP75*, and *BnbZIP72* were expressed at low levels in all tissues, and *BnbZIP34c*, *BnbZIP55c*, *BnbZIP30d*, *BnbZIP3f*, *BnbZIP11e*, *BnbZIP48f*, *BnbZIP58b*, *BnbZIP58c*, *BnbZIP75a*, *BnbZIP75b*, and *BnbZIP62c* exhibited little or no expression in any tissue (Figure 5, Table S4). Furthermore, duplicate copies of some gene pairs showed opposite expression patterns in different tissues, such as *BnbZIP55*, *BnbZIP30*, and *BnbZIP11* (Figure 5, Table S4).

**Figure 6:**
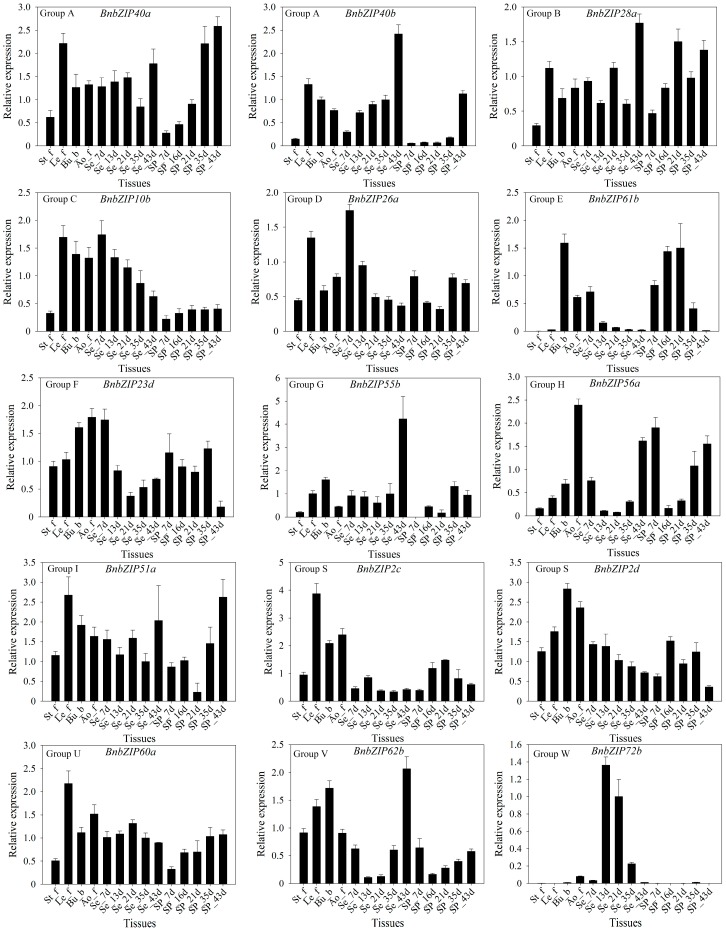
The expression levels of *B*. *napus bZIP* transcription factor family genes at different developmental stages in different tissues and organs, as determined by quantitative real time PCR (qRT-PCR). The subfamily is indicated in the upper-left corner of each graph. The *x* axis corresponds to the different tissues and organs/developmental stages of *B*. *napus* ZS11 (listed in Table S3). *BnACTIN7* (EV116054) and *BnUBC21* (EV086936) were used as the reference gene [41]. Values represent the average ± standard deviation (SD) of three biological replicates with three technical replicates at each developmental stage. Error bars denote SD among three experiments.

We performed qRT-PCR to confirm the expression patterns of differentially expressed genes identified in our transcriptomic analysis. We investigated the expression patterns of 13 *bZIP* genes from different subgroups in 14 tissues and organs of ZS11 (Figure 5 and Figure 6). The expression patterns from RNA-seq and qRT-PCR were highly similar, and highly correlated (*r* = 0.58–0.88; Pearson test, *p* ≤ 0.05), confirming the reproducibility and reliability of the transcriptome data obtained in this study.

## 4. Discussion

### 4.1. Characterization of bZIP Gene Families in Brassica napus

In this study, 247 *BnbZIP* TF family members were predicted to be present in the *B*. *napus* genome database. *bZIP* TF genes have been extensively characterized in other plants, including 75 *bZIP* genes in *Arabidopsis*, 89 in rice, 92 in *Sorghum*, 125 members in maize, and 64 in cucumber [29,30,44]. Compared with these plants, *B*. *napus* contains many more *bZIP* genes, which is not surprising, since *B*. *napus* is an allotetraploid with a complex genome with the larger assembled genome size than that of *B. oleracea* (540 Mb) and *B. rapa* (312 Mb) [25]. In this study, we identified 247 *bZIP* genes in 67 gene families in *B*. *napus*, with two to eight members per family (Table S2). In addition, 75 putative *bZIP* genes were classified into 10 groups in *Arabidopsis* [7]. Based on phylogenetic analysis, all *BnbZIP* members were divided into the same 10 groups as *bZIP* genes from *Arabidopsis* (Figure 1), suggesting similar evolutionary trajectories in *Arabidopsis* and *B*. *napus*. However, Group S was divided into two subgroups (S_1_ and S_2_, Figure 1), and *AtbZIP1*-containing clades were classified into the Group C, indicating that they have not only similar structural features with highly conserved areas, but are consistent with the assumption that the originated as the sister groups before the emergence of land plants [45]. In addition, Groups U, V, and W might represent new groups based on the single group including three *bZIP* members (*AtbZIP60*, *AtbZIP62*, and *AtbZIP72*) in *Arabidopsis* [7]. Like the *bZIP* genes of other plants (cassava, grapevine, apple, and *Brachypodium distachyon*) [44,46], similar gene structures were found in this study, ranging from 0 to 15. Further, 106 of the *BnbZIP* genes have no more than two introns, confirming the hypothesis that the lower intron numbers might be associated with the stress-response [1,28,47,48,49]. However, some *BnbZIP* gene family contains more than 6 introns, such as group C, D, and G (Figure 2, Table S2), which were consistent with the results in rice, indicating that they might contain the original *BnbZIP* genes in these groups [50]. In addition, the motif numbers and composition of were varied in each *bZIP* family, but the motif 1, a basic region of 16 amino acid residues containing an invariant N-x7-R/K motif [7], were widely detected in all bZIP family (Figure 3 and Figure S1), indicating that they have a highly conserved in protein structures. In all, the phylogeny analysis of *BnbZIP* genes is consistent with the gene structures and sharing the similar motifs in each group (Figure 1, Figure 2 and Figure 3), which has also been reported in grape and apple [49,51].

*B*. *napus*, an ideal model for studying the role of polyploidy in the expansion and evolution of gene families, is an allotetraploid species that has undergone widespread genome duplications and merging events [25]. Some other gene superfamilies in the allotetraploid species *B*. *napus* have been described, including the *SnRK2*, *ERF*, *VOC*, *ACBP*, and *MAPKKK* superfamilies [52,53,54,55,56]. In the current study, we found that the copy number of *bZIP* genes varied from two to eight, which is consistent with the finding that two or more copies of homologous genes have been detected in *B*. *napus* [57]. Excessive gene loss is also typical after polyploidization in eukaryotes [39,40]. The complete and accurate annotation of genes is an essential starting point for further evolutionary and functional analyses of gene superfamilies. In this study, 89.88% (222/247) *BnbZIP* genes were accurately mapped onto the 19 *B*. *napus* chromosomes (Figure 4), suggesting that WGD events indeed took place during the evolution of *Brassica* species [58,59]. In addition, while 67 *bZIP* genes were identified in the *Arabidopsis* genome and we identified 136 and 130 bZIP genes in the *B*. *oleracea* and *B*. *rapa* genomes (Table S5), respectively, 247 *BnbZIP* genes were detected in *B*. *napus* (Table S5), implying that more than most of duplicated *bZIP* genes were lost after WGT. Certainly, the similar deletions or losses of genes after WGT have been observed in the nucleotide-binding site (NBS)-encoding genes or Late embryogenesis abundant (LEA) genes of *Brassica* species [48,60]. Furthermore, a few tandemly duplicated genes were detected, most of which were found on chromosomes A04, A05, A08, A09, A10, C03, C04, and C09 (Figure 4). Our results further support the notion that these events may have been the result of chromosomal rearrangement during their evolution of *Brassica* [58]. These results increase our understanding of segmental duplication and WGD for bZIP genes expansion in *B. napus*. 

### 4.2. Expression Profile Analysis Reveals the Potential Roles of *bZIP* Genes in Brassica napus

The bZIP TFs play crucial roles in various biological processes, such as signal transduction, stress responses, and plant growth and development [2]. In *Arabidopsis*, Group A bZIPs, such as AtbZIP39, AtbZIP36, AtbZIP38, AtbZIP66, AtbZIP40, AtbZIP35, and AtbZIP37, are involved in the abiotic stress response or ABA signaling [6,7,24,61]. Numerous bZIP proteins are involved in the drought/osmotic stress response [28,29,30,44], growth and development, organ and tissue differentiation [15], cell elongation [16,17], seed storage protein gene regulation [23], and somatic embryogenesis [24]. In our current study, the 247 bZIP proteins were classified into the same 10 groups as *bZIP* genes from *Arabidopsis* (Figure 1). Our results indicate that the encoding *bZIP* genes, which are homologous to *Arabidopsis* genes, might play similar roles in specific biological processes.

To investigate the functions of these *bZIP* genes in more detail, we investigated their transcriptional patterns, which can provide important clues as to their functions in *B*. *napus*. We constructed heatmap based on the relative expression levels of the *bZIP* genes in 17 tissues at different developmental stages (for a total of 42 samples) in ZS11, including roots, hypocotyls, cotyledons, stems, leaves, buds, flowers, and seeds (Table S3). A strong correlation was found between the results of RNA-seq and qRT-PCR, pointing to the reproducibility and reliability of our results. On the whole, although most *bZIP* genes displayed similar expression patterns among all tissues, these *BnbZIP* genes exhibited significant variations in expression during development, with different copies of the same gene sometimes exhibiting different expression patterns, suggesting that these BnbZIP TFs might be involved in different biological processes in rapeseed. Of the *bZIP* TF genes examined, six (*BnbZIP13*, *BnbZIP28*, *BnbZIP41*, *BnbZIP2*, *BnbZIP53*, and *BnbZIP60*) were highly expressed throughout development, suggesting that they might be regulatory genes that function throughout plant development.

The *bZIP* genes *bZIP28*, *bZIP2*, *bZIP53*, and *bZIP60* play positive roles in abiotic stress signaling and responses [62,63], as well as growth and development [23,64]. However, little is known about the roles of *BnbZIP13* and *BnbZIP41*. Conversely, some *BnbZIP* genes (*BnbZIP14*, *BnbZIP15*, *BnbZIP66*, *BnbZIP3*, *BnbZIP4*, *BnbZIP5*, *BnbZIP*8, *BnbZIP*58, *BnbZIP*70, *BnbZIP*75, and *BnbZIP*72) were expressed at relatively low levels throughout development, and we did not detect the expression of some *BnbZIP* genes, such as *BnbZIP34c*, *BnbZIP55c*, *BnbZIP30d*, *BnbZIP3f*, *BnbZIP11e*, *BnbZIP48f*, *BnbZIP58b*, *BnbZIP58c*, *BnbZIP75a*, *BnbZIP75b*, and *BnbZIP62c*. Furthermore, numerous studies indicate that ABA and stress are likely involved in both the transcriptional and post-translational regulation of several Group A bZIPs [3,7,65]. Therefore, the latter BnbZIP proteins might not be involved in plant differentiation and development in rapeseed under normal growth conditions. Moreover, Group S bZIPs are transcriptionally activated after stress treatment (e.g., cold, drought, anaerobic stress, wounding) or are specifically expressed in the flowers of monocot and dicot plants [66,67,68]. The transcript profiles of the Group S genes *BnbZIP11* and *BnbZIP53* suggest that they might play widespread regulatory roles in the development of *B*. *napus*, as do their homologs in *Arabidopsis* [69,70]. In addition, *BnbZIP28* and *BnbZIP60* were both highly expressed in *B*. *napus*. Interestingly, their homologs in *Arabidopsis* encode endoplasmic reticulum stress sensors [71] that play key roles in UPR signaling [72,73]. The latest results showed that F-*bZIP* genes associated with zinc deficiency and salt stress response in *A. thaliana* [74,75,76] were divided into two subgroups in land plants [77], in accordance the with the present results. In addition, *BnbZIP24* have two members, and lower than *BnbZIP19* and *BnbZIP23*, indicating that *BnbZIP24* have more prone to gene loss and expansion events. And the expression of *BnbZIP24* have lower levels during the whole development stages, but *BnbZIP19* and *BnbZIP23* have higher levels (Figure 5). Thus, the regulatory mechanisms need much more additional studies. Finally, the high transcript levels of many *BnbZIP* genes in specific organs suggest that they primarily function during development. Together, our results suggest that bZIP TFs play crucial roles in protecting plants from all types of biological and abiotic stresses, sometimes under specific conditions, laying the foundation for further analysis of the functions of BnbZIPs in *B*. *napus*.

## 5. Conclusions

In total, 247 putative bZIP transcription factors were identified in *B. napus*, one of the most important oilseed crops and is broadly used for edible vegetable oil and protein-rich meal, and these genes are located in 19 chromosomes, respectively. Further, the 247 bZIP transcription factors were classified in 10 key-clades and three minor-clades based on the phylogenetic analysis. Chromosomal/segmental duplication, and tandem gene duplication, as well as the WGD might have contributed to the major mechanisms contributing to the expansion of the *bZIP* gene superfamily in eukaryotes. In addition, RNA-Seq based analysis qRT-PCR identification revealed tissue specific expression of these bZIPs. six (BnbZIP13, BnbZIP28, BnbZIP41, BnbZIP2, BnbZIP53, and BnbZIP60) were always highly expressed throughout development, whereas others exhibited significant variations in expression during development, with different copies of the same gene sometimes exhibiting different expression patterns, suggesting that these BnbZIP TFs might be involved in different biological processes in rapeseed. Our results suggest that bZIP TFs play crucial roles in protecting plants from all types of biological and abiotic stresses, sometimes under specific conditions. In all, these genes could be utilized to characterize them and laying the foundation for further elucidating their specific response mechanisms and ultimate integration into *B. napus* improvement programs.

## Supplementary Materials

The following are available online at www.mdpi.com/2073-4425/8/10/288/s1. Figure S1: The 247 *B. napus* BnbZIP amino acid sequences were aligned. Table S1: Primers of the *bZIP* genes and housekeeping genes used for qRT-PCR. Table S2: List of the identified *bZIP* genes in *B. napus* genome. Table S3: The tissues and organs from *B. napus* ZS11 used in this study. Table S4: Heat map of expression levels of *B. napus bZIP* transcription factor family genes at different growth periods in different tissues and organs. Table S5: Syntenic gene analysis of *bZIP* genes in *A. thaliana*, *B. rapa*, *B. oleracea* and *B. napus*.
