# Supplementary material for: Genome-Wide Identification and Structural Analysis of bZIP Transcription Factor Genes in Brassica napus
Source: Genes (Basel). 2017 Oct 24;8(10):288. doi: 10.3390/genes8100288 (PMC5664138; doi:10.3390/genes8100288)
Supplement: Supplementary file 1 [file genes-08-00288-s001.zip › Figure S1.pdf]

```
                   10        20        30        40        50        60        70        80        90
BnbZIP12a  -----------------------------------------------------------------------MGSLRGNVEEPISQ-SLPR  18
BnbZIP12b  -----------------------------------------------------------------------MGSLRGNAEEPISQ-SLPR  18
BnbZIP12c  -----------------------------------------------------------------------MGSLRGDMEEPIHQ-SLPR  18
BnbZIP12d  -----------------------------------------------------------------------MGSLRGNVEEPIHQ-SLPR  18
BnbZIP14a  ------------------------------------------------------------------------------------------  1
BnbZIP14b  ------------------------------------------------------------------------------------------  1
BnbZIP14c  ------------------------------------------------------------------------------------------  1
BnbZIP14d  ------------------------------------------------------------------------------------------  1
BnbZIP14e  ------------------------------------------------------------------------------------------  1
BnbZIP14f  ------------------------------------------------------------------------------------------  1
BnbZIP27b  ------------------------------------------------------------------------------------------  1
BnbZIP27a  ------------------------------------------------------------------------------------------  1
BnbZIP38e  -------------------------------------------------MGTHINFNNLGGG-----DPGGEGFNQMKPTDT--AYPLAR  34
BnbZIP38f  -------------------------------------------------MGTHINFNNLGGEG-------------MKPTDN--AYPLAR  26
BnbZIP37a  -------------------------------------------------MGSRMNFVDVVRDEV--------INAKQPALGS--GLPLTR  31
BnbZIP37b  -------------------------------------------------MGSRFNFVD---------------DAKQPALGT--GVPLTR  24
BnbZIP37c  -------------------------------------------------MGSQFNFVD---------------DAKQPALGA--GVPLTR  24
BnbZIP38a  -------------------------------------------------MGTHINFNNLGGGGGGGGDPGGEGFNQMKATDT--AYPLAR  39
BnbZIP38b  -------------------------------------------------MGTHINFNNLGGGGS----------NQMKPTDT--PNPLAR  29
BnbZIP38c  -------------------------------------------------MGTHINFNNIGGGGG------GEGSNQMKPTDN--AYPLAR  33
BnbZIP38d  -------------------------------------------------MGTHINFNSLGGGGS----------YQMKPTDS--PNPLAR  29
BnbZIP39a  -----------------------------------------------MVVRETKMMSGREVESST---RHNGGAGAGGG-ENHPFTSLGR  39
BnbZIP39b  -----------------------------------------------MVGRETEMMSEREVETSTAQARQNGGAGGG---ENHPFSSLGR  40
BnbZIP39c  -----------------------------------------------MVSRETEMMSEREVETSTAQARQNGGAGGGGGGENHPFSSLGR  43
BnbZIP40a  -------------------------------------------------MASFKMMSS--------------STSRNSDLSRRNSSSASS  27
BnbZIP40b  -------------------------------------------------MASFKMMSS--------------STSRNSDLSRRNSSSASS  27
BnbZIP66a  -----------------------------------------------------------------------MDSQR----EPKSH-SLNR  14
BnbZIP66b  -----------------------------------------------------------------------MDSQRVIVEDPKSP-SLNR  18
BnbZIP66c  -----------------------------------------------------------------------MDSQR----EPRSHQSLNR  15
BnbZIP66d  -----------------------------------------------------------------------MDSHRVIVEDPKSQ-FLNR  18
BnbZIP66e  -----------------------------------------------------------------------MDSQR----EPKSH-SLNR  14
BnbZIP66f  -----------------------------------------------------------------------MDSQR----EPRSHQSLNR  15
BnbZIP67a  --------------------------------------MHVHITRFIGLKTMPGPESETSTFHVFNHDLQTQLQTYPQETAVEEHAPVGR  52
BnbZIP67b  --------------------------------------MHVHITRFIGLKTMPGLESETSTFHVFNHDLQTQLQRYPQETAVEEHPPVGR  52
BnbZIP17a  --------------------------------MAESITEEPPPSEFDPL---------FDQLHSSISDQTPIGELMSDLGFPI------D  43
BnbZIP17b  --------------------------------MAESITEEPPPPEFDPIST-----PPFAQFHNPSSDQTPIGELMSDLGFPI------D  47
BnbZIP17c  --------------------------------MSQSITEEPPPPSSDSFS--------IPPFDDQFHHQTPIGELMSDLGFPI------D  44
BnbZIP28a  --------------------------------MTESTFSIANPP---DLNPNTLPDPEMISVPPLDPLFLSASDPISDLSFLLDDENGDL  55
BnbZIP28b  --------------------------------MTESTFSIANPPEMPDLNPNTLPDPEMISVPPLDPLFLSASDPISDLSFFFNDENGDF  58
BnbZIP49a  --------------------------------MAEPAVDS---------------------FYNS---SFELGNSISDL----------D  24
BnbZIP49b  --------------------------------MAEPAVDS---------------------FYNS---SFEIENSISDL----------D  24
BnbZIP9a   -------------------------------------------------------------------------------MDGHTAK----  7
BnbZIP9b   -------------------------------------------------------------------------------MDGHTAK----  7
BnbZIP9c   -------------------------------------------------------------------------------MDGHTAK----  7
BnbZIP9d   -------------------------------------------------------------------------------MDGHTAK----  7
BnbZIP10a  -------------------------------------------------------------------------MNSIFSIDDFSDP--FW  15
BnbZIP10b  -------------------------------------------------------------------------MNSIFSIDDFSDP--FW  15
BnbZIP25a  ------------------------------------------------------------------------------------------  1
BnbZIP25b  -------------------------------------------------------------------------MHIVFSVDDLTDS--FW  15
BnbZIP25c  -------------------------------------------------------------------------MHIVFSVDDLTES--FW  15
BnbZIP25d  ------------------------------------------------------------------------------------------  1
BnbZIP25e  -------------------------------------------------------------------------MKSGFSADDSTDS--FW  15
BnbZIP25f  ------------------------------------------------------------------------------------------  1
BnbZIP63a  -------------------------------------------------------------------------MEKTLSVEEISCN-HLW  16
BnbZIP63b  -------------------------------------------------------------------------MEKTLSVEEISCN-HLW  16
BnbZIP63c  -------------------------------------------------------------------------MEKVFSVEVTSCNNHLW  17
BnbZIP63d  -------------------------------------------------------------------------MEKVFSVEETSCNNHLW  17
BnbZIP20a  ------------------------------------------------------------------------------------------  1
BnbZIP20b  ------------------------------------------------------------------------------------------  1
BnbZIP45d  ------------------------------------------------------------------------------------------  1
BnbZIP45e  ------------------------------------------------------------------------------------------  1
BnbZIP45a  ------------------------------------------------------------------------------------------  1
BnbZIP45b  ------------------------------------------------------------------------------------------  1
BnbZIP45c  ------------------------------------------------------------------------------------------  1
BnbZIP45f  ------------------------------------------------------------------------------------------  1
BnbZIP26a  ------------------------------------------------------------------------------------------  1
BnbZIP26b  ------------------------------------------------------------------------------------------  1
BnbZIP21a  ------------------------------------------------------------------------------------------  1
BnbZIP21b  ------------------------------------------------------------------------------------------  1
BnbZIP21c  ------------------------------------------------------------------------------------------  1
BnbZIP21d  ------------------------------------------------------------------------------------------  1
BnbZIP22a  ------------------------------------------------------------------------------------------  1
BnbZIP22b  ------------------------------------------------------------------------------------------  1
BnbZIP22c  ------------------------------------------------------------------------------------------  1
BnbZIP22d  ------------------------------------------------------------------------------------------  1
BnbZIP50c  ------------------------------------------------------------------------------------------  1
BnbZIP50d  ------------------------------------------------------------------------------------------  1
BnbZIP46a  ------------------------------------------------------------------------------------------  1
BnbZIP46b  ------------------------------------------------------------------------------------------  1
BnbZIP47a  ------------------------------------------------------------------------------------------  1
BnbZIP47b  ------------------------------------------------------------------------------------------  1
BnbZIP47c  ------------------------------------------------------------------------------------------  1
BnbZIP47d  ------------------------------------------------------------------------------------------  1
BnbZIP47e  ------------------------------------------------------------------------------------------  1
BnbZIP50b  ------------------------------------------------------------------------------------------  1
BnbZIP50a  ------------------------------------------------------------------------------------------  1
BnbZIP57a  ------------------------------------------------------------------------------------------  1
BnbZIP57b  ------------------------------------------------------------------------------------------  1
BnbZIP57c  ------------------------------------------------------------------------------------------  1
BnbZIP57d  ------------------------------------------------------------------------------------------  1
BnbZIP65a  ------------------------------------------------------------------------------------------  1
BnbZIP65b  ------------------------------------------------------------------------------------------  1
BnbZIP65c  ------------------------------------------------------------------------------------------  1
BnbZIP34a  ------------------------------------------------------------------------------------------  1
BnbZIP34b  ------------------------------------------------------------------------------------------  1
BnbZIP34c  ------------------------------------------------------------------------------------------  1
BnbZIP34d  ------------------------------------------------------------------------------------------  1
BnbZIP34e  ------------------------------------------------------------------------------------------  1
BnbZIP61a  ------------------------------------------------------------------------------------------  1
BnbZIP61b  ------------------------------------------------------------------------------------------  1
BnbZIP61c  ------------------------------------------------------------------------------------------  1
BnbZIP19a  ------------------------------------------------------------------------------------------  1
BnbZIP19b  ------------------------------------------------------------------------------------------  1
BnbZIP19c  ------------------------------------------------------------------------------------------  1
BnbZIP23b  ------------------------------------------------------------------------------------------  1
BnbZIP23a  ------------------------------------------------------------------------------------------  1
BnbZIP23c  ------------------------------------------------------------------------------------------  1
BnbZIP23d  ------------------------------------------------------------------------------------------  1
BnbZIP24a  ------------------------------------------------------------------------------------------  1
BnbZIP24b  ------------------------------------------------------------------------------------------  1
BnbZIP16a  ----MERNRSRRYVVFAAVRESEEKRFTAVREIEEERFLSFFSLLDSHRIPSFVLRVFRHHPVVRYLSLVIMASNEMEKSSKEKEPTTPP  86
BnbZIP16b  -----------------------------------------------------------------------MASNEMEKSSKEKEPTTPP  19
BnbZIP16c  -----------------------------------------------------------------------MANSEMEKPSKEKELKQPS  19
BnbZIP68a  -----------------------------------------------------------------------MGSSEVDKSGEENEPKTPS  19
BnbZIP68b  -----------------------------------------------------------------------MGSSEVDKSGEENEPKTPP  19
BnbZIP16d  ------------------------------------------------------------------------------------------  1
BnbZIP16e  ------------------------------------------------------------------------------------------  1
BnbZIP41a  -----------------------------------------------------------------------MGTSEENTPFKPSKPASSA  19
BnbZIP41b  -----------------------------------------------------------------------MGTSEEKTPFKPSKPASSA  19
```

BnbZIP66e	QD-SSLYSLTLDEVQTHLD-GSGKALGSMNLDELLKSVCSVDSNG---DT	59
BnbZIP66f	QG--SLYSLTLDEVQTHLG-SSGKALGSMNLDELLKSVCS--	52
BnbZIP67a	QN--SIMSLTLDEIQMK----SGKSFGAMNMDELLANMWMTVEENNGGGAGAQQDGEK--------------------------------	104
BnbZIP67b	QN--SIMSLTLDEIQMK----SGKSFGAMNMDELLANMWMTVEENNGGGAGAQQDGEK--------------------------------	104
BnbZIP17a	ADFELTFDGMEDLYLPAEDETFLLPDNGS-----NQEQFGDFTPESEGSAIFGDTDKNIHR-NSESPKDSDDRCSGDE----RTLDQLS-	122
BnbZIP17b	ADFELTFDGMEDLYLPAEDETFLLPDNAS-----NQEQFGDFTPES-------DDERTLDQ-STPLSSQGSDNCGSDVS---EAVDQKVK	121
BnbZIP17c	GDFELTFDGMEDVYLPAENETFLIP---G-----NQEQFGDFTPESEGSGDCLPKDADKSKSATPLSSQGSGNCGSDVSEGTTVVDQKVK	126
BnbZIP28a	ADLDFSFDDSVDFFDFDLDADLPVAITES-----------IATHADMDSPEIKIVDRGLEDRSDSVHSQVSSQGSK--------------	120
BnbZIP28b	ADLDFSFDDSVDFVDFDLDAEPPVAISEELGSSGDQSPEAIANHANLDSPEEKIVDRGLEDRSDSVHSQVSSQGSKTSGGCDTLSSPENS	148
BnbZIP49a	VDFELTFE---DLYFPSEGESFFIP--------------------------------------VEVEEKATTTCKTSM------------	61
BnbZIP49b	VDFELTFD---DLYFPSENESFFIP--------------------------------------VDVEEEATTTCKTSM------------	61
BnbZIP9a	-----------------DTGMKRSASELALQEYLTKVIPLDPS-------------------FDPMNQDHTGELRDRHLL----------	51
BnbZIP9b	-----------------DTGMKRSASELALQEYLTRVIPLEPS-------------------FDLKNQDHTGELRDRHLL----------	51
BnbZIP9c	-----------------DIGMKKSASELALQELLTK-FPLDPS-------------------FDLMNWDYTCELRDSLLK----------	50
BnbZIP9d	-----------------DIGMKKSASELALQELLTK-FPLDPS-------------------FDLMNWDYTCELRDSLLK----------	50
BnbZIP10a	ESSPPLDS---------DSAKALTAEEWTVEMFFEEIASSVTSAPVGSNNNNNNAIVGVSSAQSLPSVSGQNDFEEDSRFLRR-------	89
BnbZIP10b	ESSPPLDS---------DSAKAVAAEEWTVEMFLEEIASSVTSAPVGNNNNNNNAIVGVSSAQSLPSVSGQNDFEEDSRFLRR-------	89
BnbZIP25a	--------------------MTRSQSEWAFQRLLKENSGSDQT-----NAIDRLSPP----VQSLSTVDETGDVVEIQKPPRN-------	54
BnbZIP25b	PPPPPEPSPGSVPSQTMPDGMTRSQSEWAFQRLLREMSGSDESP----TTINVRSPPPVQSEQSLSTVDETADVVEIQKPPPPPPRNPAD	101
BnbZIP25c	PPPPPEPSPGSVSAQNMPDGMTRSQSEWAFQRLLREMSGSDESP----TTINVRSPPPVQSEQSLSTVDETSDVVEIQKPPPP------D	95
BnbZIP25d	--------------------MVFTAS--THLKFFP---------------INP---------FILSNLVHFSTFILLGS-----------	33
BnbZIP25e	PPPPPSPPLSPG------DGMTRSQSEWAFQMLLEEISSVPAG-----SSISSSSATDNAIGRSYTQVRSESSVSRTEEASSN-------	87
BnbZIP25f	-----------------MDGVVFYCSRLRCCTNLT----------------------------RLLHLDLIPQQAFKRN-----------	34
BnbZIP63a	SELAEETNGTTAK----GVMNRSDSEWAFQRFIQESSSAGEA--------AYGVSVSGPPSPSVPVDSEEYREFLKSKLN----------	84
BnbZIP63b	SELAEETNGTTAK----GMMNRSDSEWAFQRFIQESSSAGEA--------VYGVSVSGPPSPSVPVDSEEYREFLKSKLN----------	84
BnbZIP63c	SEPAKESDGTSG-------MNRSDSEWAFHRFIQESSAGED-----------GVSVSGPSSP---VDSHEYREILKSKLN----------	76
BnbZIP63d	SEPAKESDGTSG-------MNRSDSEWAFHRSIQKSSAGEDAT-------AHGASDSGPSSP---VDSHEYREILKSKLN----------	80
BnbZIP20a	--	1
BnbZIP20b	--	1
BnbZIP45d	--	1
BnbZIP45e	--	1
BnbZIP45a	--	1
BnbZIP45b	--	1
BnbZIP45c	--	1
BnbZIP45f	--	1
BnbZIP26a	--	1
BnbZIP26b	--	1
BnbZIP21a	---MATNHNHSHHLSYSLLHGLSNNPPPPG	27
BnbZIP21b	---MATNHNHSHHLSYSLLHGLSNNPPPPG	27
BnbZIP21c	---MDNHRVISEATNHNDNHHLSYSLLHGLSNNPPPPG	35
BnbZIP21d	--	1
BnbZIP22a	--	1
BnbZIP22b	---MKTRC	5
BnbZIP22c	--	1
BnbZIP22d	--	1
BnbZIP50c	--	1
BnbZIP50d	--	1
BnbZIP46a	---------------------------------------MQSSFKTVPFNPDFYSQASFFFRGDSCLDEFHQPINGFHHDEAVGLSPNVT	51
BnbZIP46b	---------------------------------------MQSSFKTVPFNPDFYSQASFFFRGDSCLDEFHQPINGFHHDEAVGLSPNVT	51
BnbZIP47a	--	1
BnbZIP47b	--	1
BnbZIP47c	--	1
BnbZIP47d	--	1
BnbZIP47e	--	1
BnbZIP50b	--MLHPVSSSLRKK-------------VEALQEF	19
BnbZIP50a	--MLHSVLSSLRKKWSKNTLWKSHTSGLWALQEF	32
BnbZIP57a	--	1
BnbZIP57b	--	1
BnbZIP57c	--	1
BnbZIP57d	--	1
BnbZIP65a	--MQGHHQNHHQQLSSASATSSSGNFMNKDGY	30
BnbZIP65b	--MQGHHQNHHQQLSSASATSSNGNFMNKDGY	30
BnbZIP65c	--MSTD-F	5
BnbZIP34a	------------------------------MAQLPPQIPNM----TPHWPD---------------------------------------	17
BnbZIP34b	------------------------------MAQLPPKIPNM----TPHWPD---------------------------------------	17
BnbZIP34c	------------------------------MAQLPPKIPNM----TPHWPD---------------------------------------	17
BnbZIP34d	------------------------------MAQLPPKIPNM----TPHWPD---------------------------------------	17
BnbZIP34e	------------------------------MAQLPPKIPNM----TPHWPD---------------------------------------	17
BnbZIP61a	------------------------------MAQLPPKIPTMTTTT-PHWPD---------------------------------------	20
BnbZIP61b	------------------------------MAQLPPKIPTMTTTTTPHWPD---------------------------------------	21
BnbZIP61c	------------------------------MAQLPPKIPTMTTTTTPHWPD---------------------------------------	21
BnbZIP19a	--	1
BnbZIP19b	--	1
BnbZIP19c	--	1
BnbZIP23b	--	1
BnbZIP23a	--	1
BnbZIP23c	--	1
BnbZIP23d	--	1
BnbZIP24a	--	1
BnbZIP24b	--	1
BnbZIP16a	---PPP-SSQEPSSAVSAGMATPDWSGFQAY-SPMPPHGFVASSPQPHPYMWGVQHM---------------------------------	138
BnbZIP16b	---PPPPSSQEPSSAVSAGMATPDWSGFQAY-SPMPPHGFVASSPQPHPYMWGVQHMN--------------------------------	73
BnbZIP16c	S-SSAPPPSQEPSSSVSAG---PDWSGFQASSAPMQPHGFVTSSPQPHPYMWRVQHM---------------------------------	72
BnbZIP68a	S-SSAPVTSQEPSSAVAT----PDWSGFQAYSPMPP-HGYVASSPQPHPYMWGVQHM---------------------------------	70
BnbZIP68b	PPSSAPVTSQEPSSAVAT----PDWSGFQAYSPMPPPHGYVASSPQPHPYMWGVQHM---------------------------------	72
BnbZIP16d	--	1
BnbZIP16e	--	1
BnbZIP41a	QDTPP------TPYADWSNSMQAYYGGGGTPSPFFPSPVGSPS---PHPYMWGAQHH---------------------------------	67
BnbZIP41b	QDTPP------TPYADWSNSMQAYYGGGGTPSPFFPSPVGSPS---PHPYMWGAQHH---------------------------------	67
BnbZIP41c	QDIPP------TPYPEWSNSMQAYYGGGGTPNPFFPSPVGSPS---PHPYMWGAQHH---------------------------------	64
BnbZIP41d	QDIPP------TPYPDWSNSMQAYYGGGGTPSPFFPSPVGSPS---PHPYMWGAQHH---------------------------------	64
BnbZIP54a	APPPPPEQSNVHVYHHDWAAMQAYYGPRVAITPQYYNSNGHAAP--PPPYIWGSPSP---------------------------------	74
BnbZIP54b	APPPP-EQSNVHVYHHDWAAMQAYYGPRGAITPQYYNSNGHAP-----PYIWGSPSP---------------------------------	70
BnbZIP55a	PSSPPADQTNVHVYP-DWAAMQAYYGPRVAMPPYYNSAMAAASGHPPPPYMWN-PQH---------------------------------	134
BnbZIP55b	PSSPPADQTNVHVYP-DWAAIQAYYGPRVAMPPYYNSAMAAASGHPPPPYMWN-PQH---------------------------------	110
BnbZIP55c	--MWN-SQH---------------------------------	6
BnbZIP56a	--	1
BnbZIP56b	--	1
BnbZIP56c	--	1
BnbZIP56d	--	1
BnbZIP64a	--	1
BnbZIP64b	--	1
BnbZIP64c	--	1
BnbZIP18a	----------------------------AKSNQCPPPLSN--PTPTPFRGP---------------------------------------	27
BnbZIP18b	----------------------------AKSNQCPPPLSN--PTPTPFRGP---------------------------------------	27
BnbZIP18c	---------------------------TTKPNQCPPPLSTPPPTSTPFRGP---------------------------------------	30
BnbZIP18d	---------------------------TTKSNQCPPPLSTAPPTSTPFRGP---------------------------------------	30
BnbZIP29a	SQPCSFFSLDSLPPLSPSPF-LDDRLNTNPSSLLPP-----FTSSS--------RADSLPPR----------------------------	125
BnbZIP29b	SQPSSFFSFDSLPPLSPSPF-RDH---DHPSSLFTR-----LVG------------ECLPPR----------------------------	117
BnbZIP29c	SQPSSFFSFDSLPPLSPSPF-NNNNNNNPSSSLLPPSPFTRCNSDS--------SRVVLPPR----------------------------	106
BnbZIP29d	SQPSSFFSFDSLPPLSPSPS-NNNNNNNQPSSLLPPPPFTRCNSDS--------SRVVLPPR----------------------------	113
BnbZIP30a	SQPSSFFSFDSLPPLNPSPVSVDERNGAGFSPSLPPSPFTMCHSSSRMVG-GGGDGENLPPR----------------------------	129
BnbZIP30b	SQPSSFFSFDSLPPLNPSPVSAEERNGAGFSPSLPPSPFTMCHSSSRMVGVGGGDGENLPPR----------------------------	142
BnbZIP51a	SHHR----------------RARSDTFFSGESIDDLLLFDPSDVDFSSLDF---------------------------------------	63
BnbZIP51b	SHHR----------------RARSDTFFTGESIDDLLLFDPSDVDFSSLDL---------------------------------------	62
BnbZIP51c	--	1
BnbZIP69d	-------------RYSSFPHLTPPPSGSSDANRFSHDISRMPDNPPKNLG--	53
BnbZIP52a	-----------------------------KSD--PQPPN-ADQIPSSFHR--	20
BnbZIP52b	-----------------------------KPD--PQPPNAADPIPSSFHR--	21
BnbZIP52c	-----------------------------KPD--PPPNPNADPVPSSFHR--	21
BnbZIP52d	-----------------------------KPD--PPPNPNADPVPSSFHR--	21

```
BnbZIP45b  -----------------------------------------------------------------MADTSSRTDASTDGDTD------PR  19
BnbZIP45c  -------------------------------------------------------------MSQLMADTSSRTDVSTDGDTD------PR  23
BnbZIP45f  -----------------------------------------------------------------MADTSSRTDASTDGDTD------PR  19
BnbZIP26a  -----------------------------------------------------------------MGDTSPRTSGSTDGDMD------QN  19
BnbZIP26b  -----------------------------------------------------------------MGDTSPRTSGSTDGDMD------QN  19
BnbZIP21a  FINQDGSSSFDFGELEEAIVLQGVKYRNDETKPPLFAGGGGATTLEMFPSWPIRTHQTLPTEESSKSEGESTDSGSANFSGKAESQQPES  117
BnbZIP21b  FINQDGSSSFDFGELEEAIVLQGVKYRNDETKPPLFAGGGGATTLEMFPSWPIRTHQTLPTEESSKSEGESTDSGSANFSGKAESQQPES  117
BnbZIP21c  FINQDGSSCFDYGELEEAMVLQGVNSSSHEAKPRLFAGGG-ATTLEMFPSWPIRTHKSLPTE-SSKSGGESSDSESANFSGKAESQLPES  123
BnbZIP21d  ------------------MSICRCNDGKFAG---LFAGGG-ATTLEIFPSWPIRTHKSLPTE-SSKSGGESSDSGSANFSGKAESQLPES  67
BnbZIP22a  -----------------------------------MMSSSSSSTTQVVSLREMGMYEP-FQQLS-GWENAFNTIGSSNQNNNNNNNNPSS  53
BnbZIP22b  RNFVIFFYWNILSSECVSTRDSLFISRLRDELSMEMMSSSSSSTTQVVSLREMGMYEP-FQQLS-GWENAFNTIGTSNQNNN---NNPSS  90
BnbZIP22c  ------------------------------------MMSSSSSNTQAVPFRDMGMYEP-FQQLS-GWENTFNTITTNNHNNN----NQTS  48
BnbZIP22d  ------------------------------------MMSSSSSNTQAVPFRDMAMYEP-FQQLT-GWDNTFNTITSNNHNNN----NQTS  48
BnbZIP50c  ------------------------------------MMSSTSP-TQLASLRDMGIYEP-FQQMV-SWGNVFKS-DINDHSPN-----TAS  45
BnbZIP50d  ------------------------------------MMSSSSP-TQLASLRDMGIYEP-FQQMV-SWRNVFKS-DINDHSPN-----TAS  45
BnbZIP46a  VAASNNLHYTTFDTVMDYGMRERLEGEDECLDTGELMYQRGTRLVGGGGGEVNSSLDKWCDSVSAMADNSQHTDTSTDIDTD------DK  135
BnbZIP46b  VAASNNLHYTTFDTVMDYGLRERLEGEDECLDTGELMYQRGTRLVGGGG-EVNSSLDKWCDSVSAMADNSQHTDTSTDIDTD------DK  134
BnbZIP47a  --------------------------------------MN-STSTHFVPPRRVGIYEP-LHQFG-MWGETFKNNIGNGGDMN-----TPS  44
BnbZIP47b  --------------------------------------MN-STSTHFVPPSRVGIYEP-LHQFG-MWGEPFKNNIGNGGSMN-----TPS  44
BnbZIP47c  --------------------------------------MN-STSTHFVPPRRVGIYEP-LHQFG-MWGETFKHNIGNGGDMN-----TPS  44
BnbZIP47d  --------------------------------------MN-STSTHFVPPSRVGIYEP-LHQFG-MWGETFKSNIGNGGSMN-----TPN  44
BnbZIP47e  --------------------------------------MN-STSTHFVPPRRVNIYEP-LHQFG-MWGETFKSNIGNG-NIN-----TPS  43
BnbZIP50b  LVADIIWCVLNRYKRTTRSPFGSVKEALISKQNKEEMMSSFSP-TQLASLRDMGIYEP-FQQLV-SWGNVYKS-DINDHSPN-----TAS  100
BnbZIP50a  LVADIIWCVLHRYKRTIMSPFGSVKKALISKQNKEEMMSSFSP-TQLASLRDMGIYEP-FQQLV-SWGNVYKS-DINDHSPN-----TAS  113
BnbZIP57a  --------------------------------------MN-TTSTHFVPPRRFEIYETTLNQVS-MWEESFKN---NGG-IF-----TPN  41
BnbZIP57b  --------------------------------------MN-TTSTHFVPPRRFEIYETTLNQVS-MWEENFKN---NGG-IY-----IPN  41
BnbZIP57c  --------------------------------------MNTTTSTHFVPPTRFEIYDP-LNQIGTMWEESFKN---NGGGFY-----TPN  43
BnbZIP57d  --------------------------------------MNTTTSSHFVPPTRFEIFDP-LNQISTMWEESFKN---NGGGFY-----TPN  43
BnbZIP65a  DLGEIDPSLFLYLDGQGHHDQPSTAPLPHHHTTQNLAMRPPTSTLNIFPSKPMHIEPPPSSSTPNAVAPSGSTRPSSEPSMD-----LTN  115
BnbZIP65b  DLGEIDPSLFLYLDGQGHHDQPSTAPLPHHHTTQNLAMRPPTSTLNIFPSKPMHIEPPPSSSTPNAVAPSGSTRPSSEPSMD-----LTN  115
BnbZIP65c  DS-------FVY-----------------------------------NVFG--------KTDNTRLVAPSSSTRPSSEPSMD-----LTN  40
BnbZIP34a  -----------------FS-QKLSHFSTAASS---------------AFLDFSACSR--------------GNHRRSISDSIAFLEAPIV  61
BnbZIP34b  -----------------FS-QKLSHFSTVAASS---------------AFLDFSACRR--------------GNHRRSISDSIAFLEAPIV  61
BnbZIP34c  -----------------FSSQNLTATASTAVQNPS---------WVDEFLDFSACRR--------------GNHRRSISDSIAFLEVPFV  67
BnbZIP34d  -----------------FSSQNLTATASTAVQNPS---------WVDEFLDFSACRR--------------GNHRRSISDSIAFLEAPFV  67
BnbZIP34e  -----------------FSSQNLTATASTAVQNPS---------WVDEFLDFSACRR--------------GNHRRSISDSIAFLEVPFV  67
BnbZIP61a  -----------------FSSQKLPSIAATAAATAG----QQNPSWMDEFLDFSTTRR--------------GTHRRSISDSIAFLETP--  73
BnbZIP61b  -----------------FSSQKLPSIAATAAAT---------------FLDFSSTRR--------------GTHRRSISDSIAFLEAP--  63
BnbZIP61c  -----------------FSSQKLPSIAATAAATAATA-GQQNPTWMDEFLDFSSTRR--------------GTHRRSISDSIAFLEAP--  77
BnbZIP19a  --------------MEDGELEFSNQEVFSTSDMNG-LPLTNCSMDTFFDELLMDSN---------AACTHTHTCN-PTGPEN-----AHT  60
BnbZIP19b  --------------MEDGELEFSNQEVFSSSDVVGGLPPSNGSIDSFFDGLLMDSHHQHQHQHQQGACTHTHTCNNSTGTADN----THS  72
BnbZIP19c  --------------MEDGELE-----VFSS----------NGSIDSFFDGLLMDSH----HQHQQGACTHTHTCNNSTGTEP------HA  51
BnbZIP23b  --------------MDDGELEFP---------------PSSSSMDSFLEELLKDSH----------ACTHTHTCN-PPGPEN-----THT  45
BnbZIP23a  --------------MEDGELEFP---------------PSSSSMDSFLEELLKDSH----------ACTHTHTCN-PPGPEN-----THT  45
BnbZIP23c  --------------MDDGEVEFP---------------LSSSSMDSFFDELLRDSH----------ACTHTHTCN-PPGPEN-----THT  45
BnbZIP23d  --------------MDDGEVEFP---------------LSSSSMDSFFDELLRDSH----------ACTHTHTCN-PPGPEN-----THT  45
BnbZIP24a  --------------MDGEEVE----------------------------AWARASG----------GCSHTHSCN-PPGPEDA----SHS  33
BnbZIP24b  --------------MDGEEIE----------------------------PWARASG----------GCSHTHSCN-PPGPEDA----SHS  33
BnbZIP16a  --------IPPYGTPPHPYVAMYPPGG-MYGHPSMPPGSYPYSPYAMPSPNGMTEASGNTTGGTEGGDAKQSEVKEKLPIKRSRG-SLGS  218
BnbZIP16b  -------MMPPYGTPPHPYVAMYPPGG-MYGHPSMPPGSYPYSPYAMPSPNGMTEASGNTTGGTEGGDAKQSEVKEKLPIKRSRG-SLGS  154
BnbZIP16c  --------MPPYGTPPHPYVTMYPPGG-MYAHPSMPPGSYPYSP--MPSPNGVTEASGNTTGG-TEGDSKRSDVKEKLPIRRSKR----I  146
BnbZIP68a  --------MPPYGTPPHPYVAMYPPGG-MYAHPSIPPGSYPYSPYAMPSPNGMAEASGNTGSG-VEGDGKQAEVKEKLPIKRSKG-SLGS  149
BnbZIP68b  --------MPPYGTPPHPYVAMYPPGG-LYAHPSLPPGSYPYSPYTMPSPNGMAEASGNTGSG-VEGDGKQAEAKEKLPIKRSKG-SLGS  151
BnbZIP16d  --------MPPYGTPTHPYVTMYPPGG-MYGHPSIPPGSYPYSPYAMPSPNGMTEASGNTTGG-TEVEAKQSEMKEKLPIKRSRGGSLGS  80
BnbZIP16e  -----------------------------MELTLKVG-----------RVLLMVQVK-----------EVMQTLKIT-----------LD  28
BnbZIP41a  -------MMPPYGTP-VPYPAMYPPGT-VYAHPGMPMP-------------QASGPTNTEIVKAQAPGKKPKGNSKRKSGGGEKVLSGSG  135
BnbZIP41b  -------MMPPYGTP-VPYPAMYPPGT-VYAHPGMPMP-------------QASGPTNTETVKAQAPGKKPKGNLKRKSGGSEKAPSGSG  135
BnbZIP41c  -------MMPPYGTP-VPYPAMYPPGA-VYAHPGMPMP-------------PASAPTNKETVKEQAPGKKSKGSLKRKGEGGEKAPSGSG  132
BnbZIP41d  -------MMPPYGTP-VPYPAMYPPGA-VYAHPGMPMP-------------PSSAPTN-ETVKEQAPGKKSKGSLKRKGEGGEKAPSGSG  131
BnbZIP54a  -------MMAPYGT---PYPPFCPPGG-VYAHPALQMGSQPQGPASQA-TPVVATPLNLEAHPANSSGNTDQGFMTKLKEFDGLAMSISN  152
BnbZIP54b  -------MMAPYGT---PYPPFCPPGG-VYAHPALQMGTQPQGAVSQA-TPVVATPLNLEAHPANSSGNTDQGFMKKLKEFDGLAMSISN  148
BnbZIP55a  -------MMSPYGT---PYAAVYPHGGGVYAHPGFPMGSQPQGQKGATPLTTPGTPLNIDT-PSKSTGNTENGLMKKLKEFDGLAMSLGN  213
BnbZIP55b  -------MMSPYGT---PYAAVYPHGGGVYAHPGFPM---PQSQKGAA-LSTPGTPLNIDT-PSKSTGNTENGLMKKLKEFDGLAMSLGN  185
BnbZIP55c  -------MMSPYGT---PYAAVYPHGGGVSAHPGFPMRSQPQVQKGPP-LTTPGTPLNMDT-PTKSTGNTDNGLMKKLKEFDGLAMSLG-  83
BnbZIP56a  ---------------------------------------------------------------MQEQTTSSLPASSLPSSSERSSSSAPH  27
BnbZIP56b  ---------------------------------------------------------------MQEQTTSSLPASSLPSSSERSSSSAPH  27
BnbZIP56c  ---------------------------------------------------------------MQEQATSSLAASSLPSSSERSSSSNPH  27
BnbZIP56d  ---------------------------------------------------------------MQEQATS-----SLPSSSERSSSSNPH  22
BnbZIP64a  --------------------------------------------------------------------------MSLQRPNGNSSSS-SS  15
BnbZIP64b  --------------------------------------------------------------------------MSLQRPNGNSSSS-SS  15
BnbZIP64c  ---------------------------------------------MVRYSKVTSYSLYSWSSSKSQRQIKKNILMSLQRPNGNSSSSSPS  45
BnbZIP18a  ------------------YHRRAHSEVQFRLPED---------------LDLSEPF----------------GGFDDLGSEDDIFCTYMD  68
BnbZIP18b  ------------------YHRRAHSEVQFRLPED---------------LDLSEPF----------------GGFDDLGSEDDIFCTYMD  68
BnbZIP18c  ------------------YHRRAHSEVQFRLPED---------------LDLSEPF----------------GGFEDLGSEDDLFCSYMD  71
BnbZIP18d  ------------------YHRRAHSEVQFRLPED---------------LDLSEPF----------------GGFEDLGSEDDLFCSYMD  71
BnbZIP29a  -----------------KSHRRSNSDIPTGLT----------------SNPFVKK-----------------------EDMDDLFSAYMN  159
BnbZIP29b  -----------------KSHRRSNSDIPTGLSP------IPPTPLERSTNPFVKK-----------------------EDMDDLFSAYMN  161
BnbZIP29c  -----------------KSHRRSNSDISG---------GESADWSTKTTPPFVKKESGER------------------EDMDDLFSAYMN  152
BnbZIP29d  -----------------KSHRRSNSDIPTSIPPRPLERGESPDWSN-HTPPFVKKESGE--------------------DMDDLFSAYMN  165
BnbZIP30a  -----------------KSHRRSNSDVTFGFSSV------MSPPLIPSRSLERSVS-------------GGGEASSDCEAMDDVFRAYMN  183
BnbZIP30b  -----------------KSHRRSNSDVTFGFSSI------MSPPLIPSRSLERSEEPG----------EGGGRKKSESEAMDDVFRAYMN  199
BnbZIP51a  ------------------LNAPTPQTQPQPQPQP---------------MSVDSPP----------------EETSSNGAPPIPLPPGRH  104
BnbZIP51b  ------------------LNAPA------PQPSL---------------MYVDSPP----------------EETSSNGAPQIHLPPGRH  97
BnbZIP51c  -------------------------------------------------MSVDSPP----------------EETSSNGAPQIPLPPGRH  25
BnbZIP69d  -------------------HRRAHS-EILTLPDD---------------LSFDSDLGVVGG-------AADGPSFSDDADE-DLMSMYLD  100
BnbZIP52a  -------------------RSRSDDMSMFMFMDP---------------LSSSGPP----------------PSSDDLPSDDDLFSSFID  60
BnbZIP52b  -------------------RSRSDDMSMFMFMDP---------------LSSSGPP----------------PSSDDLPSDDDLFSSFID  61
BnbZIP52c  -------------------RTRSDDMSMFMFTDP---------------LSSAAAP----------------PSSDDLPSDDDLFSSFID  61
BnbZIP52d  -------------------RSRSDDMSMFMFTDP---------------LSS-AAP----------------PSSDDLPSDDDLFSSFID  60
BnbZIP59a  -------------------HRRAHS-EILTLPDD---------------LSFDSDLGVVGTN------AADGASFSDDTEE-ELLSMYLD  85
BnbZIP59b  -------------------HRRAHS-EILTLPDD---------------LSFDSDLGVVGTN------AADGASFSDDTEE-DLLSMYLD  94
BnbZIP59c  -------------------HRRAHS-EILTLPDD---------------LSFDFDLGVVGG-------SADVPSFSDDTEGGDLMSMYLD  97
BnbZIP59d  -------------------HRRAHS-EILTLPDD---------------LSFDFDLGVVGG-------SADVPSFSDDTEGGDLMSMYLD  99
BnbZIP69a  -------------------HRRAHS-EILTLPDD---------------LSFDSDLGVVGA-------AADGPSFSDDTDE-DLLSMYLD  117
BnbZIP69b  -------------------HRRAHS-EILTLPDD---------------LSFDSDLGVVGG-------APDGPSFSDDADE-DLMSMYLD  104
BnbZIP69c  -------------------HRRAHS-EILTLPDD---------------LSFDSDLGVVGA-------AADGPSFSDDTDE-DLLSMYLD  110
BnbZIP1a   ------------------------------------------------------------------------------------------  1
BnbZIP1b   ------------------------------------------------------------------------------------------  1
BnbZIP1c   ------------------------------------------------------------------------------------------  1
BnbZIP1d   ------------------------------------------------------------------------------------------  1
BnbZIP2a   ------------------------------------------------------------------------------------------  1
BnbZIP2b   ------------------------------------------------------------------------------------------  1
BnbZIP2c   ------------------------------------------------------------------------------------------  1
BnbZIP2d   ------------------------------------------------------------------------------------------  1
BnbZIP3a   -----------------------------------------------MQPNYDSSS---LNSMQ---QQDYFHLNHYYNNLNPSTNINNL  37
BnbZIP3b   -----------------------------------------------MQPNYDSSS---FNNMQ---QQDYFHLNHYYNNLNPSTNINNL  37
BnbZIP3c   -----------------------------------------------MQPNYDSSS---LNNMQ---QQDYFHLNHYYNNLNPSNN--NL  35
BnbZIP3d   -----------------------------------------------MHPNYDSSS---INNMQ---QQDYFHLNPYYNNLNPSTN-NNL  36
BnbZIP3e   -----------------------------------------------MQPHYDSSS---LNNMQ---QQDYFHLNHYYNNLNPSNN--NL  35
BnbZIP3f   -----------------------------------------------MHPNYDSSS---INNMQ---QQDYFHLNPYYNNLNPSTN-NNL  36
BnbZIP3g   -----------------------------------------------MHPNYDSSS---INNMQ---QQDYFHLNPYYNNLNPSTN-NNL  36
BnbZIP4a   ---------------------------------------------------------MSSTSLEP-------------------VRICFD  14
BnbZIP5a   --------------------------------------------------MMSTIHPVLSCEPGQTTHVPAFETGFTPWDNS--HLFSVF  38
BnbZIP7a   --------------------------------------------------MLSTVPAISFLEPGTVNHFSGFQTGFTPWEGDCFDLLSMN  40
BnbZIP7b   --------------------------------------------------MLSTVPAFSMSDPGLVNQFQGFQTGFTPWEWDCSDLFSVN  40
BnbZIP7c   --------------------------------------------------MLSTVPAISFLEPGTVNHFSGFQTGFTPWEGDCFDLLSMN  40
BnbZIP7d   --------------------------------------------------MLSTVPAFSMSEPGLVNQFQGFQTGFTPWEWDCSDLFSVN  40
BnbZIP11a  ------------------------------------------------------------------------------------------  1
BnbZIP11b  ------------------------------------------------------------------------------------------  1
BnbZIP11c  ------------------------------------------------------------------------------------------  1
BnbZIP11d  ------------------------------------------------------------------------------------------  1
```

BnbZIP34b	TSNMEEHN---	69
BnbZIP34c	RGHGSTQN-NH--	77
BnbZIP34d	RGHGSNQNDHH--	78
BnbZIP34e	RGHGSTQN-NH--	77
BnbZIP61a	SSGVGSHH---	81
BnbZIP61b	SSGVGNHH---	71
BnbZIP61c	SSGVGNHH---	85
BnbZIP19a	HTCFHVHTKILP---	72
BnbZIP19b	HTCFHVHTKVLP---	84
BnbZIP19c	HTCFHVHTKILP---	63
BnbZIP23b	HTCLHVHTKILPA--	58
BnbZIP23a	HTCLHVHTKILPA--	58
BnbZIP23c	HTCLHVHTKILP---	57
BnbZIP23d	HTCLHVHTKILP---	57
BnbZIP24a	HTCFHTHTHLII---	45
BnbZIP24b	HTCFHTHTHLII---	45
BnbZIP16a	LNMITGKNNEP--GKNSGASA	237
BnbZIP16b	LNMITGKNNEP--GKNSGASA	173
BnbZIP16c	LSMMKGKNNEP--GKNSGASA	165
BnbZIP68a	LNMIIGKKNEA--GKNSGASA	168
BnbZIP68b	LNMIIGKKNEA--GKNSGASA	170
BnbZIP16d	LNMITGKN--SGASA	93
BnbZIP16e	LDNTTG---	34
BnbZIP41a	NDAVSQ--SEESV	146
BnbZIP41b	NDAVSQ--SEESV	146
BnbZIP41c	NDGVSH--SDESV	143
BnbZIP41d	NDGVSH--SDESV	142
BnbZIP54a	NKSGSGEHSSE--PKNSQSSE	171
BnbZIP54b	NKTGSAEHSSE--PKNSQSSE	167
BnbZIP55a	GNNGDEGKRS--RNSSE	228
BnbZIP55b	GNNGDEGKRS--RNSSE	200
BnbZIP55c	-TAADERKRS--WNSSE	97
BnbZIP56a	LEI--	30
BnbZIP56b	LEI--	30
BnbZIP56c	LEI--	30
BnbZIP56d	LEI--	25
BnbZIP64a	PKK--	18
BnbZIP64b	PKK--	18
BnbZIP64c	PKK--	48
BnbZIP18a	IEKLGG---	74
BnbZIP18b	VEKLG--	73
BnbZIP18c	IEKLG--	76
BnbZIP18d	IEKLG--	76
BnbZIP29a	LDNIDALNSSE--	170
BnbZIP29b	LDNIDAS---D--	169
BnbZIP29c	LENIDAL--	159
BnbZIP29d	LENIDAL--	172
BnbZIP30a	LDNIDVL--	190
BnbZIP30b	LDNIDVL--	206
BnbZIP51a	VRSFSVD--	111
BnbZIP51b	VRSFSVD--	104
BnbZIP51c	VRSFSVD--	32
BnbZIP69d	MDKFNSS--	107
BnbZIP52a	VDSLS--	65
BnbZIP52b	VDSLS--	66
BnbZIP52c	VDSLS--	66
BnbZIP52d	VDSLS--	65
BnbZIP59a	MDKFNSS--	92
BnbZIP59b	MDKFNSS--	101
BnbZIP59c	MDKFNSS--	104
BnbZIP59d	MDKFNSS--	106
BnbZIP69a	MDKFNSS--	124
BnbZIP69b	MDKFNSS--	111
BnbZIP69c	MDKFNSS--	117
BnbZIP1a	---	1
BnbZIP1b	---	1
BnbZIP1c	---	1
BnbZIP1d	---	1
BnbZIP2a	---	1
BnbZIP2b	---	1
BnbZIP2c	---	1
BnbZIP2d	---	1
BnbZIP3a	NLIPYPQIH--	46
BnbZIP3b	NLIPYPQIH--	46
BnbZIP3c	NIFPYPQFQ--	44
BnbZIP3d	NLLSYPQIQ--	45
BnbZIP3e	NLIPYPQFQ--	44
BnbZIP3f	NLISYPQMQ--	45
BnbZIP3g	NLLSYPQIQ--	45
BnbZIP4a	DATITGDEIT---	24
BnbZIP5a	DSS-MNIIPH---	47
BnbZIP7a	HFFIEPAVPS---	50
BnbZIP7b	QLSLEPAVPS---	50
BnbZIP7c	QFLIEPVVPS---	50
BnbZIP7d	QLSLEPAVPS---	50
BnbZIP11a	---	1
BnbZIP11b	---	1
BnbZIP11c	---	1
BnbZIP11d	---	1
BnbZIP11e	---	1
BnbZIP42a	NPNSFYGFQN---	47
BnbZIP42b	NPNSFYGFQN---	47
BnbZIP42c	NLNPFYGIQS---	47
BnbZIP44a	-------MNK---	3
BnbZIP44b	-------MNK---	3
BnbZIP44c	-------MNK---	3
BnbZIP44d	-------MNK---	3
BnbZIP48a	INNNN--YSS---	54
BnbZIP48b	INNNN--YSS---	54
BnbZIP48c	INNN---YSS---	53
BnbZIP48d	INNN---YSS---	53
BnbZIP48e	IINNN--YSL---	54
BnbZIP48f	IINNN--YSL---	54
BnbZIP53a	---	1
BnbZIP53b	---	1
BnbZIP53c	---	1
BnbZIP53d	---	1
BnbZIP58a	VTKNNNNYSS---	60
BnbZIP58b	MTNNNNNYSS---	60
BnbZIP58c	MTNNNNNYSS---	60
BnbZIP58d	FTKKKKKIKY---	32
BnbZIP60a	DDP----TADS--VTGKVD	93
BnbZIP60b	DDP----TSDSADL---VTGKVD	95
BnbZIP62a	KAADLAYENEN--LRREKDWA	193
BnbZIP62b	KAADLAYENEN--LRREKDWA	190
BnbZIP62c	NAADLTYENEN--LRR-----	43
BnbZIP36a	VREEAQ--IAAKDAN	135
BnbZIP36b	VREEAQ--IAAKDAN	174
BnbZIP13a	EDGGG--	140
BnbZIP13b	EDGGG--	120
BnbZIP8a	PLVN---	26

```
BnbZIP56a - - - - - - - - - - - - - - - - - - - - - - - - - - - - - - - - - - - - - - - - - - - - - - - - - - - - - - - - - - - - - - - - - - - - - - - - - - K E G I E S D E E - - - - - 39
BnbZIP56b - - - - - - - - - - - - - - - - - - - - - - - - - - - - - - - - - - - - - - - - - - - - - - - - - - - - - - - - - - - - - - - - - - - - - - - - - - K E G I E S D E E - - - - - 39
BnbZIP56c - - - - - - - - - - - - - - - - - - - - - - - - - - - - - - - - - - - - - - - - - - - - - - - - - - - - - - - - - - - - - - - - - - - - - - - - - - K E G I E S D E E - - - - - 39
BnbZIP56d - - - - - - - - - - - - - - - - - - - - - - - - - - - - - - - - - - - - - - - - - - - - - - - - - - - - - - - - - - - - - - - - - - - - - - - - - - K E G I E S D E E - - - - - 34
BnbZIP64a - - - - - - - - - - - - - - - - - - - - - - - - - - - - - - - - - - - - - - - - - - - - - - - - - - - - - - - - - - - - - - - - - - - - - - - - - - R T T E E S D E E - - - - - 27
BnbZIP64b - - - - - - - - - - - - - - - - - - - - - - - - - - - - - - - - - - - - - - - - - - - - - - - - - - - - - - - - - - - - - - - - - - - - - - - - - - H K T E E S D E E E - - - - 28
BnbZIP64c - - - - - - - - - - - - - - - - - - - - - - - - - - - - - - - - - - - - - - - - - - - - - - - - - - - - - - - - - - - - - - - - - - - - - - - - - - Q K T E E S E E E - - - - - 57
BnbZIP18a - - - - - - - - - - - - - - - - - - - - - - - - - - - - - - - - - - - - - - - - - - - - - - - - - - - - - - - - - - - - S G S D S A A - - - P K S D N P L F A G - E E G G S S S 98
BnbZIP18b - - - - - - - - - - - - - - - - - - - - - - - - - - - - - - - - - - - - - - - - - - - - - - - - - - - - - - - - - - - - S G S D S A G - - - P R S D N P L F S G - E E G G S S S 97
BnbZIP18c - - - - - - - - - - - - - - - - - - - - - - - - - - - - - - - - - - - - - - - - - - - - - - - - - - - - - - - - - - - - S G S D S A G P S A P R S D N P F P - - - - E A G D S - 99
BnbZIP18d - - - - - - - - - - - - - - - - - - - - - - - - - - - - - - - - - - - - - - - - - - - - - - - - - - - - - - - - - - - - S G S D S A G P S A P R S D N P F S - - - - E A G D S - 99
BnbZIP29a - - - - - - - - - - - - - - - - - - - - - - - - - - - - - - - - - - - - - - - - - - - - - - - - - - - - - - - - - - - - N L S R D D M E S S R A S G T K T N - - S D T E G E T S 196
BnbZIP29b - - - - - - - - - - - - - - - - - - - - - - - - - - - - - - - - - - - - - - - - - - - - - - - - - - - - - - - - - - - - N L S R D D M E S S R A S G T K T N - - S D T E G D S S 195
BnbZIP29c - - - - - - - - - - - - - - - - - - - - - - - - - - - - - - - - - - - - - - - - - - - - - - - - - - - - - - - - - - - - N S P E A D - - - - R A S G T K T N G S D D T E G E S S 183
BnbZIP29d - - - - - - - - - - - - - - - - - - - - - - - - - - - - - - - - - - - - - - - - - - - - - - - - - - - - - - - - - - - - N S S E A D M E S S R A S G T K T N G S D D T E G E S S 200
BnbZIP30a - - - - - - - - - - - - - - - - - - - - - - - - - - - - - - - - - - - - - - - - - - - - - - - - - - - - - - - - - - - - - N S F G G N E T V D S S G T R K S S S D S E G E S S - 216
BnbZIP30b - - - - - - - - - - - - - - - - - - - - - - - - - - - - - - - - - - - - - - - - - - - - - - - - - - - - - - - - - - - - - N S F G G N E T V D S S G - - - - - - - S E G E S S - 225
BnbZIP51a - - - - - - - - - - - - - - - - - - - - - - - - - - - - - - - - - - - - - - - - - - - - - - - - - - - - - - - - - - - - S E S N F F D D L T A T E D T Q F A R P T R S G G R K G 139
BnbZIP51b - - - - - - - - - - - - - - - - - - - - - - - - - - - - - - - - - - - - - - - - - - - - - - - - - - - - - - - - - - - - S D S D F F D D L T A T E D N Q F A R A T S S G E R K G 132
BnbZIP51c - - - - - - - - - - - - - - - - - - - - - - - - - - - - - - - - - - - - - - - - - - - - - - - - - - - - - - - - - - - - S D S D F F D D V T A T E D K Q F A R A T S S G E R K G 60
BnbZIP69d - - - - - - - - - - - - - - - - - - - - - - - - - - - - - - - - - - - - - - - - - - - - - - - - - - - - - - - - - - - - A S S S - - - - - - C E P A E L V W R N E L G S T T T P 129
BnbZIP52a - - - - - - - - - - - - - - - - - - - - - - - - - - - - - - - - - - - - - - - - - - - - - - - - - - - - - - - - - - - - S N P H P T T T A F P N H A A A A A A A S N S V P S S S 93
BnbZIP52b - - - - - - - - - - - - - - - - - - - - - - - - - - - - - - - - - - - - - - - - - - - - - - - - - - - - - - - - - - - - S N P H P T T I A F P N H H A A A - - - - N S V P S S S 90
BnbZIP52c - - - - - - - - - - - - - - - - - - - - - - - - - - - - - - - - - - - - - - - - - - - - - - - - - - - - - - - - - - - - S N P N P P - I S F P D P S S A A V P - - - - P P N S S 89
BnbZIP52d - - - - - - - - - - - - - - - - - - - - - - - - - - - - - - - - - - - - - - - - - - - - - - - - - - - - - - - - - - - - S N P D P P - I S F P D P S - P A V P - - - - P - S S S 86
BnbZIP59a - - - - - - - - - - - - - - - - - - - - - - - - - - - - - - - - - - - - - - - - - - - - - - - - - - - - - - - - - - - - T A T S S A A A Q V G E P S - - - W R N E S V G M H T - 116
BnbZIP59b - - - - - - - - - - - - - - - - - - - - - - - - - - - - - - - - - - - - - - - - - - - - - - - - - - - - - - - - - - - - T A T S S A A A Q V G E P S - - - W R N E S V G M H T - 125
BnbZIP59c - - - - - - - - - - - - - - - - - - - - - - - - - - - - - - - - - - - - - - - - - - - - - - - - - - - - - - - - - - - - T A T S S - - A Q V G E P S G T A W K N E P M M P Q N - 129
BnbZIP59d - - - - - - - - - - - - - - - - - - - - - - - - - - - - - - - - - - - - - - - - - - - - - - - - - - - - - - - - - - - - T A T S S - - A Q V G E P S R T A W K N E S V M P Q N - 131
BnbZIP69a - - - - - - - - - - - - - - - - - - - - - - - - - - - - - - - - - - - - - - - - - - - - - - - - - - - - - - - - - - - - E A S S S - - - L V G E P S E P S W R N E L G S T - - - 146
BnbZIP69b - - - - - - - - - - - - - - - - - - - - - - - - - - - - - - - - - - - - - - - - - - - - - - - - - - - - - - - - - - - - A S S S - - - - - - C E P A E L V W R N E L A S T T T P 133
BnbZIP69c - - - - - - - - - - - - - - - - - - - - - - - - - - - - - - - - - - - - - - - - - - - - - - - - - - - - - - - - - - - - E A S S S - - - Q M C E P S E P A W N - - - - - - - - - 133
BnbZIP1a - - - - - - - - - - - - - - - - - - - - - - - - - - - - - - - - - - - - - - - - - - - - - - - - - - - - - - - - - - - - - - - - - - - - - - - - - - - - - - - - - - - - - - - - - - - - 1
BnbZIP1b - - - - - - - - - - - - - - - - - - - - - - - - - - - - - - - - - - - - - - - - - - - - - - - - - - - - - - - - - - - - - - - - - - - - - - - - - - - - - - - - - - - - - - - - - - - - 1
BnbZIP1c - - - - - - - - - - - - - - - - - - - - - - - - - - - - - - - - - - - - - - - - - - - - - - - - - - - - - - - - - - - - - - - - - - - - - - - - - - - - - - - - - - - - - - - - - - - - 1
BnbZIP1d - - - - - - - - - - - - - - - - - - - - - - - - - - - - - - - - - - - - - - - - - - - - - - - - - - - - - - - - - - - - - - - - - - - - - - - - - - - - - - - - - - - - - - - - - - - - 1
BnbZIP2a - - - - - - - - - - - - - - - - - - - - - - - - - - - - - - - - - - - - - - - - - - - - - - - - - - - - - - - - - - - - - - - - - - - - - - - - - - - - - - - - - - - - - - - - - - - - 1
BnbZIP2b - - - - - - - - - - - - - - - - - - - - - - - - - - - - - - - - - - - - - - - - - - - - - - - - - - - - - - - - - - - - - - - - - - - - - - - - - - - - - - - - - - - - - - - - - - - - 1
BnbZIP2c - - - - - - - - - - - - - - - - - - - - - - - - - - - - - - - - - - - - - - - - - - - - - - - - - - - - - - - - - - - - - - - - - - - - - - - - - - - - - - - - - - - - - - - - - - - - 1
BnbZIP2d - - - - - - - - - - - - - - - - - - - - - - - - - - - - - - - - - - - - - - - - - - - - - - - - - - - - - - - - - - - - - - - - - - - - - - - - - - - - - - - - - - - - - - - - - - - - 1
BnbZIP3a - - - - - - - - - - - - - - - - - - - - - - - - - - - - - - - - - - - - - - - - - - - - - - - - - - - - - - - - - - - - - - - - - - - - - - - - - - - - - - - - - - - - - - - - - - - - 46
BnbZIP3b - - - - - - - - - - - - - - - - - - - - - - - - - - - - - - - - - - - - - - - - - - - - - - - - - - - - - - - - - - - - - - - - - - - - - - - - - - - - - - - - - - - - - - - - - - - - 46
BnbZIP3c - - - - - - - - - - - - - - - - - - - - - - - - - - - - - - - - - - - - - - - - - - - - - - - - - - - - - - - - - - - - - - - - - - - - - - - - - - - - - - - - - - - - - - - - - - - - 44
BnbZIP3d - - - - - - - - - - - - - - - - - - - - - - - - - - - - - - - - - - - - - - - - - - - - - - - - - - - - - - - - - - - - - - - - - - - - - - - - - - - - - - - - - - - - - - - - - - - - 45
BnbZIP3e - - - - - - - - - - - - - - - - - - - - - - - - - - - - - - - - - - - - - - - - - - - - - - - - - - - - - - - - - - - - - - - - - - - - - - - - - - - - - - - - - - - - - - - - - - - - 44
BnbZIP3f - - - - - - - - - - - - - - - - - - - - - - - - - - - - - - - - - - - - - - - - - - - - - - - - - - - - - - - - - - - - - - - - - - - - - - - - - - - - - - - - - - - - - - - - - - - - 45
BnbZIP3g - - - - - - - - - - - - - - - - - - - - - - - - - - - - - - - - - - - - - - - - - - - - - - - - - - - - - - - - - - - - - - - - - - - - - - - - - - - - - - - - - - - - - - - - - - - - 45
BnbZIP4a - - - - - - - - - - - - - - - - - - - - - - - - - - - - - - - - - - - - - - - - - - - - - - - - - - - - - - - - - - - - - - - - - - - - - - - - - - - - - - - - - - - - - - - - - - - - 24
BnbZIP5a - - - - - - - - - - - - - - - - - - - - - - - - - - - - - - - - - - - - - - - - - - - - - - - - - - - - - - - - - - - - - - - - - - - - - - - - - - - - - - - - - - - - - - - - - - - - 47
BnbZIP7a - - - - - - - - - - - - - - - - - - - - - - - - - - - - - - - - - - - - - - - - - - - - - - - - - - - - - - - - - - - - - - - - - - - - - - - - - - - - - - - - - - - - - - - - - - - - 50
BnbZIP7b - - - - - - - - - - - - - - - - - - - - - - - - - - - - - - - - - - - - - - - - - - - - - - - - - - - - - - - - - - - - - - - - - - - - - - - - - - - - - - - - - - - - - - - - - - - - 50
BnbZIP7c - - - - - - - - - - - - - - - - - - - - - - - - - - - - - - - - - - - - - - - - - - - - - - - - - - - - - - - - - - - - - - - - - - - - - - - - - - - - - - - - - - - - - - - - - - - - 50
BnbZIP7d - - - - - - - - - - - - - - - - - - - - - - - - - - - - - - - - - - - - - - - - - - - - - - - - - - - - - - - - - - - - - - - - - - - - - - - - - - - - - - - - - - - - - - - - - - - - 50
BnbZIP11a - - - - - - - - - - - - - - - - - - - - - - - - - - - - - - - - - - - - - - - - - - - - - - - - - - - - - - - - - - - - - - - - - - - - - - - - - - - - - - - - - - - - - - - - - - - - 1
BnbZIP11b - - - - - - - - - - - - - - - - - - - - - - - - - - - - - - - - - - - - - - - - - - - - - - - - - - - - - - - - - - - - - - - - - - - - - - - - - - - - - - - - - - - - - - - - - - - - 1
BnbZIP11c - - - - - - - - - - - - - - - - - - - - - - - - - - - - - - - - - - - - - - - - - - - - - - - - - - - - - - - - - - - - - - - - - - - - - - - - - - - - - - - - - - - - - - - - - - - - 1
BnbZIP11d - - - - - - - - - - - - - - - - - - - - - - - - - - - - - - - - - - - - - - - - - - - - - - - - - - - - - - - - - - - - - - - - - - - - - - - - - - - - - - - - - - - - - - - - - - - - 1
BnbZIP11e - - - - - - - - - - - - - - - - - - - - - - - - - - - - - - - - - - - - - - - - - - - - - - - - - - - - - - - - - - - - - - - - - - - - - - - - - - - - - - - - - - - - - - - - - - - - 1
BnbZIP42a - - - - - - - - - - - - - - - - - - - - - - - - - - - - - - - - - - - - - - - - - - - - - - - - - - - - - - - - - - - - - - - - - - - - - - - - - - - - - - - - - - - - - - - - - - - - 47
BnbZIP42b - - - - - - - - - - - - - - - - - - - - - - - - - - - - - - - - - - - - - - - - - - - - - - - - - - - - - - - - - - - - - - - - - - - - - - - - - - - - - - - - - - - - - - - - - - - - 47
BnbZIP42c - - - - - - - - - - - - - - - - - - - - - - - - - - - - - - - - - - - - - - - - - - - - - - - - - - - - - - - - - - - - - - - - - - - - - - - - - - - - - - - - - - - - - - - - - - - - 47
BnbZIP44a - - - - - - - - - - - - - - - - - - - - - - - - - - - - - - - - - - - - - - - - - - - - - - - - - - - - - - - - - - - - - - - - - - - - - - - - - - - - - - - - - - - - - - - - - - - - 3
BnbZIP44b - - - - - - - - - - - - - - - - - - - - - - - - - - - - - - - - - - - - - - - - - - - - - - - - - - - - - - - - - - - - - - - - - - - - - - - - - - - - - - - - - - - - - - - - - - - - 3
BnbZIP44c - - - - - - - - - - - - - - - - - - - - - - - - - - - - - - - - - - - - - - - - - - - - - - - - - - - - - - - - - - - - - - - - - - - - - - - - - - - - - - - - - - - - - - - - - - - - 3
BnbZIP44d - - - - - - - - - - - - - - - - - - - - - - - - - - - - - - - - - - - - - - - - - - - - - - - - - - - - - - - - - - - - - - - - - - - - - - - - - - - - - - - - - - - - - - - - - - - - 3
BnbZIP48a - - - - - - - - - - - - - - - - - - - - - - - - - - - - - - - - - - - - - - - - - - - - - - - - - - - - - - - - - - - - - - - - - - - - - - - - - - - - - - - - - - - - - - - - - - - - 54
BnbZIP48b - - - - - - - - - - - - - - - - - - - - - - - - - - - - - - - - - - - - - - - - - - - - - - - - - - - - - - - - - - - - - - - - - - - - - - - - - - - - - - - - - - - - - - - - - - - - 54
BnbZIP48c - - - - - - - - - - - - - - - - - - - - - - - - - - - - - - - - - - - - - - - - - - - - - - - - - - - - - - - - - - - - - - - - - - - - - - - - - - - - - - - - - - - - - - - - - - - - 53
BnbZIP48d - - - - - - - - - - - - - - - - - - - - - - - - - - - - - - - - - - - - - - - - - - - - - - - - - - - - - - - - - - - - - - - - - - - - - - - - - - - - - - - - - - - - - - - - - - - - 53
BnbZIP48e - - - - - - - - - - - - - - - - - - - - - - - - - - - - - - - - - - - - - - - - - - - - - - - - - - - - - - - - - - - - - - - - - - - - - - - - - - - - - - - - - - - - - - - - - - - - 54
BnbZIP48f - - - - - - - - - - - - - - - - - - - - - - - - - - - - - - - - - - - - - - - - - - - - - - - - - - - - - - - - - - - - - - - - - - - - - - - - - - - - - - - - - - - - - - - - - - - - 54
BnbZIP53a - - - - - - - - - - - - - - - - - - - - - - - - - - - - - - - - - - - - - - - - - - - - - - - - - - - - - - - - - - - - - - - - - - - - - - - - - - - - - - - - - - - - - - - - - - - - 1
BnbZIP53b - - - - - - - - - - - - - - - - - - - - - - - - - - - - - - - - - - - - - - - - - - - - - - - - - - - - - - - - - - - - - - - - - - - - - - - - - - - - - - - - - - - - - - - - - - - - 1
BnbZIP53c - - - - - - - - - - - - - - - - - - - - - - - - - - - - - - - - - - - - - - - - - - - - - - - - - - - - - - - - - - - - - - - - - - - - - - - - - - - - - - - - - - - - - - - - - - - - 1
BnbZIP53d - - - - - - - - - - - - - - - - - - - - - - - - - - - - - - - - - - - - - - - - - - - - - - - - - - - - - - - - - - - - - - - - - - - - - - - - - - - - - - - - - - - - - - - - - - - - 1
BnbZIP58a - - - - - - - - - - - - - - - - - - - - - - - - - - - - - - - - - - - - - - - - - - - - - - - - - - - - - - - - - - - - - - - - - - - - - - - - - - - - - - - - - - - - - - - - - - - - 60
BnbZIP58b - - - - - - - - - - - - - - - - - - - - - - - - - - - - - - - - - - - - - - - - - - - - - - - - - - - - - - - - - - - - - - - - - - - - - - - - - - - - - - - - - - - - - - - - - - - - 60
BnbZIP58c - - - - - - - - - - - - - - - - - - - - - - - - - - - - - - - - - - - - - - - - - - - - - - - - - - - - - - - - - - - - - - - - - - - - - - - - - - - - - - - - - - - - - - - - - - - - 60
BnbZIP58d - - - - - - - - - - - - - - - - - - - - - - - - - - - - - - - - - - - - - - - - - - - - - - - - - - - - - - - - - - - - - - - - - - - - - - - - - - - - - - - - - - - - - - - - - - - - 32
BnbZIP60a D V P T Q G - - - - - - - - - - - - - - - - - - - - - - - - - - - - - - - - - - - - - - - - - - - - - - - - - - - - - - - - - - - - - - - - - - - - - - S D D A G K E K - - - - 107
BnbZIP60b D V P T E G F V N - - - - - - - - - - - - - - - - - - - - - - - - - - - - - - - - - - - - - - - - - - - - - - - - - - - - - - - - - - - - - G K G P E E S D D A G K E K - - - - 118
BnbZIP62a L K E F Q S L E T - - - - - - - - - - - - - - - - - - - - - - - - - - - - - - - - - - - - - - - - - - - - - - - - I N K H L K E Q V A K S V K P E T K E P E E S P K A S Q V E M S 234
BnbZIP62b L K E F Q S L E T - - - - - - - - - - - - - - - - - - - - - - - - - - - - - - - - - - - - - - - - - - - - - - - - I N K H L K E Q V A K S V K P E T K E P E E S P K P S Q V E M S 231
BnbZIP62c - - - - - - - - - - - - - - - - - - - - - - - - - - - - - - - - - - - - - - - - - - - - - - - - - - - - - - - - - - - - - - - - - - - S V K P E T N E L E K P T K P S Q V E M S 64
BnbZIP36a T G S S - - - - - - - - - - - - - - - - - - - - - - - - - - - - - - - - - - - - - - - - - - - - - - - - - - - - - - - - - - - - - - G L M G - L G D Q S P T N N M G L V Q G - - 158
BnbZIP36b T G F S V Q A S - - - - - - - - - - - - - - - - - - - - - - - - - - - - - - - - - - - - - - - - - - - - P Q V V P - - - - - - - - - G L M G N L G A E T S L N - M G L V Q G - - 206
BnbZIP13a - - - - - - - - - - - - - - - - - - - - - - - - - - - - - - - - - - - - - - - - - - - - - - - - - - - - - - - - - - - - - - - E S D D V D V K I P P - E S Y G F D H H N - - - - 160
BnbZIP13b - - - - - - - - - - - - - - - - - - - - - - - - - - - - - - - - - - - - - - - - - - - - - - - - - - - - - - - - - - - - - - - E S D D V D V K I P P A E S Y G F D H H N - - - - 141
BnbZIP8a - - - - - - - - - - - - - - - - - - - - - - - - - - - - - - - - - - - - - - - - - - - - - - - - - - - - - - - - - - - - - - - - - - - - - - - - - - - - - - - - - - - - - - - - - - - - 26
BnbZIP70a - - - - - - - - - - - - - - - - - - - - - - - - - - - - - - - - - - - - - - - - - - - - - - - - - - - - - - - - - - - - - - - - - - - - - - - - - - - - - - - - - - - - - - - - - - - - 51
BnbZIP70b - - - - - - - - - - - - - - - - - - - - - - - - - - - - - - - - - - - - - - - - - - - - - - - - - - - - - - - - - - - - - - - - - - - - - - - - - - - - - - - - - - - - - - - - - - - - 51
BnbZIP70c - - - - - - - - - - - - - - - - - - - - - - - - - - - - - - - - - - - - - - - - - - - - - - - - - - - - - - - - - - - - - - - - - - - - - - - - - - - - - - - - - - - - - - - - - - - - 42
BnbZIP70d - - - - - - - - - - - - - - - - - - - - - - - - - - - - - - - - - - - - - - - - - - - - - - - - - - - - - - - - - - - - - - - - - - - - - - - - - - - - - - - - - - - - - - - - - - - - 43
BnbZIP72a - - - - - - - - - - - - - - - - - - - - - - - - - - - - - - - - - - - - - - - - - - - - - - - - - - - - - - - - - - - - - T G Y L N Y E V E P G F T I R L R Q N - - - - - - - - 44
BnbZIP72b - - - - - - - - - - - - - - - - - - - - - - - - - - - - - - - - - - - - - - - - - - - - - - - - - - - - - - - - - - - - - T G Y L N Y E V E P G F T I R L R Q N - - - - - - - - 44
BnbZIP27c - - - - - - - - - - - - - - - - - - - - - - - - - - - - - - - - - - - - - - - - - - - - - - - - - - - - - - - - - - - - - - - - - - - P P T P S S T H H G S L F S P P - - - - - 70
BnbZIP15a P S N I I H G P Y L N T N V N G - - - - - - - - - - - - - - - - - - - - - - - - - - - - - - - S T S G - - - - - Q E Q K Q H N P F Q P Q Q P I M S K P H G Y G Y G T H V A F T S 230
BnbZIP15b P I N R V H G S Y L H P N V N G - - - - - - - - - - - - - - - - - - - - - - - - - - - - - - - S T S A Y K P R Q E Q Q Q Q H N P F Q P Q Q P N M S K P H G Y G Y G T H V A F T S 234
BnbZIP35a Q A Q Q P H Q - - - - - - - - - - - - - - - - - - - - - - - - - - - - - - - - - - - - - - - - - - - - - Q Q L Q Q P - - - - - H Q R L P P T I Y P K H A N V T F S A P V - - - - 140
BnbZIP35b P A T M Q Q Q - - - - - - - - - - - - - - - - - - - - - - - - - - - - - - - - - - - - - - - - - - - - - Q Q L Q P P - - - - - H Q R L P P T I Y P K Q A N V T F A A P V N N N N 199
BnbZIP47f - - - - - - - - - - - - - - - - - - - - - - - - - - - - - - - - - - - - - - - - - - - - - - - - - - - - - - - - - - - - - - - - - - - - - - - - - - - - - - - - - - - - - - - - - - - - 53
BnbZIP19d - - - - - - - - - - - - - - - - - - - - - - - - - - - - - - - - - - - - - - - - - - - - - - - - - - - - - - - - - - - - - - - - - - - - - - - - - - - - - - - - - - - - - - - - - - - - 72
BnbZIP16f - - - - - - - - - - - - - - - - - - - - - - - - - - - - - - - - - - - - - - - - - - - - - - - - - - - - - - - - - - - - - - - - - - - - - - - - - - - - - - - - - - - - - - - - - - - - 82
BnbZIP64d - - - - - - - - - - - - - - - - - - - - - - - - - - - - - - - - - - - - - - - - - - - - - - - - - - - - - - - - - - - - - - - - - - - - - - - - - - H K T E E S D E E E - - - - 28
BnbZIP64e - - - - - - - - - - - - - - - - - - - - - - - - - - - - - - - - - - - - - - - - - - - - - - - - - - - - - - - - - - - - - - - - - - - - - - - - - - Q K T E E S E E E - - - - - 56
BnbZIP18e - - - - - - - - - - - - - - - - - - - - - - - - - - - - - - - - - - - - - - - - - - - - - - - - - - - - - - - - - - - - L G P G N D N P F P A E N G G G G G R E - E E G G R E - 101
BnbZIP18f - - - - - - - - - - - - - - - - - - - - - - - - - - - - - - - - - - - - - - - - - - - - - - - - - - - - - - - - - - - - - G P G N N N P F P A E N G G G G G R E - E E G G S S - 99
BnbZIP30c - - - - - - - - - - - - - - - - - - - - - - - - - - - - - - - - - - - - - - - - - - - - - - - - - - - - - - - - - - - - - R S R G R T R K M T N G G - - - - - - - S T G G D S - 110
BnbZIP30d - - - - - - - - - - - - - - - - - - - - - - - - - - - - - - - - - - - - - - - - - - - - - - - - - - - - - - - - - - - - - - - - - - - - - - - - - - - - - - - - - - - - - - - - - - - - 86
BnbZIP30e - - - - - - - - - - - - - - - - - - - - - - - - - - - - - - - - - - - - - - - - - - - - - - - - - - - - - - - - - - - - - S S R G R T R K M T N G G - - - - - - - S T S G D S - 175
BnbZIP2e - - - - - - - - - - - - - - - - - - - - - - - - - - - - - - - - - - - - - - - - - - - - - - - - - - - - - - - - - - - - - - - - - - - - - - - - - - - - - - - - - - - - - - - - - - - - 1
BnbZIP3h - - - - - - - - - - - - - - - - - - - - - - - - - - - - - - - - - - - - - - - - - - - - - - - - - - - - - - - - - - - - - - - - - - - - - - - - - - - - - - - - - - - - - - - - - - - - 45
BnbZIP4b - - - - - - - - - - - - - - - - - - - - - - - - - - - - - - - - - - - - - - - - - - - - - - - - - - - - - - - - - - - - - - - - - - - - - - - - - - - - - - - - - - - - - - - - - - - - 24
BnbZIP5b - - - - - - - - - - - - - - - - - - - - - - - - - - - - - - - - - - - - - - - - - - - - - - - - - - - - - - - - - - - - - - - - - - - - - - - - - - - - - - - - - - - - - - - - - - - - 47
BnbZIP5c - - - - - - - - - - - - - - - - - - - - - - - - - - - - - - - - - - - - - - - - - - - - - - - - - - - - - - - - - - - - - - - - - - - - - - - - - - - - - - - - - - - - - - - - - - - - 48
BnbZIP5d - - - - - - - - - - - - - - - - - - - - - - - - - - - - - - - - - - - - - - - - - - - - - - - - - - - - - - - - - - - - - - - - - - - - - - - - - - - - - - - - - - - - - - - - - - - - 45
BnbZIP8b - - - - - - - - - - - - - - - - - - - - - - - - - - - - - - - - - - - - - - - - - - - - - - - - - - - - - - - - - - - - - - - - - - - - - - - - - - - - - - - - - - - - - - - - - - - - 26
BnbZIP11f - - - - - - - - - - - - - - - - - - - - - - - - - - - - - - - - - - - - - - - - - - - - - - - - - - - - - - - - - - - - - - - - - - - - - - - - - - - - - - - - - - - - - - - - - - - - 1
BnbZIP11g - - - - - - - - - - - - - - - - - - - - - - - - - - - - - - - - - - - - - - - - - - - - - - - - - - - - - - - - - - - - - - - - - - - - - - - - - - - - - - - - - - - - - - - - - - - - 1
BnbZIP75a - - - - - - - - - - - - - - - - - - - - - - - - - - - - - - - - - - - - - - - - - - - - - - - - - - - - - - - - - - - - - - - - - - - - - - - - - - - - - - - - - - - - - - - - - - - - 29
BnbZIP75b - - - - - - - - - - - - - - - - - - - - - - - - - - - - - - - - - - - - - - - - - - - - - - - - - - - - - - - - - - - - - - - - - - - - - - - - - - - - - - - - - - - - - - - - - - - - 29
```

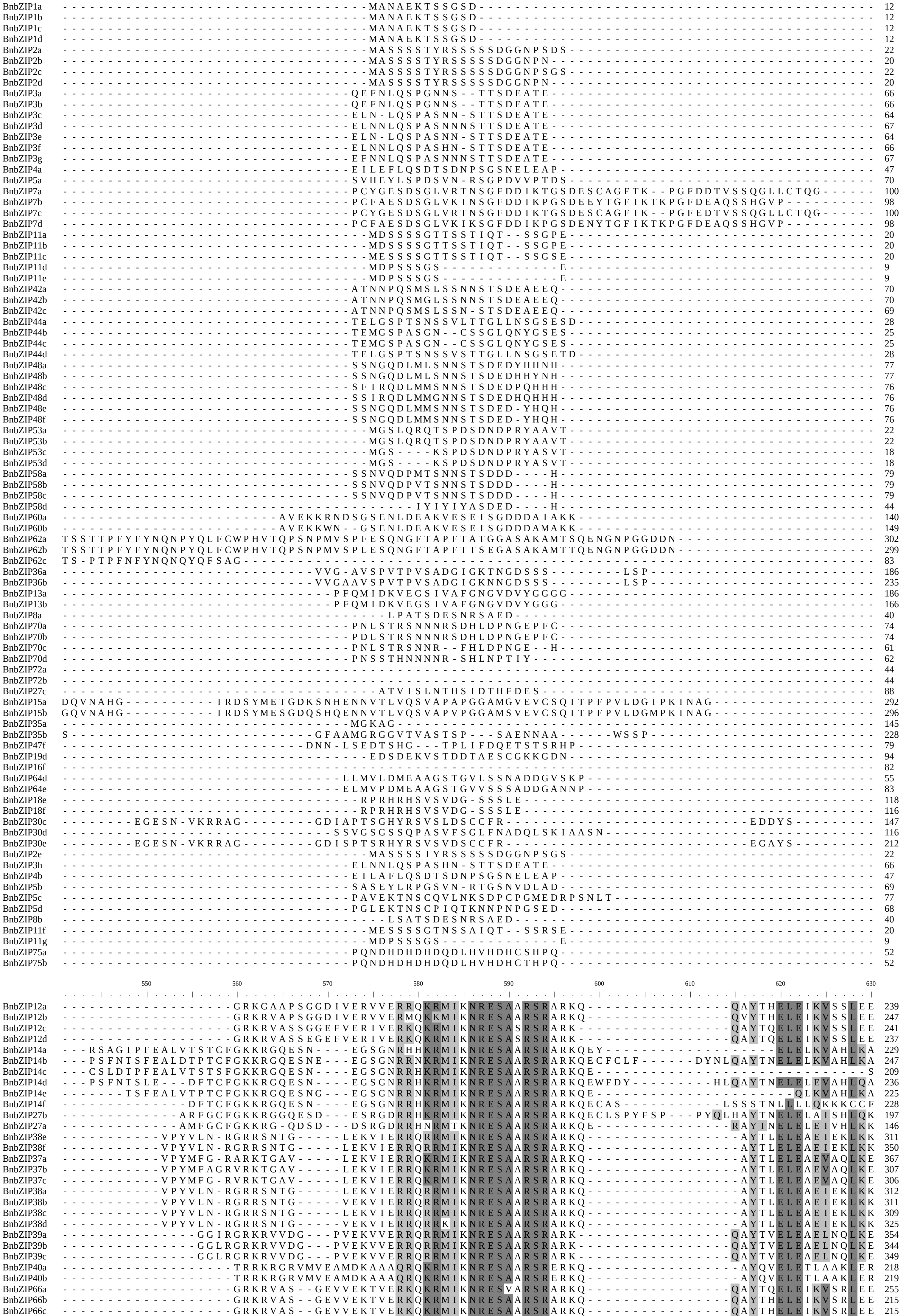

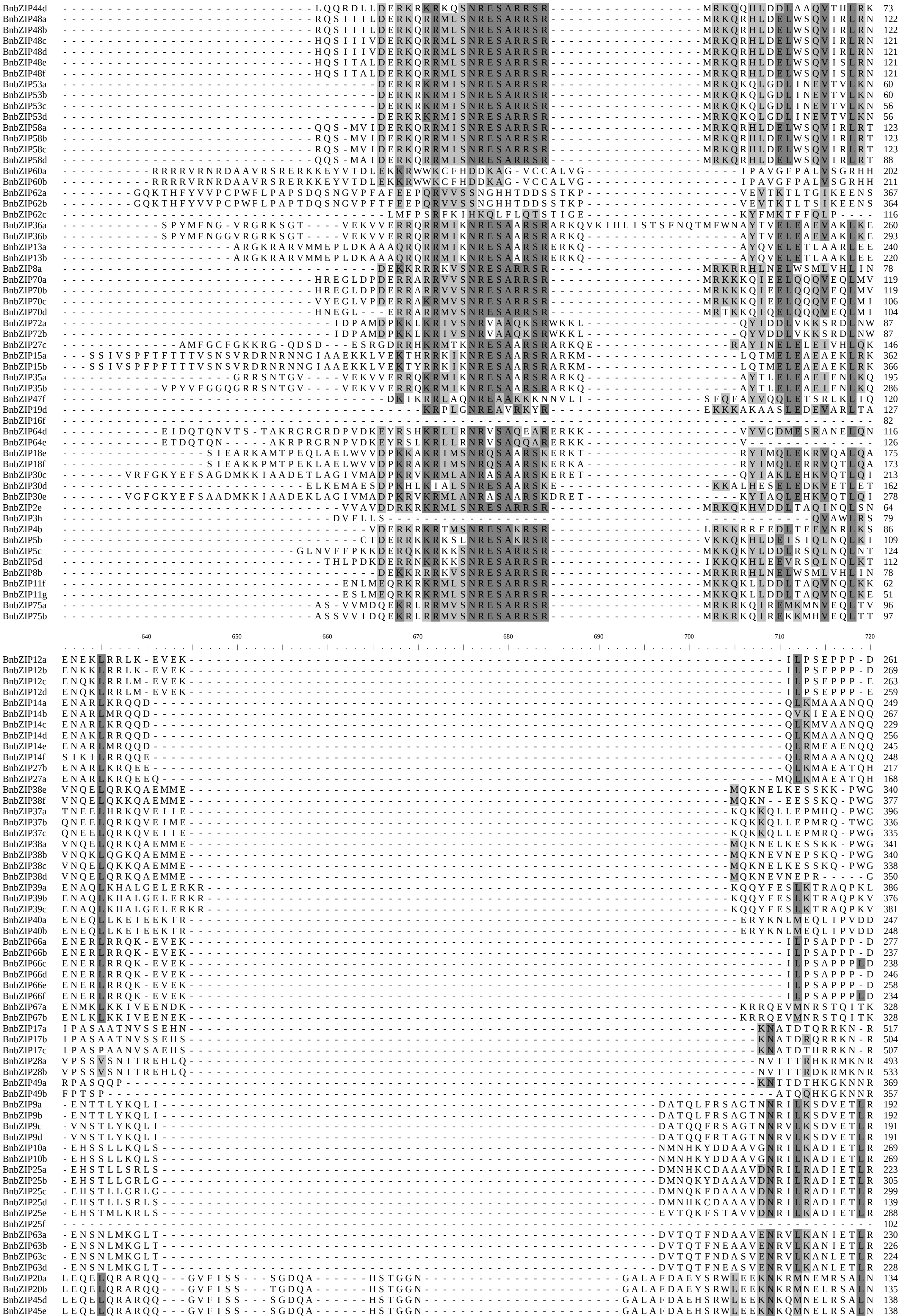
BnbZIP44d - LQQRDLLDERKRKRKQSNRESARRSR - MRKQQHLDDLAAQVTHLRK 73
BnbZIP48a - RQSIIILDERKQRRMLSNRESARRSR - MRKQRHLDELWSQVIRLRN 122
BnbZIP48b - RQSIIILDERKQRRMLSNRESARRSR - MRKQRHLDELWSQVIRLRN 122
BnbZIP48c - HQSIIIVDERKQRRMLSNRESARRSR - MRKQRHLDELWSQVIRLRN 121
BnbZIP48d - HQSIIIVDERKQRRMLSNRESARRSR - MRKQRHLDELWSQVIRLRN 121
BnbZIP48e - HQSITALDERKQRRMLSNRESARRSR - MRKQRHLDELWSQVISLRN 121
BnbZIP48f - HQSITALDERKQRRMLSNRESARRSR - MRKQRHLDELWSQVISLRN 121
BnbZIP53a - DERKRKRMISNRESARRSR - MRKQKQLGDLINEVTVLKN 60
BnbZIP53b - DERKRKRMISNRESARRSR - MRKQKQLGDLINEVTVLKN 60
BnbZIP53c - DERKRKRMISNRESARRSR - MRKQKQLGDLINEVTVLKN 56
BnbZIP53d - DERKRKRMISNRESARRSR - MRKQKQLGDLINEVTVLKN 56
BnbZIP58a - QQS-MVIDERKQRRMISNRESARRSR - MRKQRHLDELWSQVIRLRT 123
BnbZIP58b - RQS-MVIDERKQRRMISNRESARRSR - MRKQRHLDELWSQVIRLRT 123
BnbZIP58c - RQS-MVIDERKQRRMISNRESARRSR - MRKQRHLDELWSQVIRLRT 123
BnbZIP58d - QQS-MAIDERKQRRMISNRESARRSR - MRKQRHLDELWSQVIRLRT 88
BnbZIP60a - RRRRVRNRDAAVRSRERKKEYVTDLEKKRWWKCFHDDKAG-VCCALVG - IPAVGFPALVSGRHH 202
BnbZIP60b - RRRRVRNRDAAVRSRERKKEYVTDLEKKRWWKCFHDDKAG-VCCALVG - IPAVGFPALVSGRHH 211
BnbZIP62a - GQKTHFYVVPCPWFLPAPSDQSNGVPFAFEEPQRVVSSNGHHTDDSSTKP - VEVTKTLTGIKEENS 367
BnbZIP62b - GQKTHFYVVPCPWFLPAPTDQSNGVPFTFEEPQRVVSSNGHHTDDSSTKP - VEVTKTLTSIKEENS 364
BnbZIP62c - LMFPSRFKIHKQLFLQTSTIGE - KYFMKTFFQLP - 116
BnbZIP36a - SPYMFNG-VRGRKSGT-VEKVVERRQRRMIKNRESAARSRARKQVKIHLISTSFNQTMFWNAYTVELEAEVAKLKE 260
BnbZIP36b - SPYMFNGGVRGRKSGT-VEKVVERRQRRMIKNRESAARSRARKQ - AYTVELEAEVAKLKE 293
BnbZIP13a - ARGKRARVMMEPLDKAAAQRQRRMIKNRESAARSRERKQ - AYQVELETLAARLEE 240
BnbZIP13b - ARGKRARVMMEPLDKAAAQRQRRMIKNRESAARSRERKQ - AYQVELETLAAKLEE 220
BnbZIP8a - DEKKRRRKVSNRESARRSR - MRKRRHLNELWSMLVHLIN 78
BnbZIP70a - HREGLDPDERRARRVVSNRESARRSR - MRKKKQIEELQQQVEQLMV 119
BnbZIP70b - HREGLDPDERRARRVVSNRESARRSR - MRKKKQIEELQQQVEQLMV 119
BnbZIP70c - VYEGLVPDERRAKRMVSNRESARRSR - MRKKKQIEELQQQVEQLMI 106
BnbZIP70d - HNEGL-ERRARRMVSNRESARRSR - MRTKKQIQELQQQVEQLMI 104
BnbZIP72a - IDPAMDPKKLKRIVSNRVAAQKSRWKKL - QYIDDLVKKSRDLNW 87
BnbZIP72b - IDPAMDPKKLKRIVSNRVAAQKSRWKKL - QYVDDLVKKSRDLNW 87
BnbZIP27c - AMFGCFGKKRG-QDSD-ESRGDRRHKRMTKNRESAARSRARKQE - RAYINELELEIVHLQK 146
BnbZIP15a - SSIVSPFTFTTTVSNSVRDRNRNNGIAAEKKLVEKTHRRKIKNRESAARSRARKM - LQTMELEAEAEKLRK 362
BnbZIP15b - SSIVSPFPFTTTVSNSVRDRNRNNGIAAEKKLVEKTYRRKIKNRESAARSRARKM - LQTMELEAEAEKLRK 366
BnbZIP35a - GRRSNTGV-VEKVVERRQKRMIKNRESAARSRARKQ - AYTLELEAEIENLKQ 195
BnbZIP35b - VPYVFGGQGRRSNTGV-VEKVVERRQKRMIKNRESAARSRARKQ - AYTLELEAEIENLKQ 286
BnbZIP47f - DKIKRRLAQNREAAKKNNVLI - SFQFAYVQQLETSRLKLIQ 120
BnbZIP19d - KRPLGNREAVRKYR - EKKKAKAASLEDEVARLTA 127
BnbZIP16f - 82
BnbZIP64d - EIDQTQNVTS-TAKRGRGRDPVDKEYRSHKRLLRNRVSAQEARERKK - VYVGDMESRANELQN 116
BnbZIP64e - ETDQTQN-AKRPRGRNPVDKEYRSLKRLLRNRVSAQQARERKK - V - 126
BnbZIP18e - SIEARKAMTPEQLAELWVVDPKKAKRIMSNRQSAARSKERKT - RYIMQLEKRVQALQA 175
BnbZIP18f - SIEAKKPMTPEKLAELWVVDPKKAKRIMSNRQSAARSKERKA - RYIMQLEKRVQTLQA 173
BnbZIP30c - VRFGKYEFSAGDMKKIAADETLAGIVMADPKRVKRMLANRASAARSKERET - QYIAKLEHKVQTLQI 213
BnbZIP30d - ELKEMAESDPKHLKIALSNRESAARSKE - KKALHESELEDKVETLET 162
BnbZIP30e - VGFGKYEFSAADMKKIAADEKLAGIVMADPKRVKRMLANRASAARSKDRET - KYIAQLEHKVQTLQI 278
BnbZIP2e - VVAVDDRKRKRMLSNRESARRSR - MRKQKHVDDLTAQINQLSN 64
BnbZIP3h - DVFLLS - QVAWLRS 79
BnbZIP4b - VDERKRKRTMSNRESAKRSR - LRKKRRFEDLTEEVNRLKS 86
BnbZIP5b - CTDERKKRKKSLNRESAKRSR - VKKQKHLDEISIQLNQLKI 109
BnbZIP5c - GLNVFFPKKDERQKKRKKSNRESARRSR - MKKQKYLDDLRSQLNQLNT 124
BnbZIP5d - THLPDKDERRNKRKKSNRESARRSR - IKKQKHLEEVRSQLNQLKT 112
BnbZIP8b - DEKKRRRKVSNRESARRSR - MRKRRHLNELWSMLVHLIN 78
BnbZIP11f - ENLMEQRKRKRMLSNRESARRSR - MKKQKLLDDLTAQVNQLKK 62
BnbZIP11g - ESLMEQRKRKRMLSNRESARRSR - MKKQKLLDDLTAQVNQLKE 51
BnbZIP75a - AS-VVMDQEKRLRRMVSNRESARRSR - MRKRKQIREMKMNVEQLTV 96
BnbZIP75b - ASSVVIDQEKRLRRMVSNRESARRSR - MRKRKQIREKKMHVEQLTT 97
640 650 660 670 680 690 700 710 720
BnbZIP12a ENEKLRRLK-EVEK - ILPSEPPP-D 261
BnbZIP12b ENKKLRRLK-EVEK - ILPSEPPP-D 269
BnbZIP12c ENQKLRRLM-EVEK - ILPSEPPP-E 263
BnbZIP12d ENQKLRRLM-EVEK - ILPSEPPP-E 259
BnbZIP14a ENARLKRQQD - QLKMAAANQQ 249
BnbZIP14b ENARLMRQQD - QVKIEAENQQ 267
BnbZIP14c ENARLKRQQD - QLKMVAANQQ 229
BnbZIP14d ENAKLKRQQD - QLKMAAANQQ 256
BnbZIP14e ENARLMRQQD - QLRMEAENQQ 245
BnbZIP14f SIKILRRQQE - QLRMAAANQQ 248
BnbZIP27b ENARLKRQEE - QLKMAEATQH 217
BnbZIP27a ENARLKRQEEQ - MQLKMAEATQH 168
BnbZIP38e VNQELQRKQAEMME - MQKNELKESSKK-PWG 340
BnbZIP38f VNQELQKKQAEMME - MQKN-EESSKQ-PWG 377
BnbZIP37a TNEELHRKQVEIIE - KQKKQLLEPMHQ-PWG 396
BnbZIP37b QNEELQRKQVEIME - KQKKQLLEPMRQ-TWG 336
BnbZIP37c QNEELQRKQVEIIE - KQKKQLLEPMRQ-PWG 335
BnbZIP38a VNQELQRKQAEMME - MQKNELKESSKK-PWG 341
BnbZIP38b VNQKLQGKQAEMME - MQKNEVNEPSKQ-PWG 340
BnbZIP38c VNQELQKKQAEMME - MQKNELKESSKQ-PWG 338
BnbZIP38d VNQELQRKQAEMME - MQKNEVNEPR-G 350
BnbZIP39a ENAQLKHALGELERKR - KQQYFESLKTRAQPKL 386
BnbZIP39b ENAQLKHALGELERKR - KQQYFESLKTRAQPKV 376
BnbZIP39c ENAQLKHALGELERKR - KQQYFESLKTRAQPKV 381
BnbZIP40a ENEQLLKEIEEKTR - ERYKNLMEQLIPVDD 247
BnbZIP40b ENEQLLKEIEEKTR - ERYKNLMEQLIPVDD 248
BnbZIP66a ENERLRRQK-EVEK - ILPSAPPP-D 277
BnbZIP66b ENERLRRQK-EVEK - ILPSAPPP-D 237
BnbZIP66c ENERLRRQK-EVEK - ILPSAPPPLD 238
BnbZIP66d ENERLRRQK-EVEK - ILPSAPPP-D 246
BnbZIP66e ENERLRRQK-EVEK - ILPSAPPP-D 258
BnbZIP66f ENERLRRQK-EVEK - ILPSAPPPLD 234
BnbZIP67a ENMKLKKIVEENDK - KRRQEVMNRSTQITK 328
BnbZIP67b ENLKLKKIVEENEK - KRRQEVMNRSTQITK 328
BnbZIP17a IPASAATNVSSEHN - KNATDTQRRKN-R 517
BnbZIP17b IPASAATNVSSEHS - KNATDRQRRKN-R 504
BnbZIP17c IPASPAANVSAEHS - KNATDTHRRKN-R 507
BnbZIP28a VPSSVSNITREHLQ - NVTTTRHKRMKNR 493
BnbZIP28b VPSSVSNITREHLQ - NVTTTRDKRMKNR 533
BnbZIP49a RPASQQP - KNTTDTHKGKNNR 369
BnbZIP49b FPTSP - ATQQHKGKNNR 357
BnbZIP9a -ENTTLYKQLI - DATQLFRSAGTNNRILKSDVETLR 192
BnbZIP9b -ENTTLYKQLI - DATQLFRSAGTNNRILKSDVETLR 192
BnbZIP9c -VNSTLYKQLI - DATQLFRSAGTNNRILKSDVETLR 191
BnbZIP9d -VNSTLYKQLI - DATQQFRTAGTNNRVLKSDVETLR 191
BnbZIP10a -EHSSLLKQLS - NMNHKYDDAAVGNRILKADIETLR 269
BnbZIP10b -EHSSLLKQLS - NMNHKYDDAAVGNRILKADIETLR 269
BnbZIP25a -EHSTLLSRLS - DMNHKCDAAAVDNRILRADIETLR 223
BnbZIP25b -EHSTLLGRLG - DMNQKYDAAAVDNRILRADIETLR 305
BnbZIP25c -EHSTLLGRLG - DMNQKFDAAAVDNRILRADIETLR 299
BnbZIP25d -EHSTLLSRLS - DMNHKCDAAAVDNRILRADIETLR 139
BnbZIP25e -EHSTMLKRLS - EVTQKFSTAVVDNRILKADIETLR 288
BnbZIP25f - 102
BnbZIP63a -ENSNLMKGLT - DVTQTFNDAAVENRVLKANIETLR 230
BnbZIP63b -ENSNLMKGLT - DVTQTFNEAAVENRVLKANIETLR 226
BnbZIP63c -ENSNLMKGLT - DVTQTFNDASVENRVLKANIETLR 224
BnbZIP63d -ENSNLMKGLT - DVTQTFNEASVENRVLKANLETLR 228
BnbZIP20a LEQELQRARQQ-GVFISS-SGDQA-HSTGGN - GALAFDAEYSRWLEEKNKRMNEMRSALN 134
BnbZIP20b LEQELQRARQQ-GVFISS-TGDQA-HSTGGN - GALAFDAEYSRWLEEKNKRMNELRSALN 135
BnbZIP45d LEQELQRARQQ-GVFISS-SGDQA-HSTGGN - GALAFDAEHSRWLEEKNKQMNELRSALN 138
BnbZIP45e LEQELQRARQQ-GVFISS-SGDQA-HSTGGN - GALAFDAEHSRWLEEKNKQMNELRSALN 138

```
BnbZIP15a  ENHELLKNQGKEITN--------------------------------------------------------------------------G  378
BnbZIP15b  ENHELLKKQGKKITN--------------------------------------------------------------------------G  382
BnbZIP35a  LNQDLQRKQAEIMK------------------------------------------------------------TQKNE--EPSKQRPWL  223
BnbZIP35b  LNQDLQRKQAEIMK------------------------------------------------------------TQKNELKEPSKQRPWL  316
BnbZIP47f  LEQELDRARQQ---GFYVGN---GIDTSTTTSLGFSENMNP---------------------GIAAFEMEYGQWIEEQNKQICELRTVLQ  183
BnbZIP19d  VNHQLVKRLQS----------------------------------------------------QGALEAEVSRLK----CLLVDLRGRID  161
BnbZIP16f  ------------------------------------------------------------------------------------------  82
BnbZIP64d  NNNELEEKISTLMN---------------------------------------------------------------ENTMLRKMLINTR  143
BnbZIP64e  ------------------------------------------------------------------------------------------  126
BnbZIP18e  EYATRSAYLSLINGDID--------------------------------------------------------GLLSENTKLKLQIQMVE  209
BnbZIP18f  EYATRSAHLSLINTDIA--------------------------------------------------------GLLSENTELKLRIQAVE  207
BnbZIP30c  ETTTLSAHLTYLQRDNM--------------------------------------------------------GLQNQNNELKFRLQSME  247
BnbZIP30d  QIDILTAELKLE--------------------------------------------------------KRERMTADDECVQFRIRLHAGE  196
BnbZIP30e  ETTTLSAHLTHLQRDNM--------------------------------------------------------GLQNQNSELKFRLQSME  312
BnbZIP2e   -DNRQILTSLT-------------------------------------------------------VTTQLYTKIQAENSILTAQMTELS  98
BnbZIP3h   -ENHQILDKLN-------------------------------------------------------KASDSNDLVLQENVILKEENLELR  113
BnbZIP4b   -KNQELVNQLA-------------------------------------------------------NVLSCGNAISKENNRLKTESVCLE  120
BnbZIP5b   -QNRKLKNQLQ-------------------------------------------------------YIYYHCQRTKMENDRLRLEHRMLH  143
BnbZIP5c   -ENRELENQLR-------------------------------------------------------YVMHHCQGANMENDRLRVENQILH  158
BnbZIP5d   -ENRDLLNRLR-------------------------------------------------------YFMHNCQHAKMESDRLRLEHKVLL  146
BnbZIP8b   -ENKCLVDELS-------------------------------------------------------RANEGYEDVVEENKKLREENSKLR  112
BnbZIP11f  -ENSEIVTSVS-------------------------------------------------------ITTQHYLAVEAENSVLRAQLDELS  96
BnbZIP11g  -QNNEILTSVS-------------------------------------------------------ITTQHYLTVEAENSVLKAQLDELS  85
BnbZIP75a  DANYNLYNKVI-------------------------------------------------------SLLEKTRQILHENSQLEEKLSYFH  131
BnbZIP75b  -SNYNLYNKVI-------------------------------------------------------SLLERTPQILHENSQLEEKLSYFH  131

                    730       740       750       760       770       780       790       800       810
           ....|....|....|....|....|....|....|....|....|....|....|....|....|....|....|....|....|....|

BnbZIP12a  PKWK-------------------------LRRTSSASF----------------------------------------------------  274
BnbZIP12b  PKWK-------------------------LRRTSSASF----------------------------------------------------  282
BnbZIP12c  PKWK-------------------------LRRTSSASF----------------------------------------------------  276
BnbZIP12d  PKWK-------------------------LRRTSTASF----------------------------------------------------  272
BnbZIP14a  PKKN------------------------TLQRSSSALF----------------------------------------------------  263
BnbZIP14b  PKKN------------------------TLQRASTAPF----------------------------------------------------  281
BnbZIP14c  PKKN------------------------TLQRSSTAPF----------------------------------------------------  243
BnbZIP14d  PKKK------------------------TLQRSSTSPF----------------------------------------------------  270
BnbZIP14e  PKKN------------------------TLQRASTAPF----------------------------------------------------  259
BnbZIP14f  PKKK------------------------TLQRSSTAPF----------------------------------------------------  262
BnbZIP27b  QTKR------------------------KLQRSWTTPF----------------------------------------------------  231
BnbZIP27a  QTKK------------------------ILQRSWTSPF----------------------------------------------------  182
BnbZIP38e  SKRQ------------------------CLRRTLTGPW----------------------------------------------------  354
BnbZIP38f  SKRQ------------------------CLRRTLTGPW----------------------------------------------------  391
BnbZIP37a  CKRQ------------------------CLRRTSTGPW----------------------------------------------------  410
BnbZIP37b  CKRQ------------------------CLRRTLTGPW----------------------------------------------------  350
BnbZIP37c  CKRQ------------------------CLRRTLTGPW----------------------------------------------------  349
BnbZIP38a  SKRQ------------------------CLRRTLTGPW----------------------------------------------------  355
BnbZIP38b  SKRQ------------------------CLRRTLTGPW----------------------------------------------------  354
BnbZIP38c  SKRQ------------------------CLRRTLTGPW----------------------------------------------------  352
BnbZIP38d  SKRQ------------------------CLRRTLTGPW----------------------------------------------------  364
BnbZIP39a  PKASG--------------------RLRTLVRNPSCPL----------------------------------------------------  404
BnbZIP39b  PKVSG--------------------RLRTLMRNPSCPL----------------------------------------------------  394
BnbZIP39c  PKVSG--------------------RLRTLMRNPSCPL----------------------------------------------------  399
BnbZIP40a  EKRKPSS-----------------SSSRSLSRSHSLEW----------------------------------------------------  268
BnbZIP40b  EKRKPSS-----------------SASRSLSRSHSLEW----------------------------------------------------  269
BnbZIP66a  PKRQ-------------------------LRRTSSAPF----------------------------------------------------  290
BnbZIP66b  PKRQ-------------------------LRRTSSAPF----------------------------------------------------  250
BnbZIP66c  PKRQ-------------------------LRRTSSAPF----------------------------------------------------  251
BnbZIP66d  PKRQ-------------------------LRRTSSAPF----------------------------------------------------  259
BnbZIP66e  PKRQ-------------------------LRRTSSAPF----------------------------------------------------  271
BnbZIP66f  PKRQ-------------------------LRRTSSAPF----------------------------------------------------  247
BnbZIP67a  EKNGD--------------------KLRRIRRMASAGW----------------------------------------------------  346
BnbZIP67b  EKNGD--------------------KLRRIRRMASAGW----------------------------------------------------  346
BnbZIP17a  RTLRGL-------------------AIPLPGS------EHQRNSSSKE-------IKPVS-SMVVS------------------------  550
BnbZIP17b  RTLRGL-------------------AIPLPGSDFNFTKEHQRNSSSKE-------IKAAS-SMVVS------------------------  543
BnbZIP17c  RTLRGL-------------------PIPLPGSDFNFTKEHQRNSSSKE-------IKPAS-SMVVS------------------------  546
BnbZIP28a  RILEGL-------------------PVSRLASELNITEAQASKDAQNKSFHGKDNTKPTSSSMVVS------------------------  540
BnbZIP28b  RILEGL-------------------PVSRLASELNITEAQASKDAQNKTFHGKANTKPTSSSMVVS------------------------  580
BnbZIP49a  RILRG--------------------GHPLSDSNLTKDQNNSSKDNFST-------TKPFP-SMVVS------------------------  407
BnbZIP49b  RILRG--------------------GHPLSNLNLSKDQNSSNKENSST-------NKSFP-SMVVS------------------------  395
BnbZIP9a   IKVKLAEDLVARGSLTSSL-----------NQLLQTHLSPSPQSIN--------------------------------------------  227
BnbZIP9b   IKVKLAEDLVARGSFTSSL-----------NQLLQTHLSPSPQSIN--------------------------------------------  227
BnbZIP9c   VKVKLAEDLIARGSLTSSL-----------NQLLQTHLSPPQHSIN--------------------------------------------  226
BnbZIP9d   VKVKLAEDLIARGSLTSSL-----------NQLLQTHLSPPQHSIN--------------------------------------------  226
BnbZIP10a  AKVKMAEETVKRVTGMNPM-----------LLGRSNGHNNNNN-RMPLTGNSRMGG------------------SSCIPPFQPQSNPNMG  329
BnbZIP10b  AKVKMAEETVKRVTGMNPM-----------LLGRSNGHNNNNNNRMPLTGNSRMG-------------------SSCIPPFQPQSNLNMG  329
BnbZIP25a  TKVKMVEETVKRVTGVNPL-----------HWARPNMGTPLNNTPIDSS----------------------------------RILPNSN  268
BnbZIP25b  TKVKMAEETVKRVTGVNPL-----------QWARPNMGMPVNNTPR--------------------------------------------  340
BnbZIP25c  TKVKMAEETVKRVTGVNPL-----------QWARPNMGMPVNNTPR--------------------------------------------  334
BnbZIP25d  TKIMILTKFMNPTD----------------------------------------------------------------------------  153
BnbZIP25e  TKVKMAEDTVKRVTGMNPR-----------LLAKP-YSIPFDRTLMGS------------------------------------------  324
BnbZIP25f  ---KMAEETVKRVTRVNPS-----------RWTRTNMDIPLKR-----------------------------------------------  131
BnbZIP63a  AKVKMAEETVKRLTGFNPM-----------FQTMPQVSTVSN------------------------------------------------  261
BnbZIP63b  AKVKMAEETVKRLTGFNPM-----------YHTMPQVSTVSN------------------------------------------------  257
BnbZIP63c  AKVKMAEETVKRITGFNPM-----------FHTMPQVSSVSN------------------------------------------------  255
BnbZIP63d  AKVKMAEETVKRITGFNPM-----------FHTMPPVSTVSN------------------------------------------------  259
BnbZIP20a  AHAGDGELLIIVDG--VMAHYEELFRIKSNAAKNDVFHLLYGMWK---------------------------------------------  177
BnbZIP20b  AHAGDGELLIIVDG--VMAHYEELFRIKSNAAKNDVFHLLYGMWK---------------------------------------------  178
BnbZIP45d  AHAGDTELRIIVEG--VMSHYEELFRIKSNAAKNDVFHLLSGMWK---------------------------------------------  181
BnbZIP45e  AHAGDTELRIIVEG--VMSHYEELFRIKSSAAKNDVFHLLSGMWK---------------------------------------------  181
BnbZIP45a  AHAGDAELLIIVDG--VMTHYEELFRIKSNAAKNDVFHLLSGMWK---------------------------------------------  184
BnbZIP45b  AHAGDTELRTIVDG--AMAHYEELFRIKSNAAKNDVFHLLSGMWK---------------------------------------------  181
BnbZIP45c  AHAGDAELLIILDG--VMTHYEELFRIKSNAAKNDVFHLLSGMWK---------------------------------------------  183
BnbZIP45f  AHAGDTELRTIVDG--VMAHYEELFRIKSNAAKNDVFHILSGMWK---------------------------------------------  204
BnbZIP26a  SHASEPELRTIVEA--VLAHYEELFRIKSNAAKNDVFHLLSGMWK---------------------------------------------  176
BnbZIP26b  SHASEPELRTIVEA--VLAHYEELFRIKSNAAKNDVFHLLSGMWK---------------------------------------------  176
BnbZIP21a  AHLSDNDLRLIVDG--YIAHFDEVLRLKAVAAKADVFHLIIGTWM---------------------------------------------  308
BnbZIP21b  AHLSDNDLRLIVDG--YIAHFDEVLRLKAVAVKADVFHLIIGTWM---------------------------------------------  308
BnbZIP21c  AHLSDNDLRLIVDG--YIAHFDEVFRLKAVAAKADVFHLIIGTWM---------------------------------------------  314
BnbZIP21d  AHLSDNDLRLIVDG--YIAHFDEVFRLKAVAAKADVFHLIIGTWM---------------------------------------------  258
BnbZIP22a  AHISDIELKMLVDI--CLNNYANLFRMKADAAKADVFFLISGMWR---------------------------------------------  237
BnbZIP22b  AHISDIELKMLVDI--CLNHYANLFRMKADAAKADVFFLISGMWR---------------------------------------------  270
BnbZIP22c  AHISDVELKMLVDV--CLNHYANLFRMKAAAAKADVFFLISGMWR---------------------------------------------  227
BnbZIP22d  AHISDVELKMLVDV--CLNHYANLFRMKADAAKADVFFLISGMWR---------------------------------------------  226
BnbZIP50c  AHISDIELKMLVES--CLNHYANLFRMKADAAKADVFYLISGMWR---------------------------------------------  224
BnbZIP50d  AHISDIELKMLVES--CLNHYANLFRMKSDAAKADVFYLISGMWR---------------------------------------------  224
BnbZIP46a  SQLGDNELRVLVDA--VMSHYDEIFRLKGIGTKVDVFHMLSGMWQ---------------------------------------------  292
BnbZIP46b  SQLGDNELRVLVDA--VMSHYDEIFRLKGIGTKVDVFHMLSGMWQ---------------------------------------------  292
BnbZIP47a  GQVSDVELRLLVEN--GMKHYFDLFRMKSAAAKADVFFVMSGMWR---------------------------------------------  224
BnbZIP47b  GHVTDVELRSLVEN--TMKHYFELFRMKSAAAKADVFFVMSGMWR---------------------------------------------  221
BnbZIP47c  GQVSDVELRLLVEN--GMKHYFDLFRMKSAAAKADVFFVMSGMWR---------------------------------------------  224
BnbZIP47d  GHVTDVELRSLVEN--TMKHYFELFRMKSAAAKADVFFVMSGMWR---------------------------------------------  221
BnbZIP47e  GHVGDVELRLLVEI--AMKHYFDLFRMKSAAAKEDVFFVMSGMWR---------------------------------------------  243
BnbZIP50b  AHISDIELRMLVES--CLNHYANLFRMKSDAAKADVFYLISGMWR---------------------------------------------  285
BnbZIP50a  AHISDIELRMLVES--CLNHYANLFRMKSDAAKADVFYLISGMWR---------------------------------------------  299
BnbZIP57a  GQVSDVELRLLVDN--AMKHYFQLFRMKSAAAKIDVFYIMSGMWK---------------------------------------------  216
BnbZIP57b  GQVSDVELRLLVDN--AMKHYFQLFRMKSAAAKIDVFYIMSGMWK---------------------------------------------  216
BnbZIP57c  GQVSDIELRLLVDN--AMKHYFQLFRMKSAAAKLDVFYIMSGMWK---------------------------------------------  218
BnbZIP57d  GQVSDVEIRLLVDN--AMKHYFQLFLMKSAAAKLDVFYIMSGMWK---------------------------------------------  218
BnbZIP65a  EHLAENELRMFVDT--CLAHYDHLINLKAMVAKTDVFHLISGAWK---------------------------------------------  296
BnbZIP65b  EHLAENELRMFVDT--CLAHYDHLINLKAMVAKTDVFHLISGAWK---------------------------------------------  296
BnbZIP65c  EHLAENELRMFVDN--CLAHYDHLINLKAMVAKTDVFHLISGAWK---------------------------------------------  221
```

```
BnbZIP14a  ------------------------------------------------------------------------------------------  263
BnbZIP14b  ------------------------------------------------------------------------------------------  281
BnbZIP14c  ------------------------------------------------------------------------------------------  243
BnbZIP14d  ------------------------------------------------------------------------------------------  270
BnbZIP14e  ------------------------------------------------------------------------------------------  259
BnbZIP14f  ------------------------------------------------------------------------------------------  262
BnbZIP27b  ------------------------------------------------------------------------------------------  231
BnbZIP27a  ------------------------------------------------------------------------------------------  182
BnbZIP38e  ------------------------------------------------------------------------------------------  354
BnbZIP38f  ------------------------------------------------------------------------------------------  391
BnbZIP37a  ------------------------------------------------------------------------------------------  410
BnbZIP37b  ------------------------------------------------------------------------------------------  350
BnbZIP37c  ------------------------------------------------------------------------------------------  349
BnbZIP38a  ------------------------------------------------------------------------------------------  355
BnbZIP38b  ------------------------------------------------------------------------------------------  354
BnbZIP38c  ------------------------------------------------------------------------------------------  352
BnbZIP38d  ------------------------------------------------------------------------------------------  364
BnbZIP39a  ------------------------------------------------------------------------------------------  404
BnbZIP39b  ------------------------------------------------------------------------------------------  394
BnbZIP39c  ------------------------------------------------------------------------------------------  399
BnbZIP40a  ------------------------------------------------------------------------------------------  268
BnbZIP40b  ------------------------------------------------------------------------------------------  269
BnbZIP66a  ------------------------------------------------------------------------------------------  290
BnbZIP66b  ------------------------------------------------------------------------------------------  250
BnbZIP66c  ------------------------------------------------------------------------------------------  251
BnbZIP66d  ------------------------------------------------------------------------------------------  259
BnbZIP66e  ------------------------------------------------------------------------------------------  271
BnbZIP66f  ------------------------------------------------------------------------------------------  247
BnbZIP67a  ------------------------------------------------------------------------------------------  346
BnbZIP67b  ------------------------------------------------------------------------------------------  346
BnbZIP17a  ----------------VLVDPRE-GGDGDIDGMIGGPKSLSRVFVVVLQDSAKYVTYSCVLPRPG-APHLMTT-----------------  605
BnbZIP17b  ----------------VLVDPRE-GGDGDIDGMIGGPKSLSRVFVVVLVDSAKYVTYSCVLPRPG-APHLVTT-----------------  598
BnbZIP17c  ----------------VLVDPRE-GGDGDIDGMIGGPKSLSRVFVVVLVDSAKYVTYSCVLPRSG-APHLVTT-----------------  601
BnbZIP28a  ----------------VLLDPREVVDSETDRVMPSNPKSLSRIFVVVLLDSVKYVTYSCVLPRSG--LHLVAT-----------------  595
BnbZIP28b  ----------------VLLDPREVVDSETDRVMPSNPKSLSRVFVVVLLDSVKYVTYSCVLPRSG--LHLVAT-----------------  635
BnbZIP49a  ----------------VLTDPREEGSDEDINGVTGDTKSLSRLFIVVLVDSVKYVTYSCVLPRPEEVPHLVTT-----------------  464
BnbZIP49b  ----------------VLIDPREESSD--------------GVFIVVLVDNVKYVTYSCVLPRPEEVPHLVTT-----------------  438
BnbZIP9a   ------SLHYTG-STSPAVTVHSDQSMFPGITLSGQNSSPGLGNVSSEAVSCVSDIWP--------------------------------  278
BnbZIP9b   ------SLHYTG-STSPAVTVHSDQSMFPGITLSGQNSSSGLGNVSSEAVSCVSDIWP--------------------------------  278
BnbZIP9c   ------SLHYTGNNTSPAITVHSDQSMFPGITISGQNSSPGLGNVSSEAVSCVSDIWP--------------------------------  278
BnbZIP9d   ------SLHYTGNNTSPAITVHSDQSLFPGITISGQNSSPGLGNVSSEAVSCVSDIWP--------------------------------  278
BnbZIP10a  GLTTTILSPRLENSFIPTPSLNSQTNSPHLQRIRPTQNHHAAPTTNTYGWTTETHNDSAWPKKCVD------------------------  395
BnbZIP10b  GLPTTILSPRLENSFIPTPSLNSQTNS-HLQRIRPTQNHHAAPTTNPYGWTTETHNDSTWPKKCVD------------------------  394
BnbZIP25a  HMLEPAIPSTTNAGLASNQRVERANLLLEQVNREGMQNQFAINP---NLY----------------------------------------  315
BnbZIP25b  ------IPSTTGAGSAPNQRVE--------VNREGLQNQFATNPK--NMYETMPHWNHKH------------------------------  384
BnbZIP25c  ------ISSTTGASLAPNQRVE--------VNSEGLQNQFATNPK--NMYETMPHWNHKH------------------------------  378
BnbZIP25d  -------QRVERANLLPEQ-----------VNREGMQNQFATNP---NLYETLLHWNHKH------------------------------  192
BnbZIP25e  -----SQPNLNQTDMIPNQISE---------DNSFASNSVVTLESGCNAFEATSWICDKDAP----------------------------  372
BnbZIP25f  -----AIPSTADAGLGPYQRVDKPDFLPEQLNRESIQNQIDTNP---NIFETLPHWRHKH------------------------------  183
BnbZIP63a  -------PSETSGSVDTSVQVTTPEISSG-NKNKALTGCKMNRTESMRRVASLEHLQKRIRSVGDQ------------------------  319
BnbZIP63b  -------PSETSDSVDTSIRVTTSEISSG-NKNKALTGCKMNRTESMRRVASLEHLQKRIRSVGDQ------------------------  315
BnbZIP63c  -------PSETSNSLDTSSQVTTPEISSSSNKSKALMGCKMNRTESMRRVASLEHLQKRIRSVGDK------------------------  314
BnbZIP63d  -------PSETSNSLDTSSQVTTPEISSSSNKSKALIGCKMNRTESMRRVASLEHLQKRIRSVGDK------------------------  318
BnbZIP20a  -------TPAERCFLWLGGFRSSEILKLLVNQLEPMTERQVMGINSLQETSQQAEDALSQGMESLQQS-----LADTLSSGTLG---SSS  252
BnbZIP20b  -------TPAERCFLWLGGFRSSEILKLLVNQLEPMTERQVMGINSLQETSQQAEDALSQGMESLQQS-----LADTLSSGTLG---SSS  253
BnbZIP45d  -------TPAERCFLWLGGFRSSELLKLIANQLEPMTERQVMGLNSLQQTSQQAEDALSQGMESLQQS-----LADTLSSGTLG---SSS  256
BnbZIP45e  -------TPAERCFLWLGGFRSSELLKLIANQLEPITERQVMGLNSLQQTSQQAEDALSQGMESLQQS-----LADTLSSGTLG---SSS  256
BnbZIP45a  -------TPAERCFLWLGGFRSSELLKLLANQLEPMTERQVMGLNSLQQTSQQAEDALSQGMESLQQS-----LADTLSSGTLG---SSS  259
BnbZIP45b  -------TPAERCFLWLGGFRSSELLKLLANQLEPMTERQVLGINSLQQTSQQAEDALSQGMESLQQS-----LAETLSSGTLG---SSS  256
BnbZIP45c  -------TPAERCFLWLGGFRSSELLKLLANQLEPVTERQVMGLNSLQQTSQQAEDALSQGMESLQQS-----LADTLSSGTLG---SSS  258
BnbZIP45f  -------TPAERCFLWLGGFRSSELLKLLANQLEPMTERQVLGINSLQQTSQQAEDALSQGMESLQQS-----LAETLSSGTLG---SCS  279
BnbZIP26a  -------TPAERCFLWLGGFRSSDLLKLIASQVEPLTEQQSQDLNNLQESSQQAEDALSQGMDSLQQS-----LSETLSSGTLG---SSS  251
BnbZIP26b  -------TPAERCFLWLGGFRSSDLLKLIASQVEPLTEQQSQDLNNLQESSQQAEDALSQGMDSLQQS-----LSETLSSGTLG---SSS  251
BnbZIP21a  -------SPAERCFIWMAGFRPSDLIKILVSQMDLLTEQQLMGIYSLQHSSQQAEEALSQGLEQLQQS-----LIDTLAAS---------  377
BnbZIP21b  -------SPAERCFIWMAGFRPSDLIKILVSQMDLLTEQQLMGIYSLQHSSQQAEEALSQGLEQLQQS-----LIDTLAAS---------  377
BnbZIP21c  -------SPAERCFIWMAGFRPSDFIKILVSQMDLLTEQQLMGIYSLQHSSQQAEEALSQGLEQLQQS-----LIDTLAAS---------  383
BnbZIP21d  -------SPAERCFIWMAGFRPSDFIKILVSQMDLLTEQQLMGIYSLQHSSQQAEEALSQGLEQLQQS-----LIDTLAAS---------  327
BnbZIP22a  -------TSTERFFQWIGGFRPSELLNVVMPYIEPLTDQQLLEVRNLQQSSQQAEEALSQGLDKLQQG-----LVENIAVDIR------V  309
BnbZIP22b  -------TSTERFFQWIGGFRPSELLNVVMPYIEPLTDQQLLEVRNLQQSSQQAEEALSQGLDKLQQG-----LVENIAVDIR------V  342
BnbZIP22c  -------TSTERFFQWIGGFRPSELLNVVMPYIEPLTDQQLLEVTNLQQSSQQAEEALSQGLDKLQQG-----LVENIAV----------  295
BnbZIP22d  -------TSTERFFQWIGGFRPSELLNVVMPYIEPLTDQQLLEVRNLQQSSQQAEEALSQGLDKLQQG-----LVENIAV----------  294
BnbZIP50c  -------TSTERFFQWIGGFRPSGLLNVVMPYLQPLTDQQILEVRNLQQSSQQAEDALSQGIDKLQQS-----LADNIVID--------V  294
BnbZIP50d  -------TSTERFFQWIGGFRPSGLLNVVMPYLQPLTDQQILEVRNLQQSSQQAEDALSQGIDKLQQS-----LAENIVID--------V  294
BnbZIP46a  -------TPAERFFMWLGGFRSSELLKILGNHVDPLTDQQLIGICNLQQSSQQAEDALSQGMEALQQS-----LLETLSSASMG---PNS  367
BnbZIP46b  -------TPAERFFMWLGGFRSSELLKILGNHVDPLTDQQLIGICNLQQSSQQAEDALSQGMEALQQS-----LLETLSSASMG---PNS  367
BnbZIP47a  -------TSAERFFLWIGGFRPSDLLKVLLPHFDVMTDQQILDVCNLRQSCQQAEDALSQGMEKLQHT-----LADCVAGG---------  293
BnbZIP47b  -------TSAERFFLWIGGFRPSDLLKVLLPHFDVMTDQQVLDVCNLRQSCQQAEDALSQGMEKLQHT-----LAECVARG---------  290
BnbZIP47c  -------TSAERFFLWIGGFRPSDLLKVLLPHFDVMTDQQILDVCNLRQSCQQAEDALSQGMEKLQHT-----LADCVAGG---------  293
BnbZIP47d  -------TSAERFFLWIGGFRPSDLLKVLLPHFDVMTDQQVLDVCNLRQSCQQAEDALSQGMEKLQHT-----LAECVARG---------  290
BnbZIP47e  -------TSAERFFLWIGGFRPSDLIKVLLPHFDVMTDQQILDVCNLRQSCQQAEDALSQGMEKLQHT-----LADCVAGG---------  312
BnbZIP50b  -------TSTERFFQWIGGFRPSELLNVVMPYLQPLTDQQVLEVRNLQQSSQQAEEALSQGIEKLQQS-----LVENIVVD--------A  355
BnbZIP50a  -------TCTERFFQWIGGFRPSELLNVVMPYLQPLTDQQVLEVRNLQQSSQQAEDALSQGIEKLQQS-----LVENIVVD--------A  369
BnbZIP57a  -------TSAERFFMWIGGFRPSELLKVLLPHFDPLMDQQVLDVCNLRQSCQQAEDAVSQGMEKLQHT-----LADSVAAG---------  285
BnbZIP57b  -------TSAERFFMWIGGFRPSELLKVLLPHFDPLMDQQVLDVCNLRQSCQQAEDAVSQGMEKLQHT-----LADSVAAG---------  285
BnbZIP57c  -------TSAERFFLWIGGFRPSELLKVLLPHFDPMMDQQVLDVCNLRQSCQQAEDAVSQGMEKLQHT-----LAESVAAG---------  287
BnbZIP57d  -------TSAERFFLWIGGFRPSELLKVLVPHFDPMMDQQVLDVCNLRQSCQQAEDAVSQGMEKLQHT-----LAESVAAG---------  287
BnbZIP65a  -------TPAERCFLWMGGFRPSEIIKVIVNQIEPLTEQQIVGICGLQQSTHEAEEALSQGLEALNQS-----LSDSIVSDSLSPASAQL  374
BnbZIP65b  -------TPAERCFLWMGGFRPSEIIKVIVNQIEPLTEQQIVGICGLQQSTQEAEEALSQGLEALNQS-----LSDSIVSDSLSPASAQL  374
BnbZIP65c  -------SPAERCFLWMGGFRPSEIIKVIVNQIEQLTEQQIVGICGLQQSTQEAEEALSQGLEALNQS-----LSDSIVSDSLSPASAQF  299
BnbZIP34a  -------------------------------------MENANHSPETG------VDIKLSIEKEQMLNI---------------------  268
BnbZIP34b  -------------------------------------MENANHLPETG------VDIKLSIEKEQMLNI---------------------  268
BnbZIP34c  -------------------------------------MDNANHLQATGTVANSAVDIKPSNEN---------------------------  273
BnbZIP34d  -------------------------------------MENGNHSQATGTGANSAVDIKPSNEKEELFNV---------------------  280
BnbZIP34e  -------------------------------------MENGNHSQATGTGANSAVDIKPSNEKEELFNV---------------------  279
BnbZIP61a  -------------------------------------MENNVSDQSP-------ADIKPSVEKEQLLNA---------------------  298
BnbZIP61b  -------------------------------------MENNVSDQSP-------ADIKPSVEKKQ-LNA---------------------  267
BnbZIP61c  -------------------------------------MENNVSDQSP-------ADIKPSVEKEQ-LNA---------------------  284
BnbZIP19a  -------SSQ-----GGVSIN----DGCGFDQLQQCVANQNLVGNGNGSFNSSANASA-SNKRKGG------------------------  251
BnbZIP19b  -------GGNSQEGGGGASINEQGLSGCDFDQLQ-CMTDQNLNG----SFN-SANVSA-TNKRKGG------------------------  264
BnbZIP19c  -------GNNRQEGGG------------DFDQLQ-CMTDQNLNG----SFN-SANVSA-SNKRKGG------------------------  231
BnbZIP23b  -------EGILMNG----------LNGCEFDQLQCLG-DQNLAGCSNENGTFNVDASG-ANKSKGG------------------------  232
BnbZIP23a  -------EGILMNGQG--------LNGCEFDQLQCLG-DQNLAGCSNGNGTFDVDASG-ANKSKGG------------------------  234
BnbZIP23c  -------EGALMNGQG--------LNGCEFDQLQCLDGDQNLAGCSNGNGTFGVNASGVANKRKRG------------------------  241
BnbZIP23d  -------EGALMNGQG--------MNGCEFDQLQCLGGDQDLAGCSNGNGTFGVNASGVGNKRKRG------------------------  232
BnbZIP24a  -------ATRN--------------MICEVARVECEE-GKTLHEPVHSFVPHSPPFSR--------------------------------  188
BnbZIP24b  -------ATRN--------------MICEVARVECEE-GKTLHEPIHSFVPHSPPFSR--------------------------------  188
BnbZIP16a  ------------------------------------------------------------------------------------------  459
BnbZIP16b  ------------------------------------------------------------------------------------------  395
BnbZIP16c  ------------------------------------------------------------------------------------------  401
BnbZIP68a  ------------------------------------------------------------------------------------------  381
BnbZIP68b  ------------------------------------------------------------------------------------------  383
BnbZIP16d  ------------------------------------------------------------------------------------------  300
BnbZIP16e  ------------------------------------------------------------------------------------------  153
BnbZIP41a  ------------------------------------------------------------------------------------------  313
BnbZIP41b  ------------------------------------------------------------------------------------------  313
BnbZIP41c  ------------------------------------------------------------------------------------------  313
BnbZIP41d  ------------------------------------------------------------------------------------------  313
BnbZIP54a  ------------------------------------------------------------------------------------------  365
BnbZIP54b  ------------------------------------------------------------------------------------------  363
BnbZIP55a  ------------------------------------------------------------------------------------------  443
BnbZIP55b  ------------------------------------------------------------------------------------------  414
```

```
BnbZIP17c  ------------------------------------------------------------------------------------------  601
BnbZIP28a  ------------------------------------------------------------------------------------------  595
BnbZIP28b  ------------------------------------------------------------------------------------------  635
BnbZIP49a  ------------------------------------------------------------------------------------------  464
BnbZIP49b  ------------------------------------------------------------------------------------------  438
BnbZIP9a   ------------------------------------------------------------------------------------------  278
BnbZIP9b   ------------------------------------------------------------------------------------------  278
BnbZIP9c   ------------------------------------------------------------------------------------------  278
BnbZIP9d   ------------------------------------------------------------------------------------------  278
BnbZIP10a  ------------------------------------------------------------------------------------------  395
BnbZIP10b  ------------------------------------------------------------------------------------------  394
BnbZIP25a  ------------------------------------------------------------------------------------------  315
BnbZIP25b  ------------------------------------------------------------------------------------------  384
BnbZIP25c  ------------------------------------------------------------------------------------------  378
BnbZIP25d  ------------------------------------------------------------------------------------------  192
BnbZIP25e  ------------------------------------------------------------------------------------------  372
BnbZIP25f  ------------------------------------------------------------------------------------------  183
BnbZIP63a  ------------------------------------------------------------------------------------------  319
BnbZIP63b  ------------------------------------------------------------------------------------------  315
BnbZIP63c  ------------------------------------------------------------------------------------------  314
BnbZIP63d  ------------------------------------------------------------------------------------------  318
BnbZIP20a  SGNVASYMGQMAMAMGKLGTLEGFIRQADN--------------------------------------------------------LRLQ  286
BnbZIP20b  SGNVASYMGQMAMAMGKLGTLEGFIRQADN--------------------------------------------------------LRLQ  287
BnbZIP45d  SDNVASYMGQMAMAMGQLGTLEGFIRQADN--------------------------------------------------------LRLQ  290
BnbZIP45e  SDNVASYMGQMAMAMGQLGTLEGFIRQADN--------------------------------------------------------LRLQ  290
BnbZIP45a  SDNVASYMGQMAMAMGQLGTLEGFIRQADN--------------------------------------------------------LRLQ  293
BnbZIP45b  SDNVASYMGQMAMAMGQLGTLEGFIRQADN--------------------------------------------------------LRLQ  290
BnbZIP45c  SDNVASYMGQMAMAMGQLGTLEGFIRQADN--------------------------------------------------------LRLQ  292
BnbZIP45f  SDNVASYMGQMAMAMGQLGTLEGFIRQVH-------------------------------------------------------------  308
BnbZIP26a  SGNVANYMGQMAMAMGKLGTLEGFIRQADN--------------------------------------------------------LRLQ  285
BnbZIP26b  SGNVANYMGQMAMAMGKLGTLEGFLRQADN--------------------------------------------------------LRLQ  285
BnbZIP21a  --PVMDGMQQMAVALGKISNLEGFIRQADN--------------------------------------------------------LRQQ  409
BnbZIP21b  --PVMDGMQQMAVALGKISNLEGFIRQADN--------------------------------------------------------LRQQ  409
BnbZIP21c  --QVIDGMQQMAVALGKVSNLEGFIRQADN--------------------------------------------------------LRQQ  415
BnbZIP21d  --QVIDGMQQMAVALGKVSNLEGFIRQADN--------------------------------------------------------LRQQ  359
BnbZIP22a  VESVSHG-AQMASAMENLQALEGFVNQAD--------------------------------------------------------HLRKQ  342
BnbZIP22b  VESVSHG-AQMASAMENLQALEGFVNQAD--------------------------------------------------------HLRKQ  375
BnbZIP22c  VESLNHGGAQMASAMENLESLEGFVNQAD--------------------------------------------------------HLRKQ  329
BnbZIP22d  VESLNHGGAQMASAMENLESLEGFVNQAD--------------------------------------------------------HLRKQ  328
BnbZIP50c  VMDSNDYPSHMGAAVENLQALEGFVNQAD--------------------------------------------------------HLRQQ  328
BnbZIP50d  VMESNDYPSHMGAAVENLQALEGFVNQVSGSFEATNIAANGKDLDDKTSSSRSKDSNFWFGVRQRKTLVRAASSWSEGKSPYDTLELERD  384
BnbZIP46a  SANVADYMGHMAMAMGKLGTLENFLRQADL--------------------------------------------------------LRQQ  401
BnbZIP46b  SANVADYMGHMAMAMGKLGTLENFLRQADL--------------------------------------------------------LRQQ  401
BnbZIP47a  RLGEGSYIPQVNSAMERLEALVSFVNQAD--------------------------------------------------------HLRHE  327
BnbZIP47b  GLGEGNYIPQVNSAMERLEALVSFVNQAD--------------------------------------------------------HLRHE  324
BnbZIP47c  RLGEGSYIPQVNSAMERLEALVSFVNQAD--------------------------------------------------------HLRHE  327
BnbZIP47d  GLGEGNYIPQVNSAMERLEALVSFVNQAD--------------------------------------------------------HLRHE  324
BnbZIP47e  RLGEGNYIPQVNSAMERAEASVSFVNQAD--------------------------------------------------------HLRHE  346
BnbZIP50b  FS--MDYPPQMAAALENLQALEGFVNQAD--------------------------------------------------------HLRQQ  387
BnbZIP50a  FS--MDYPPQMAAALENLQALEGFVNQAD--------------------------------------------------------HLRQQ  401
BnbZIP57a  NLGEGSYIPQITCAMERLEALVSFVNQAD--------------------------------------------------------HLRHE  319
BnbZIP57b  NLGEGSYIPQITCAMERLEALVSFVDQAD--------------------------------------------------------HLRHE  319
BnbZIP57c  ELGEGSYVPQITSAMERLEALVSFVNQAD--------------------------------------------------------HLRHE  321
BnbZIP57d  ELGEGSYVPQITSAMERLEALVSFVDQAD--------------------------------------------------------HLRHE  321
BnbZIP65a  PPHLSNFMSHMSLALNKLSSLEGFVLQADN--------------------------------------------------------LRHQ  408
BnbZIP65b  PPHLSNFMSHMSLALNKLSSLEGFVLQADN--------------------------------------------------------LRHQ  408
BnbZIP65c  PPHLSNFMSHMSLALNKLSALEGFVLQVP-------------------------------------------------------------  328
BnbZIP34a  ------------------------------------------------------------------------------------------  268
BnbZIP34b  ------------------------------------------------------------------------------------------  268
BnbZIP34c  ------------------------------------------------------------------------------------------  273
BnbZIP34d  ------------------------------------------------------------------------------------------  280
BnbZIP34e  ------------------------------------------------------------------------------------------  279
BnbZIP61a  ------------------------------------------------------------------------------------------  298
BnbZIP61b  ------------------------------------------------------------------------------------------  267
BnbZIP61c  ------------------------------------------------------------------------------------------  284
BnbZIP19a  --------HKAA------------------------------------------------------------------------------  255
BnbZIP19b  --------HRGPKGA---------------------------------------------------------------------------  271
BnbZIP19c  --------HRGPKGA---------------------------------------------------------------------------  238
BnbZIP23b  --------TCAAKAV---------------------------------------------------------------------------  239
BnbZIP23a  --------TCAAKAV---------------------------------------------------------------------------  241
BnbZIP23c  --------TPASKAV---------------------------------------------------------------------------  248
BnbZIP23d  --------TPAAKAV---------------------------------------------------------------------------  239
BnbZIP24a  ------------------------------------------------------------------------------------------  188
BnbZIP24b  ------------------------------------------------------------------------------------------  188
BnbZIP16a  ------------------------------------------------------------------------------------------  459
BnbZIP16b  ------------------------------------------------------------------------------------------  395
BnbZIP16c  ------------------------------------------------------------------------------------------  401
BnbZIP68a  ------------------------------------------------------------------------------------------  381
BnbZIP68b  ------------------------------------------------------------------------------------------  383
BnbZIP16d  ------------------------------------------------------------------------------------------  300
BnbZIP16e  ------------------------------------------------------------------------------------------  153
BnbZIP41a  ------------------------------------------------------------------------------------------  313
BnbZIP41b  ------------------------------------------------------------------------------------------  313
BnbZIP41c  ------------------------------------------------------------------------------------------  313
BnbZIP41d  ------------------------------------------------------------------------------------------  313
BnbZIP54a  ------------------------------------------------------------------------------------------  365
BnbZIP54b  ------------------------------------------------------------------------------------------  363
BnbZIP55a  ------------------------------------------------------------------------------------------  443
BnbZIP55b  ------------------------------------------------------------------------------------------  414
BnbZIP55c  ------------------------------------------------------------------------------------------  300
BnbZIP56a  ------------------------------------------------------------------------------------------  164
BnbZIP56b  ------------------------------------------------------------------------------------------  164
BnbZIP56c  ------------------------------------------------------------------------------------------  166
BnbZIP56d  ------------------------------------------------------------------------------------------  162
BnbZIP64a  ------------------------------------------------------------------------------------------  150
BnbZIP64b  ------------------------------------------------------------------------------------------  150
BnbZIP64c  ------------------------------------------------------------------------------------------  175
BnbZIP18a  ------------------------------------------------------------------------------------------  356
BnbZIP18b  ------------------------------------------------------------------------------------------  355
BnbZIP18c  ------------------------------------------------------------------------------------------  352
BnbZIP18d  ------------------------------------------------------------------------------------------  343
BnbZIP29a  ------------------------------------------------------------------------------------------  445
BnbZIP29b  ------------------------------------------------------------------------------------------  441
BnbZIP29c  ------------------------------------------------------------------------------------------  426
BnbZIP29d  ------------------------------------------------------------------------------------------  438
BnbZIP30a  ------------------------------------------------------------------------------------------  444
BnbZIP30b  ------------------------------------------------------------------------------------------  442
BnbZIP51a  ------------------------------------------------------------------------------------------  330
BnbZIP51b  ------------------------------------------------------------------------------------------  322
BnbZIP51c  ------------------------------------------------------------------------------------------  241
BnbZIP69d  N-----------------------------------------------------------------------------------------  392
BnbZIP52a  ------------------------------------------------------------------------------------------  328
BnbZIP52b  ------------------------------------------------------------------------------------------  325
BnbZIP52c  ------------------------------------------------------------------------------------------  321
BnbZIP52d  ------------------------------------------------------------------------------------------  318
BnbZIP59a  ------------------------------------------------------------------------------------------  368
BnbZIP59b  ------------------------------------------------------------------------------------------  371
BnbZIP59c  ------------------------------------------------------------------------------------------  370
BnbZIP59d  ------------------------------------------------------------------------------------------  318
BnbZIP69a  D-----------------------------------------------------------------------------------------  393
BnbZIP69b  D-----------------------------------------------------------------------------------------  393
```

BnbZIP21b	TVHQLRRILTVR-----------------------------QAARCFLVIG----------------------EYYGRLR----------	438
BnbZIP21c	TVHQLRRILTVR-----------------------------QAARCFLVIG----------------------EYYGRLR----------	444
BnbZIP21d	TVHQLRQILTVR-----------------------------QAARCFLLIG----------------------EYYGRLR----------	388
BnbZIP22a	TLQQMSKILTTR-----------------------------QAARGLLALG----------------------EYFHRLR----------	371
BnbZIP22b	TLQQMSKILTTR-----------------------------QAARGLLALG----------------------EYFHRLR----------	404
BnbZIP22c	SLQQMSKVLTTR-----------------------------QAARGLLALG----------------------EYFHRLR----------	358
BnbZIP22d	SLQQMSKVLTTR-----------------------------QAARGLLALG----------------------EYFHRLR----------	357
BnbZIP50c	TLQQMAKILTTR-----------------------------QAARGLLCLG----------------------EYLHRLR----------	357
BnbZIP50d	AEEEQIKVAYRRLAKYYHPDVYDGKGTLEEGETAESRFIKIQAAYELLMDTEKRRQYDTDNRVNPMKASQAWMEWLMKKRKAFDQRGDMA	474
BnbZIP46a	TLQQLHRILTTR-----------------------------QAARAFLVIH----------------------DYICRLR----------	430
BnbZIP46b	TLQQLHRILTTR-----------------------------QAARAFLVIH----------------------DYICRLR----------	430
BnbZIP47a	TLQQMHRILTTR-----------------------------QAARGLLALG----------------------EYFQRLR----------	356
BnbZIP47b	TLQQMHRILTTR-----------------------------QAARGLLALG----------------------EYFQRLR----------	353
BnbZIP47c	TLQQMHRILTTR-----------------------------QAARGLLALG----------------------EYFQRLR----------	356
BnbZIP47d	TLQQMHRILTTR-----------------------------QAARGLMALG----------------------EYFQRLR----------	353
BnbZIP47e	TLQQMHRILTTR-----------------------------QAARGLLALG----------------------EYFQRLR----------	375
BnbZIP50b	TLQQMAKILTTR-----------------------------QSARGLLALG----------------------EYLHRLR----------	416
BnbZIP50a	TLQQMAKILTTR-----------------------------QSARGLLALG----------------------EYLHRLR----------	430
BnbZIP57a	TLQQMHRILTTR-----------------------------QAARGLLALG----------------------EYFQRLR----------	348
BnbZIP57b	TLQQMHRILTTR-----------------------------QAARGLLALG----------------------EYFQRLR----------	348
BnbZIP57c	TLQQMHRILTTR-----------------------------QAARGLLALG----------------------EYFQRLR----------	350
BnbZIP57d	TLQQMHRILTTR-----------------------------QAARGLLALG----------------------EYFQRLR----------	350
BnbZIP65a	TIHRLNQLLTTR-----------------------------QEARCILAVA----------------------EYFHRLQ----------	437
BnbZIP65b	TIHRLNQLLTTR-----------------------------QEARCILAVA----------------------EYFHRLQ----------	437
BnbZIP65c	----SNPFLLFK-----------------------------TEVYTKLYQS----------------------LYIKYI-----------	352
BnbZIP34a	--	268
BnbZIP34b	--	268
BnbZIP34c	--	273
BnbZIP34d	--	280
BnbZIP34e	--	279
BnbZIP61a	--	298
BnbZIP61b	--	267
BnbZIP61c	--	284
BnbZIP19a	--	255
BnbZIP19b	--	271
BnbZIP19c	--	238
BnbZIP23b	--	239
BnbZIP23a	--	241
BnbZIP23c	--	248
BnbZIP23d	--	239
BnbZIP24a	--	188
BnbZIP24b	--	188
BnbZIP16a	--	459
BnbZIP16b	--	395
BnbZIP16c	--	401
BnbZIP68a	--	381
BnbZIP68b	--	383
BnbZIP16d	--	300
BnbZIP16e	--	153
BnbZIP41a	--	313
BnbZIP41b	--	313
BnbZIP41c	--	313
BnbZIP41d	--	313
BnbZIP54a	--	365
BnbZIP54b	--	363
BnbZIP55a	--	443
BnbZIP55b	--	414
BnbZIP55c	--	300
BnbZIP56a	--	164
BnbZIP56b	--	164
BnbZIP56c	--	166
BnbZIP56d	--	162
BnbZIP64a	--	150
BnbZIP64b	--	150
BnbZIP64c	--	175
BnbZIP18a	--	356
BnbZIP18b	--	355
BnbZIP18c	--	352
BnbZIP18d	--	343
BnbZIP29a	--	445
BnbZIP29b	--	441
BnbZIP29c	--	426
BnbZIP29d	--	438
BnbZIP30a	--	444
BnbZIP30b	--	442
BnbZIP51a	--	330
BnbZIP51b	--	322
BnbZIP51c	--	241
BnbZIP69d	--	392
BnbZIP52a	--	328
BnbZIP52b	--	325
BnbZIP52c	--	321
BnbZIP52d	--	318
BnbZIP59a	--	368
BnbZIP59b	--	371
BnbZIP59c	--	370
BnbZIP59d	--	318
BnbZIP69a	--	393
BnbZIP69b	--	393
BnbZIP69c	--	381
BnbZIP1a	--	139
BnbZIP1b	--	142
BnbZIP1c	QIKMMVVLGELGGRDEYSLVEALKQGKVNKPVVAWVSGTCARLFKSEVQFGHAGAKSGGEMESAQAKNQALMDAGAIVPTSFEALESAIK	432
BnbZIP1d	--	142
BnbZIP2a	--	168
BnbZIP2b	--	170
BnbZIP2c	--	168
BnbZIP2d	--	155
BnbZIP3a	--	188
BnbZIP3b	--	194
BnbZIP3c	--	183
BnbZIP3d	--	190
BnbZIP3e	--	183
BnbZIP3f	--	192
BnbZIP3g	--	193
BnbZIP4a	--	149
BnbZIP5a	--	186
BnbZIP7a	--	247
BnbZIP7b	--	254
BnbZIP7c	--	246
BnbZIP7d	--	249
BnbZIP11a	--	153
BnbZIP11b	--	153
BnbZIP11c	--	154
BnbZIP11d	--	142
BnbZIP11e	--	142
BnbZIP42a	--	172
BnbZIP42b	--	172
BnbZIP42c	--	171
BnbZIP44a	--	176
BnbZIP44b	--	158

BnbZIP61c	--	284
BnbZIP19a	--	255
BnbZIP19b	--	271
BnbZIP19c	--	238
BnbZIP23b	--	239
BnbZIP23a	--	241
BnbZIP23c	--	248
BnbZIP23d	--	239
BnbZIP24a	--	188
BnbZIP24b	--	188
BnbZIP16a	--	459
BnbZIP16b	--	395
BnbZIP16c	--	401
BnbZIP68a	--	381
BnbZIP68b	--	383
BnbZIP16d	--	300
BnbZIP16e	--	153
BnbZIP41a	--	313
BnbZIP41b	--	313
BnbZIP41c	--	313
BnbZIP41d	--	313
BnbZIP54a	--	365
BnbZIP54b	--	363
BnbZIP55a	--	443
BnbZIP55b	--	414
BnbZIP55c	--	300
BnbZIP56a	--	164
BnbZIP56b	--	164
BnbZIP56c	--	166
BnbZIP56d	--	162
BnbZIP64a	--	150
BnbZIP64b	--	150
BnbZIP64c	--	175
BnbZIP18a	--	356
BnbZIP18b	--	355
BnbZIP18c	--	352
BnbZIP18d	--	343
BnbZIP29a	--	445
BnbZIP29b	--	441
BnbZIP29c	--	426
BnbZIP29d	--	438
BnbZIP30a	--	444
BnbZIP30b	--	442
BnbZIP51a	--	330
BnbZIP51b	--	322
BnbZIP51c	--	241
BnbZIP69d	--	392
BnbZIP52a	--	328
BnbZIP52b	--	325
BnbZIP52c	--	321
BnbZIP52d	--	318
BnbZIP59a	--	368
BnbZIP59b	--	371
BnbZIP59c	--	370
BnbZIP59d	--	318
BnbZIP69a	--	393
BnbZIP69b	--	393
BnbZIP69c	--	381
BnbZIP1a	--	139
BnbZIP1b	--	142
BnbZIP1c	E T F E K L V E E G K V S P I K E V T P P Q I P E D L S S A I K S G K V R A P T H I I S T I S D D R G E E P C Y A G V P M S S I I E Q G L G V G D V I S L L W F K R S L P R Y C T K	522
BnbZIP1d	--	142
BnbZIP2a	--	168
BnbZIP2b	--	170
BnbZIP2c	--	168
BnbZIP2d	--	155
BnbZIP3a	--	188
BnbZIP3b	--	194
BnbZIP3c	--	183
BnbZIP3d	--	190
BnbZIP3e	--	183
BnbZIP3f	--	192
BnbZIP3g	--	193
BnbZIP4a	--	149
BnbZIP5a	--	186
BnbZIP7a	--	247
BnbZIP7b	--	254
BnbZIP7c	--	246
BnbZIP7d	--	249
BnbZIP11a	--	153
BnbZIP11b	--	153
BnbZIP11c	--	154
BnbZIP11d	--	142
BnbZIP11e	--	142
BnbZIP42a	--	172
BnbZIP42b	--	172
BnbZIP42c	--	171
BnbZIP44a	--	176
BnbZIP44b	--	158
BnbZIP44c	--	158
BnbZIP44d	--	179
BnbZIP48a	--	170
BnbZIP48b	--	170
BnbZIP48c	--	179
BnbZIP48d	--	179
BnbZIP48e	--	176
BnbZIP48f	--	176
BnbZIP53a	--	141
BnbZIP53b	--	141
BnbZIP53c	--	134
BnbZIP53d	--	134
BnbZIP58a	--	198
BnbZIP58b	--	198
BnbZIP58c	--	198
BnbZIP58d	--	163
BnbZIP60a	--	233
BnbZIP60b	--	242
BnbZIP62a	--	462
BnbZIP62b	--	459
BnbZIP62c	--	150
BnbZIP36a	--	302
BnbZIP36b	--	335
BnbZIP13a	--	286
BnbZIP13b	--	266
BnbZIP8a	--	120
BnbZIP70a	--	221
BnbZIP70b	--	218
BnbZIP70c	--	183
BnbZIP70d	--	177
BnbZIP72a	--	149
BnbZIP72b	--	149

```
BnbZIP64c  ------------------------------------------------------------------------------------------  175
BnbZIP18a  ------------------------------------------------------------------------------------------  356
BnbZIP18b  ------------------------------------------------------------------------------------------  355
BnbZIP18c  ------------------------------------------------------------------------------------------  352
BnbZIP18d  ------------------------------------------------------------------------------------------  343
BnbZIP29a  ------------------------------------------------------------------------------------------  445
BnbZIP29b  ------------------------------------------------------------------------------------------  441
BnbZIP29c  ------------------------------------------------------------------------------------------  426
BnbZIP29d  ------------------------------------------------------------------------------------------  438
BnbZIP30a  ------------------------------------------------------------------------------------------  444
BnbZIP30b  ------------------------------------------------------------------------------------------  442
BnbZIP51a  ------------------------------------------------------------------------------------------  330
BnbZIP51b  ------------------------------------------------------------------------------------------  322
BnbZIP51c  ------------------------------------------------------------------------------------------  241
BnbZIP69d  ------------------------------------------------------------------------------------------  392
BnbZIP52a  ------------------------------------------------------------------------------------------  328
BnbZIP52b  ------------------------------------------------------------------------------------------  325
BnbZIP52c  ------------------------------------------------------------------------------------------  321
BnbZIP52d  ------------------------------------------------------------------------------------------  318
BnbZIP59a  ------------------------------------------------------------------------------------------  368
BnbZIP59b  ------------------------------------------------------------------------------------------  371
BnbZIP59c  ------------------------------------------------------------------------------------------  370
BnbZIP59d  ------------------------------------------------------------------------------------------  318
BnbZIP69a  ------------------------------------------------------------------------------------------  393
BnbZIP69b  ------------------------------------------------------------------------------------------  393
BnbZIP69c  ------------------------------------------------------------------------------------------  381
BnbZIP1a   ------------------------------------------------------------------------------------------  139
BnbZIP1b   ------------------------------------------------------------------------------------------  142
BnbZIP1c   FIEICIMLCADHGPCVSGAHNTIVTARAGKDLVSSLVSGLLTIGPRFGGAIDDAARYFKDACDRNLTPYEFVEGMKKKGIRVPGIGHRIK  612
BnbZIP1d   ------------------------------------------------------------------------------------------  142
BnbZIP2a   ------------------------------------------------------------------------------------------  168
BnbZIP2b   ------------------------------------------------------------------------------------------  170
BnbZIP2c   ------------------------------------------------------------------------------------------  168
BnbZIP2d   ------------------------------------------------------------------------------------------  155
BnbZIP3a   ------------------------------------------------------------------------------------------  188
BnbZIP3b   ------------------------------------------------------------------------------------------  194
BnbZIP3c   ------------------------------------------------------------------------------------------  183
BnbZIP3d   ------------------------------------------------------------------------------------------  190
BnbZIP3e   ------------------------------------------------------------------------------------------  183
BnbZIP3f   ------------------------------------------------------------------------------------------  192
BnbZIP3g   ------------------------------------------------------------------------------------------  193
BnbZIP4a   ------------------------------------------------------------------------------------------  149
BnbZIP5a   ------------------------------------------------------------------------------------------  186
BnbZIP7a   ------------------------------------------------------------------------------------------  247
BnbZIP7b   ------------------------------------------------------------------------------------------  254
BnbZIP7c   ------------------------------------------------------------------------------------------  246
BnbZIP7d   ------------------------------------------------------------------------------------------  249
BnbZIP11a  ------------------------------------------------------------------------------------------  153
BnbZIP11b  ------------------------------------------------------------------------------------------  153
BnbZIP11c  ------------------------------------------------------------------------------------------  154
BnbZIP11d  ------------------------------------------------------------------------------------------  142
BnbZIP11e  ------------------------------------------------------------------------------------------  142
BnbZIP42a  ------------------------------------------------------------------------------------------  172
BnbZIP42b  ------------------------------------------------------------------------------------------  172
BnbZIP42c  ------------------------------------------------------------------------------------------  171
BnbZIP44a  ------------------------------------------------------------------------------------------  176
BnbZIP44b  ------------------------------------------------------------------------------------------  158
BnbZIP44c  ------------------------------------------------------------------------------------------  158
BnbZIP44d  ------------------------------------------------------------------------------------------  179
BnbZIP48a  ------------------------------------------------------------------------------------------  170
BnbZIP48b  ------------------------------------------------------------------------------------------  170
BnbZIP48c  ------------------------------------------------------------------------------------------  179
BnbZIP48d  ------------------------------------------------------------------------------------------  179
BnbZIP48e  ------------------------------------------------------------------------------------------  176
BnbZIP48f  ------------------------------------------------------------------------------------------  176
BnbZIP53a  ------------------------------------------------------------------------------------------  141
BnbZIP53b  ------------------------------------------------------------------------------------------  141
BnbZIP53c  ------------------------------------------------------------------------------------------  134
BnbZIP53d  ------------------------------------------------------------------------------------------  134
BnbZIP58a  ------------------------------------------------------------------------------------------  198
BnbZIP58b  ------------------------------------------------------------------------------------------  198
BnbZIP58c  ------------------------------------------------------------------------------------------  198
BnbZIP58d  ------------------------------------------------------------------------------------------  163
BnbZIP60a  ------------------------------------------------------------------------------------------  233
BnbZIP60b  ------------------------------------------------------------------------------------------  242
BnbZIP62a  ------------------------------------------------------------------------------------------  462
BnbZIP62b  ------------------------------------------------------------------------------------------  459
BnbZIP62c  ------------------------------------------------------------------------------------------  150
BnbZIP36a  ------------------------------------------------------------------------------------------  302
BnbZIP36b  ------------------------------------------------------------------------------------------  335
BnbZIP13a  ------------------------------------------------------------------------------------------  286
BnbZIP13b  ------------------------------------------------------------------------------------------  266
BnbZIP8a   ------------------------------------------------------------------------------------------  120
BnbZIP70a  ------------------------------------------------------------------------------------------  221
BnbZIP70b  ------------------------------------------------------------------------------------------  218
BnbZIP70c  ------------------------------------------------------------------------------------------  183
BnbZIP70d  ------------------------------------------------------------------------------------------  177
BnbZIP72a  ------------------------------------------------------------------------------------------  149
BnbZIP72b  ------------------------------------------------------------------------------------------  149
BnbZIP27c  ------------------------------------------------------------------------------------------  180
BnbZIP15a  ------------------------------------------------------------------------------------------  391
BnbZIP15b  ------------------------------------------------------------------------------------------  395
BnbZIP35a  ------------------------------------------------------------------------------------------  237
BnbZIP35b  ------------------------------------------------------------------------------------------  330
BnbZIP47f  ------------------------------------------------------------------------------------------  372
BnbZIP19d  ------------------------------------------------------------------------------------------  273
BnbZIP16f  ------------------------------------------------------------------------------------------  82
BnbZIP64d  ------------------------------------------------------------------------------------------  150
BnbZIP64e  ------------------------------------------------------------------------------------------  133
BnbZIP18e  ------------------------------------------------------------------------------------------  334
BnbZIP18f  ------------------------------------------------------------------------------------------  339
BnbZIP30c  ------------------------------------------------------------------------------------------  307
BnbZIP30d  ------------------------------------------------------------------------------------------  264
BnbZIP30e  ------------------------------------------------------------------------------------------  372
BnbZIP2e   ------------------------------------------------------------------------------------------  168
BnbZIP3h   ------------------------------------------------------------------------------------------  162
BnbZIP4b   ------------------------------------------------------------------------------------------  149
BnbZIP5b   ------------------------------------------------------------------------------------------  195
BnbZIP5c   ------------------------------------------------------------------------------------------  204
BnbZIP5d   ------------------------------------------------------------------------------------------  168
BnbZIP8b   ------------------------------------------------------------------------------------------  120
BnbZIP11f  ------------------------------------------------------------------------------------------  154
BnbZIP11g  ------------------------------------------------------------------------------------------  142
BnbZIP75a  ------------------------------------------------------------------------------------------  170
BnbZIP75b  ------------------------------------------------------------------------------------------  164

                    1270      1280      1290      1300      1310      1320      1330      1340      1350

BnbZIP12a  ------------------------------------------------------------------------------------------  274
BnbZIP12b  ------------------------------------------------------------------------------------------  282
BnbZIP12c  ------------------------------------------------------------------------------------------  276
```

```
BnbZIP2c    - - - - - - - - - - - - - - - - - - - - - - - - - - - - - - - - - - - - - - - - - - - - - - - - - - - - - - - - - - - - - - - - - - - - - - - - - - - - - - - - - - - - - - - - - -  168
BnbZIP2d    - - - - - - - - - - - - - - - - - - - - - - - - - - - - - - - - - - - - - - - - - - - - - - - - - - - - - - - - - - - - - - - - - - - - - - - - - - - - - - - - - - - - - - - - - -  155
BnbZIP3a    - - - - - - - - - - - - - - - - - - - - - - - - - - - - - - - - - - - - - - - - - - - - - - - - - - - - - - - - - - - - - - - - - - - - - - - - - - - - - - - - - - - - - - - - - -  188
BnbZIP3b    - - - - - - - - - - - - - - - - - - - - - - - - - - - - - - - - - - - - - - - - - - - - - - - - - - - - - - - - - - - - - - - - - - - - - - - - - - - - - - - - - - - - - - - - - -  194
BnbZIP3c    - - - - - - - - - - - - - - - - - - - - - - - - - - - - - - - - - - - - - - - - - - - - - - - - - - - - - - - - - - - - - - - - - - - - - - - - - - - - - - - - - - - - - - - - - -  183
BnbZIP3d    - - - - - - - - - - - - - - - - - - - - - - - - - - - - - - - - - - - - - - - - - - - - - - - - - - - - - - - - - - - - - - - - - - - - - - - - - - - - - - - - - - - - - - - - - -  190
BnbZIP3e    - - - - - - - - - - - - - - - - - - - - - - - - - - - - - - - - - - - - - - - - - - - - - - - - - - - - - - - - - - - - - - - - - - - - - - - - - - - - - - - - - - - - - - - - - -  183
BnbZIP3f    - - - - - - - - - - - - - - - - - - - - - - - - - - - - - - - - - - - - - - - - - - - - - - - - - - - - - - - - - - - - - - - - - - - - - - - - - - - - - - - - - - - - - - - - - -  192
BnbZIP3g    - - - - - - - - - - - - - - - - - - - - - - - - - - - - - - - - - - - - - - - - - - - - - - - - - - - - - - - - - - - - - - - - - - - - - - - - - - - - - - - - - - - - - - - - - -  193
BnbZIP4a    - - - - - - - - - - - - - - - - - - - - - - - - - - - - - - - - - - - - - - - - - - - - - - - - - - - - - - - - - - - - - - - - - - - - - - - - - - - - - - - - - - - - - - - - - -  149
BnbZIP5a    - - - - - - - - - - - - - - - - - - - - - - - - - - - - - - - - - - - - - - - - - - - - - - - - - - - - - - - - - - - - - - - - - - - - - - - - - - - - - - - - - - - - - - - - - -  186
BnbZIP7a    - - - - - - - - - - - - - - - - - - - - - - - - - - - - - - - - - - - - - - - - - - - - - - - - - - - - - - - - - - - - - - - - - - - - - - - - - - - - - - - - - - - - - - - - - -  247
BnbZIP7b    - - - - - - - - - - - - - - - - - - - - - - - - - - - - - - - - - - - - - - - - - - - - - - - - - - - - - - - - - - - - - - - - - - - - - - - - - - - - - - - - - - - - - - - - - -  254
BnbZIP7c    - - - - - - - - - - - - - - - - - - - - - - - - - - - - - - - - - - - - - - - - - - - - - - - - - - - - - - - - - - - - - - - - - - - - - - - - - - - - - - - - - - - - - - - - - -  246
BnbZIP7d    - - - - - - - - - - - - - - - - - - - - - - - - - - - - - - - - - - - - - - - - - - - - - - - - - - - - - - - - - - - - - - - - - - - - - - - - - - - - - - - - - - - - - - - - - -  249
BnbZIP11a   - - - - - - - - - - - - - - - - - - - - - - - - - - - - - - - - - - - - - - - - - - - - - - - - - - - - - - - - - - - - - - - - - - - - - - - - - - - - - - - - - - - - - - - - - -  153
BnbZIP11b   - - - - - - - - - - - - - - - - - - - - - - - - - - - - - - - - - - - - - - - - - - - - - - - - - - - - - - - - - - - - - - - - - - - - - - - - - - - - - - - - - - - - - - - - - -  153
BnbZIP11c   - - - - - - - - - - - - - - - - - - - - - - - - - - - - - - - - - - - - - - - - - - - - - - - - - - - - - - - - - - - - - - - - - - - - - - - - - - - - - - - - - - - - - - - - - -  154
BnbZIP11d   - - - - - - - - - - - - - - - - - - - - - - - - - - - - - - - - - - - - - - - - - - - - - - - - - - - - - - - - - - - - - - - - - - - - - - - - - - - - - - - - - - - - - - - - - -  142
BnbZIP11e   - - - - - - - - - - - - - - - - - - - - - - - - - - - - - - - - - - - - - - - - - - - - - - - - - - - - - - - - - - - - - - - - - - - - - - - - - - - - - - - - - - - - - - - - - -  142
BnbZIP42a   - - - - - - - - - - - - - - - - - - - - - - - - - - - - - - - - - - - - - - - - - - - - - - - - - - - - - - - - - - - - - - - - - - - - - - - - - - - - - - - - - - - - - - - - - -  172
BnbZIP42b   - - - - - - - - - - - - - - - - - - - - - - - - - - - - - - - - - - - - - - - - - - - - - - - - - - - - - - - - - - - - - - - - - - - - - - - - - - - - - - - - - - - - - - - - - -  172
BnbZIP42c   - - - - - - - - - - - - - - - - - - - - - - - - - - - - - - - - - - - - - - - - - - - - - - - - - - - - - - - - - - - - - - - - - - - - - - - - - - - - - - - - - - - - - - - - - -  171
BnbZIP44a   - - - - - - - - - - - - - - - - - - - - - - - - - - - - - - - - - - - - - - - - - - - - - - - - - - - - - - - - - - - - - - - - - - - - - - - - - - - - - - - - - - - - - - - - - -  176
BnbZIP44b   - - - - - - - - - - - - - - - - - - - - - - - - - - - - - - - - - - - - - - - - - - - - - - - - - - - - - - - - - - - - - - - - - - - - - - - - - - - - - - - - - - - - - - - - - -  158
BnbZIP44c   - - - - - - - - - - - - - - - - - - - - - - - - - - - - - - - - - - - - - - - - - - - - - - - - - - - - - - - - - - - - - - - - - - - - - - - - - - - - - - - - - - - - - - - - - -  158
BnbZIP44d   - - - - - - - - - - - - - - - - - - - - - - - - - - - - - - - - - - - - - - - - - - - - - - - - - - - - - - - - - - - - - - - - - - - - - - - - - - - - - - - - - - - - - - - - - -  179
BnbZIP48a   - - - - - - - - - - - - - - - - - - - - - - - - - - - - - - - - - - - - - - - - - - - - - - - - - - - - - - - - - - - - - - - - - - - - - - - - - - - - - - - - - - - - - - - - - -  170
BnbZIP48b   - - - - - - - - - - - - - - - - - - - - - - - - - - - - - - - - - - - - - - - - - - - - - - - - - - - - - - - - - - - - - - - - - - - - - - - - - - - - - - - - - - - - - - - - - -  170
BnbZIP48c   - - - - - - - - - - - - - - - - - - - - - - - - - - - - - - - - - - - - - - - - - - - - - - - - - - - - - - - - - - - - - - - - - - - - - - - - - - - - - - - - - - - - - - - - - -  179
BnbZIP48d   - - - - - - - - - - - - - - - - - - - - - - - - - - - - - - - - - - - - - - - - - - - - - - - - - - - - - - - - - - - - - - - - - - - - - - - - - - - - - - - - - - - - - - - - - -  179
BnbZIP48e   - - - - - - - - - - - - - - - - - - - - - - - - - - - - - - - - - - - - - - - - - - - - - - - - - - - - - - - - - - - - - - - - - - - - - - - - - - - - - - - - - - - - - - - - - -  176
BnbZIP48f   - - - - - - - - - - - - - - - - - - - - - - - - - - - - - - - - - - - - - - - - - - - - - - - - - - - - - - - - - - - - - - - - - - - - - - - - - - - - - - - - - - - - - - - - - -  176
BnbZIP53a   - - - - - - - - - - - - - - - - - - - - - - - - - - - - - - - - - - - - - - - - - - - - - - - - - - - - - - - - - - - - - - - - - - - - - - - - - - - - - - - - - - - - - - - - - -  141
BnbZIP53b   - - - - - - - - - - - - - - - - - - - - - - - - - - - - - - - - - - - - - - - - - - - - - - - - - - - - - - - - - - - - - - - - - - - - - - - - - - - - - - - - - - - - - - - - - -  141
BnbZIP53c   - - - - - - - - - - - - - - - - - - - - - - - - - - - - - - - - - - - - - - - - - - - - - - - - - - - - - - - - - - - - - - - - - - - - - - - - - - - - - - - - - - - - - - - - - -  134
BnbZIP53d   - - - - - - - - - - - - - - - - - - - - - - - - - - - - - - - - - - - - - - - - - - - - - - - - - - - - - - - - - - - - - - - - - - - - - - - - - - - - - - - - - - - - - - - - - -  134
BnbZIP58a   - - - - - - - - - - - - - - - - - - - - - - - - - - - - - - - - - - - - - - - - - - - - - - - - - - - - - - - - - - - - - - - - - - - - - - - - - - - - - - - - - - - - - - - - - -  198
BnbZIP58b   - - - - - - - - - - - - - - - - - - - - - - - - - - - - - - - - - - - - - - - - - - - - - - - - - - - - - - - - - - - - - - - - - - - - - - - - - - - - - - - - - - - - - - - - - -  198
BnbZIP58c   - - - - - - - - - - - - - - - - - - - - - - - - - - - - - - - - - - - - - - - - - - - - - - - - - - - - - - - - - - - - - - - - - - - - - - - - - - - - - - - - - - - - - - - - - -  198
BnbZIP58d   - - - - - - - - - - - - - - - - - - - - - - - - - - - - - - - - - - - - - - - - - - - - - - - - - - - - - - - - - - - - - - - - - - - - - - - - - - - - - - - - - - - - - - - - - -  163
BnbZIP60a   - - - - - - - - - - - - - - - - - - - - - - - - - - - - - - - - - - - - - - - - - - - - - - - - - - - - - - - - - - - - - - - - - - - - - - - - - - - - - - - - - - - - - - - - - -  233
BnbZIP60b   - - - - - - - - - - - - - - - - - - - - - - - - - - - - - - - - - - - - - - - - - - - - - - - - - - - - - - - - - - - - - - - - - - - - - - - - - - - - - - - - - - - - - - - - - -  242
BnbZIP62a   - - - - - - - - - - - - - - - - - - - - - - - - - - - - - - - - - - - - - - - - - - - - - - - - - - - - - - - - - - - - - - - - - - - - - - - - - - - - - - - - - - - - - - - - - -  462
BnbZIP62b   - - - - - - - - - - - - - - - - - - - - - - - - - - - - - - - - - - - - - - - - - - - - - - - - - - - - - - - - - - - - - - - - - - - - - - - - - - - - - - - - - - - - - - - - - -  459
BnbZIP62c   - - - - - - - - - - - - - - - - - - - - - - - - - - - - - - - - - - - - - - - - - - - - - - - - - - - - - - - - - - - - - - - - - - - - - - - - - - - - - - - - - - - - - - - - - -  150
BnbZIP36a   - - - - - - - - - - - - - - - - - - - - - - - - - - - - - - - - - - - - - - - - - - - - - - - - - - - - - - - - - - - - - - - - - - - - - - - - - - - - - - - - - - - - - - - - - -  302
BnbZIP36b   - - - - - - - - - - - - - - - - - - - - - - - - - - - - - - - - - - - - - - - - - - - - - - - - - - - - - - - - - - - - - - - - - - - - - - - - - - - - - - - - - - - - - - - - - -  335
BnbZIP13a   - - - - - - - - - - - - - - - - - - - - - - - - - - - - - - - - - - - - - - - - - - - - - - - - - - - - - - - - - - - - - - - - - - - - - - - - - - - - - - - - - - - - - - - - - -  286
BnbZIP13b   - - - - - - - - - - - - - - - - - - - - - - - - - - - - - - - - - - - - - - - - - - - - - - - - - - - - - - - - - - - - - - - - - - - - - - - - - - - - - - - - - - - - - - - - - -  266
BnbZIP8a    - - - - - - - - - - - - - - - - - - - - - - - - - - - - - - - - - - - - - - - - - - - - - - - - - - - - - - - - - - - - - - - - - - - - - - - - - - - - - - - - - - - - - - - - - -  120
BnbZIP70a   - - - - - - - - - - - - - - - - - - - - - - - - - - - - - - - - - - - - - - - - - - - - - - - - - - - - - - - - - - - - - - - - - - - - - - - - - - - - - - - - - - - - - - - - - -  221
BnbZIP70b   - - - - - - - - - - - - - - - - - - - - - - - - - - - - - - - - - - - - - - - - - - - - - - - - - - - - - - - - - - - - - - - - - - - - - - - - - - - - - - - - - - - - - - - - - -  218
BnbZIP70c   - - - - - - - - - - - - - - - - - - - - - - - - - - - - - - - - - - - - - - - - - - - - - - - - - - - - - - - - - - - - - - - - - - - - - - - - - - - - - - - - - - - - - - - - - -  183
BnbZIP70d   - - - - - - - - - - - - - - - - - - - - - - - - - - - - - - - - - - - - - - - - - - - - - - - - - - - - - - - - - - - - - - - - - - - - - - - - - - - - - - - - - - - - - - - - - -  177
BnbZIP72a   - - - - - - - - - - - - - - - - - - - - - - - - - - - - - - - - - - - - - - - - - - - - - - - - - - - - - - - - - - - - - - - - - - - - - - - - - - - - - - - - - - - - - - - - - -  149
BnbZIP72b   - - - - - - - - - - - - - - - - - - - - - - - - - - - - - - - - - - - - - - - - - - - - - - - - - - - - - - - - - - - - - - - - - - - - - - - - - - - - - - - - - - - - - - - - - -  149
BnbZIP27c   - - - - - - - - - - - - - - - - - - - - - - - - - - - - - - - - - - - - - - - - - - - - - - - - - - - - - - - - - - - - - - - - - - - - - - - - - - - - - - - - - - - - - - - - - -  180
BnbZIP15a   - - - - - - - - - - - - - - - - - - - - - - - - - - - - - - - - - - - - - - - - - - - - - - - - - - - - - - - - - - - - - - - - - - - - - - - - - - - - - - - - - - - - - - - - - -  391
BnbZIP15b   - - - - - - - - - - - - - - - - - - - - - - - - - - - - - - - - - - - - - - - - - - - - - - - - - - - - - - - - - - - - - - - - - - - - - - - - - - - - - - - - - - - - - - - - - -  395
BnbZIP35a   - - - - - - - - - - - - - - - - - - - - - - - - - - - - - - - - - - - - - - - - - - - - - - - - - - - - - - - - - - - - - - - - - - - - - - - - - - - - - - - - - - - - - - - - - -  237
BnbZIP35b   - - - - - - - - - - - - - - - - - - - - - - - - - - - - - - - - - - - - - - - - - - - - - - - - - - - - - - - - - - - - - - - - - - - - - - - - - - - - - - - - - - - - - - - - - -  330
BnbZIP47f   - - - - - - - - - - - - - - - - - - - - - - - - - - - - - - - - - - - - - - - - - - - - - - - - - - - - - - - - - - - - - - - - - - - - - - - - - - - - - - - - - - - - - - - - - -  372
BnbZIP19d   - - - - - - - - - - - - - - - - - - - - - - - - - - - - - - - - - - - - - - - - - - - - - - - - - - - - - - - - - - - - - - - - - - - - - - - - - - - - - - - - - - - - - - - - - -  273
BnbZIP16f   - - - - - - - - - - - - - - - - - - - - - - - - - - - - - - - - - - - - - - - - - - - - - - - - - - - - - - - - - - - - - - - - - - - - - - - - - - - - - - - - - - - - - - - - - -  82
BnbZIP64d   - - - - - - - - - - - - - - - - - - - - - - - - - - - - - - - - - - - - - - - - - - - - - - - - - - - - - - - - - - - - - - - - - - - - - - - - - - - - - - - - - - - - - - - - - -  150
BnbZIP64e   - - - - - - - - - - - - - - - - - - - - - - - - - - - - - - - - - - - - - - - - - - - - - - - - - - - - - - - - - - - - - - - - - - - - - - - - - - - - - - - - - - - - - - - - - -  133
BnbZIP18e   - - - - - - - - - - - - - - - - - - - - - - - - - - - - - - - - - - - - - - - - - - - - - - - - - - - - - - - - - - - - - - - - - - - - - - - - - - - - - - - - - - - - - - - - - -  334
BnbZIP18f   - - - - - - - - - - - - - - - - - - - - - - - - - - - - - - - - - - - - - - - - - - - - - - - - - - - - - - - - - - - - - - - - - - - - - - - - - - - - - - - - - - - - - - - - - -  339
BnbZIP30c   - - - - - - - - - - - - - - - - - - - - - - - - - - - - - - - - - - - - - - - - - - - - - - - - - - - - - - - - - - - - - - - - - - - - - - - - - - - - - - - - - - - - - - - - - -  307
BnbZIP30d   - - - - - - - - - - - - - - - - - - - - - - - - - - - - - - - - - - - - - - - - - - - - - - - - - - - - - - - - - - - - - - - - - - - - - - - - - - - - - - - - - - - - - - - - - -  264
BnbZIP30e   - - - - - - - - - - - - - - - - - - - - - - - - - - - - - - - - - - - - - - - - - - - - - - - - - - - - - - - - - - - - - - - - - - - - - - - - - - - - - - - - - - - - - - - - - -  372
BnbZIP2e    - - - - - - - - - - - - - - - - - - - - - - - - - - - - - - - - - - - - - - - - - - - - - - - - - - - - - - - - - - - - - - - - - - - - - - - - - - - - - - - - - - - - - - - - - -  168
BnbZIP3h    - - - - - - - - - - - - - - - - - - - - - - - - - - - - - - - - - - - - - - - - - - - - - - - - - - - - - - - - - - - - - - - - - - - - - - - - - - - - - - - - - - - - - - - - - -  162
BnbZIP4b    - - - - - - - - - - - - - - - - - - - - - - - - - - - - - - - - - - - - - - - - - - - - - - - - - - - - - - - - - - - - - - - - - - - - - - - - - - - - - - - - - - - - - - - - - -  149
BnbZIP5b    - - - - - - - - - - - - - - - - - - - - - - - - - - - - - - - - - - - - - - - - - - - - - - - - - - - - - - - - - - - - - - - - - - - - - - - - - - - - - - - - - - - - - - - - - -  195
BnbZIP5c    - - - - - - - - - - - - - - - - - - - - - - - - - - - - - - - - - - - - - - - - - - - - - - - - - - - - - - - - - - - - - - - - - - - - - - - - - - - - - - - - - - - - - - - - - -  204
BnbZIP5d    - - - - - - - - - - - - - - - - - - - - - - - - - - - - - - - - - - - - - - - - - - - - - - - - - - - - - - - - - - - - - - - - - - - - - - - - - - - - - - - - - - - - - - - - - -  168
BnbZIP8b    - - - - - - - - - - - - - - - - - - - - - - - - - - - - - - - - - - - - - - - - - - - - - - - - - - - - - - - - - - - - - - - - - - - - - - - - - - - - - - - - - - - - - - - - - -  120
BnbZIP11f   - - - - - - - - - - - - - - - - - - - - - - - - - - - - - - - - - - - - - - - - - - - - - - - - - - - - - - - - - - - - - - - - - - - - - - - - - - - - - - - - - - - - - - - - - -  154
BnbZIP11g   - - - - - - - - - - - - - - - - - - - - - - - - - - - - - - - - - - - - - - - - - - - - - - - - - - - - - - - - - - - - - - - - - - - - - - - - - - - - - - - - - - - - - - - - - -  142
BnbZIP75a   - - - - - - - - - - - - - - - - - - - - - - - - - - - - - - - - - - - - - - - - - - - - - - - - - - - - - - - - - - - - - - - - - - - - - - - - - - - - - - - - - - - - - - - - - -  170
BnbZIP75b   - - - - - - - - - - - - - - - - - - - - - - - - - - - - - - - - - - - - - - - - - - - - - - - - - - - - - - - - - - - - - - - - - - - - - - - - - - - - - - - - - - - - - - - - - -  164

                            1360                1370
            . . . . | . . . . | . . . . | . . . . | . . . . | . .
BnbZIP12a   - - - - - - - - - - - - - - - - - - - - - - - - - - -  274
BnbZIP12b   - - - - - - - - - - - - - - - - - - - - - - - - - - -  282
BnbZIP12c   - - - - - - - - - - - - - - - - - - - - - - - - - - -  276
BnbZIP12d   - - - - - - - - - - - - - - - - - - - - - - - - - - -  272
BnbZIP14a   - - - - - - - - - - - - - - - - - - - - - - - - - - -  263
BnbZIP14b   - - - - - - - - - - - - - - - - - - - - - - - - - - -  281
BnbZIP14c   - - - - - - - - - - - - - - - - - - - - - - - - - - -  243
BnbZIP14d   - - - - - - - - - - - - - - - - - - - - - - - - - - -  270
BnbZIP14e   - - - - - - - - - - - - - - - - - - - - - - - - - - -  259
BnbZIP14f   - - - - - - - - - - - - - - - - - - - - - - - - - - -  262
BnbZIP27b   - - - - - - - - - - - - - - - - - - - - - - - - - - -  231
BnbZIP27a   - - - - - - - - - - - - - - - - - - - - - - - - - - -  182
BnbZIP38e   - - - - - - - - - - - - - - - - - - - - - - - - - - -  354
BnbZIP38f   - - - - - - - - - - - - - - - - - - - - - - - - - - -  391
BnbZIP37a   - - - - - - - - - - - - - - - - - - - - - - - - - - -  410
BnbZIP37b   - - - - - - - - - - - - - - - - - - - - - - - - - - -  350
BnbZIP37c   - - - - - - - - - - - - - - - - - - - - - - - - - - -  349
BnbZIP38a   - - - - - - - - - - - - - - - - - - - - - - - - - - -  355
BnbZIP38b   - - - - - - - - - - - - - - - - - - - - - - - - - - -  354
BnbZIP38c   - - - - - - - - - - - - - - - - - - - - - - - - - - -  352
BnbZIP38d   - - - - - - - - - - - - - - - - - - - - - - - - - - -  364
BnbZIP39a   - - - - - - - - - - - - - - - - - - - - - - - - - - -  404
BnbZIP39b   - - - - - - - - - - - - - - - - - - - - - - - - - - -  394
BnbZIP39c   - - - - - - - - - - - - - - - - - - - - - - - - - - -  399
BnbZIP40a   - - - - - - - - - - - - - - - - - - - - - - - - - - -  268
BnbZIP40b   - - - - - - - - - - - - - - - - - - - - - - - - - - -  269
BnbZIP66a   - - - - - - - - - - - - - - - - - - - - - - - - - - -  290
BnbZIP66b   - - - - - - - - - - - - - - - - - - - - - - - - - - -  250
BnbZIP66c   - - - - - - - - - - - - - - - - - - - - - - - - - - -  251
BnbZIP66d   - - - - - - - - - - - - - - - - - - - - - - - - - - -  259
BnbZIP66e   - - - - - - - - - - - - - - - - - - - - - - - - - - -  271
BnbZIP66f   - - - - - - - - - - - - - - - - - - - - - - - - - - -  247
BnbZIP67a   - - - - - - - - - - - - - - - - - - - - - - - - - - -  346
BnbZIP67b   - - - - - - - - - - - - - - - - - - - - - - - - - - -  346
BnbZIP17a   - - - - - - - - - - - - - - - - - - - - - - - - - - -  605
```

```
BnbZIP48f   ---------------------------   176
BnbZIP53a   ---------------------------   141
BnbZIP53b   ---------------------------   141
BnbZIP53c   ---------------------------   134
BnbZIP53d   ---------------------------   134
BnbZIP58a   ---------------------------   198
BnbZIP58b   ---------------------------   198
BnbZIP58c   ---------------------------   198
BnbZIP58d   ---------------------------   163
BnbZIP60a   ---------------------------   233
BnbZIP60b   ---------------------------   242
BnbZIP62a   ---------------------------   462
BnbZIP62b   ---------------------------   459
BnbZIP62c   ---------------------------   150
BnbZIP36a   ---------------------------   302
BnbZIP36b   ---------------------------   335
BnbZIP13a   ---------------------------   286
BnbZIP13b   ---------------------------   266
BnbZIP8a    ---------------------------   120
BnbZIP70a   ---------------------------   221
BnbZIP70b   ---------------------------   218
BnbZIP70c   ---------------------------   183
BnbZIP70d   ---------------------------   177
BnbZIP72a   ---------------------------   149
BnbZIP72b   ---------------------------   149
BnbZIP27c   ---------------------------   180
BnbZIP15a   ---------------------------   391
BnbZIP15b   ---------------------------   395
BnbZIP35a   ---------------------------   237
BnbZIP35b   ---------------------------   330
BnbZIP47f   ---------------------------   372
BnbZIP19d   ---------------------------   273
BnbZIP16f   ---------------------------   82
BnbZIP64d   ---------------------------   150
BnbZIP64e   ---------------------------   133
BnbZIP18e   ---------------------------   334
BnbZIP18f   ---------------------------   339
BnbZIP30c   ---------------------------   307
BnbZIP30d   ---------------------------   264
BnbZIP30e   ---------------------------   372
BnbZIP2e    ---------------------------   168
BnbZIP3h    ---------------------------   162
BnbZIP4b    ---------------------------   149
BnbZIP5b    ---------------------------   195
BnbZIP5c    ---------------------------   204
BnbZIP5d    ---------------------------   168
BnbZIP8b    ---------------------------   120
BnbZIP11f   ---------------------------   154
BnbZIP11g   ---------------------------   142
BnbZIP75a   ---------------------------   170
BnbZIP75b   ---------------------------   164
```
